# Topological and Functional Properties of the Small GTPases Protein Interaction Network

**DOI:** 10.1371/journal.pone.0044882

**Published:** 2012-09-13

**Authors:** Anna Delprato

**Affiliations:** BioScience Project, Wakefield, Massachusetts, United States of America; University of Edinburgh, United Kingdom

## Abstract

Small GTP binding proteins of the Ras superfamily (Ras, Rho, Rab, Arf, and Ran) regulate key cellular processes such as signal transduction, cell proliferation, cell motility, and vesicle transport. A great deal of experimental evidence supports the existence of signaling cascades and feedback loops within and among the small GTPase subfamilies suggesting that these proteins function in a coordinated and cooperative manner. The interplay occurs largely through association with bi-partite regulatory and effector proteins but can also occur through the active form of the small GTPases themselves. In order to understand the connectivity of the small GTPases signaling routes, a systems-level approach that analyzes data describing direct and indirect interactions was used to construct the small GTPases protein interaction network. The data were curated from the Search Tool for the Retrieval of Interacting Genes (STRING) database and include only experimentally validated interactions. The network method enables the conceptualization of the overall structure as well as the underlying organization of the protein-protein interactions. The interaction network described here is comprised of 778 nodes and 1943 edges and has a scale-free topology. Rac1, Cdc42, RhoA, and HRas are identified as the hubs. Ten sub-network motifs are also identified in this study with themes in apoptosis, cell growth/proliferation, vesicle traffic, cell adhesion/junction dynamics, the nicotinamide adenine dinucleotide phosphate (NADPH) oxidase response, transcription regulation, receptor-mediated endocytosis, gene silencing, and growth factor signaling. Bottleneck proteins that bridge signaling paths and proteins that overlap in multiple small GTPase networks are described along with the functional annotation of all proteins in the network.

## Introduction

The small GTP binding proteins of the Ras superfamily (Ras, Arf, Rab, Rho, and Ran) are characterized by a low molecular weight (20–25 kDa), distinct structural motifs, and the ability to bind guanine nucleotides. Small GTPases function as regulators in virtually all cellular processes including signal transduction, cell division and growth, vesicular membrane traffic, cytoskeleton dynamics and cell motility [1]–[3].

Ras GTPases are the founding members of the family and are most noted for their critical role in cellular transformation and association with human cancers. [4]–[9] Arf family members assemble vesicle coat proteins and recruit lipid modifying enzymes and adaptor molecules to sculpt membranes and promote vesicle budding while, Rabs provide specificity and directionality by facilitating the transport and tethering of vesicles with target membranes. [10], [11] Rho GTPases are primarily associated with cell motility and cytoskeleton rearrangements and regulate the formation of stress fibers, focal adhesions, filipodia, and membrane ruffles. [12], [13] Rho GTPases also function in cell proliferation, transformation and differentiation. [14]–[16] Ran GTPase, which is the only member of this subfamily, plays a regulatory role in nucleocytoplasmic transport, mitotic spindle assembly, cell cycle progression, and the assembly of the nuclear envelope. [17]–[20] Other small GTPase-like proteins such as the RGK subfamily (Rad, Rem, Gem1, and Gem2) regulate voltage-gated calcium channels [21], [22].

Each of the small GTPase subfamilies has distinct functional niches. However, overlap clearly exists in their signaling routes. This is made apparent by the discovery of cascades and feedback loops that support a model in which the small GTPases communicate in a coordinated and cooperative manner. [23]–[25] This model also forecasts the presence of special signaling junctions where crosstalk takes place.

It is widely accepted that the bridging of the small GTPase pathways occurs in part through effector proteins such as guanine nucleotide exchange factors (GEFs), GTPase activating proteins (GAPs), scaffolds and membrane tethers as well as other molecules that interact with multiple GTPase family members, including the small GTPases themselves. [26]–[30] Presently, the small GTPase cross-talk phenomenon is not well understood due to insufficient information concerning the molecular mechanisms underlying the cellular events that mediate small GTPase communication as well as a lack of knowledge about the proteins that help to connect the small GTPase signaling pathways.

Direct and indirect interactions involving small GTPases and their regulatory/signaling proteins have been identified and validated through diverse methods that assess protein-protein interactions. The data derived from these interaction studies can be used to construct large scale graphs that present the overall architecture of cellular systems as well as the underlying interactions [31], [32].

To provide insight into the overall connectivity and topology of the small GTPase signaling pathways and to identify key players, a collective interaction network of the Ras, Arf, Ran, Rab, and Rho subfamilies was constructed based on experimental data supporting protein-protein interactions. The network is comprised of human proteins only and is a static/non-dynamical representation.

The results for the small GTPases network indicate a scale free model in which a few of the GTPases dominate the connectivity and hold the network together. Rac1, Cdc42, RhoA, and HRas are the hubs in the network. Other highly connected GTPases and non-GTPase proteins are also identified and described in this study, as well as the emergence of potential signaling avenues and higher order protein complexes.

## Methods

### Database

Interaction data for each of the individual small GTPases was obtained using the STRING database (Version 9.0; http://string-db.org/). The database was searched using the protein names for 139 unique small GTPases specifying isoforms from the Rho, Ras, Rab, Ran, Arf and RGK subfamilies (Spreadsheet S1). The data was hand curated with the search parameters specified exclusively for experimentally validated protein-protein interactions for human small GTPases.

In the case of experimental evidence based on known associations, STRING extracts information from a number of sources such as the Protein Data Bank (PDB), Molecular Interaction Database - European Bioinformatics Institute (IntAct-EBI), European Molecular Biology Laboratory (EMBL), Molecular Interaction Database (MINT), Biomolecular Interaction Network Database (BIND), Biological General Repository for Interacting Datasets (BIOGRID), and the Database of Interacting Proteins (DIP). The experimental methods for identifying interactions are diverse and include affinity capture-Western, affinity capture-mass spec, co-immunoprecipitation, FRET, co-purification, two-hybrid methods, complex reconstitution, and co-crystal structure. Associations are not limited to direct physical interaction between proteins and may also be linked through genetic interaction. Only GTPases that have one or more experimentally verified interactions were used to create the graph. To validate the curation approach used to construct the network, a manual inspection for each of the citations provided by the STRING database in support of the interactions for Rac1, RhoA, HRas, Ran, Rab5A, and Arf1 was performed. The protein expression levels and tissue/organ distribution have not been determined in this study.

### Network Analysis

The small GTPase interaction network was constructed based on data reported from methods that measure immediate physical interaction between protein pairs and data reported from methods that indicate both direct and indirect interactions such as co-immunoprecipitation, yeast two-hybrid, and tandem affinity purification, which may measure physical interactions among groups of proteins without taking pair wise interactions into account. These data were used to build individual networks for each of the small GTPase subfamilies with Medusa (Version 3.0; http://coot.embl.de/medusa/), a graph visualization program that interfaces with the STRING database. Subsequently, the individual networks were merged and analyzed with Cytoscape (Version 2.8.2; http://www.cytoscape.org/), a network visualization and analysis platform that supports a wide variety of plug-ins relative to network analysis and manipulation. [33] The complete small GTPases network was constructed using the graph union operation. Duplicated edges and self-loops resulting from reciprocal interaction detection and the graph merging procedure were removed prior to the analysis. The network was treated as undirected throughout the study, meaning that there were no distinctions implied between the vertices. Network Analyzer was used to calculate the basic network metrics such as the number of nodes and edges, degree distribution, degree exponent, path length, and clustering coefficient. Hubs and bottlenecks were identified with Cytohubba (Version 1.4; http://hub.iis.sinica.edu.tw/cytoHubba/).

Clusters were found with Molecular Complex Detection (MCODE) (Version 1.2; http://baderlab.org/Software/MCODE) using the haircut option which identifies nodes that have limited connectivity at the cluster periphery. [34] A value of 2.0 was used for the degree cutoff, representing the minimum number of edges for a node to be scored. The node score cutoff which controls how new nodes are added to the cluster was set at 0.2, which means that the score of the new node must be at least 80% that of the cluster’s seed node score. The K-Core, value which is used to filter out clusters lacking a maximally interconnected core, was specified for 3 edges.

To test the significance of global and local clustering, randomized graphs were generated with the Cytoscape Random Network plug-in (Version 1.5; http://sites.google.com/site/randomnetworkplugin/). To validate the clustering coefficient, a random network was generated from a degree preserving random shuffle of the real graph. In this algorithm, edges/connections are shuffled but the in-and-out degree of a node remains constant. Edges *(u,v)* and *(s,t)* were arbitrarily selected from the network with the constraints that u ≠ v ≠ s ≠ t and that (u,t) and (s,v) do not already exist in the network. Edges (u,v) and (s,t) were removed and edges (u,t) and (s,v) were inserted into the network. The clustering coefficient obtained for the random network represents an average of 100 randomizations. For validation of the clusters/motifs identified in the real network, MCODE was used to search for clusters in a randomized graph that was derived from a degree preserving random shuffle of the real graph with the same algorithm as described above for the validation of the clustering coefficient.

The Database for Annotation and Integrated Discovery (DAVID) (http://david.abcc.ncifcrf.gov/) together with its partner databases INTERPRO for domain prediction, and the Kyoto Encyclopedia of Genes (KEGG) for pathway mapping, were used to better organize and extract information about the proteins identified in the GTPase network. The complete gene list was submitted to DAVID under the functional annotation option specifying *Homo sapiens* as the species. Seven hundred and twenty-five list entries were annotated and assigned to DAVID categories. For the purpose of this study, the “Protein Domains” and “Pathways” categories were further explored for the assignment of known domains/motifs and mapping to established information flow diagrams. The initial results were filtered using the DAVID options feature to adjust the EASE score/P-Value limit to 0.05.

### Statistical Analysis

Data were analyzed with Josses’ In-Silico Online (http://in-silico.net/statistics) statistical analysis package. Statistical differences were assessed using the one sample Z test. A P value of <0.05 was considered statistically significant.

## Results

### Database

**Figure 1:**
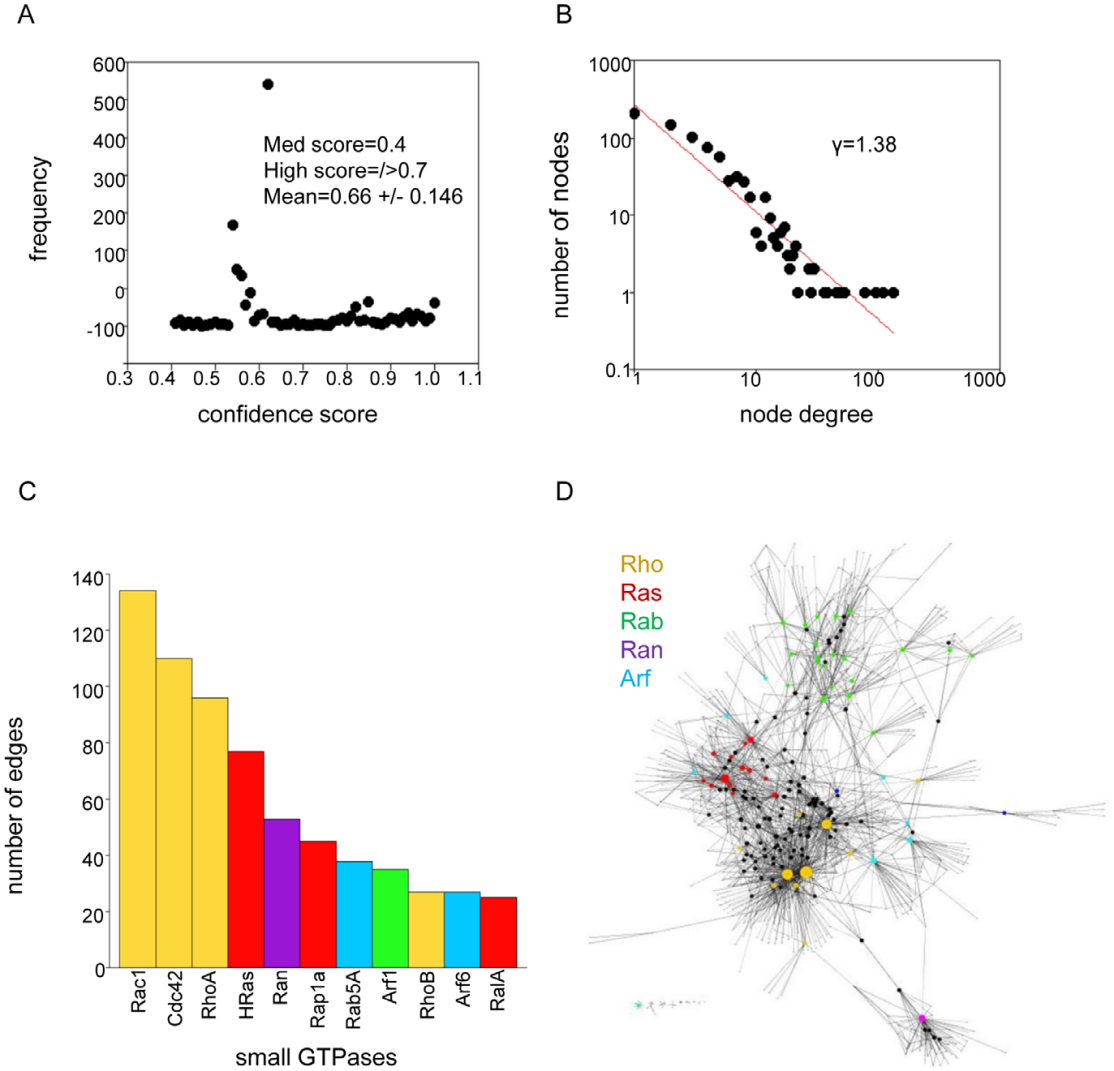
(A) Confidence score distribution. Plot of the frequency of occurrence vs. the confidence scores for the interaction data used to construct the network. (B) **Node degree distribution.** Plot of the log10 number of nodes with a given connection vs. the log10 node degrees in the network. The relationship between node number and node degree is described by the regression equation Y = 0,3354 X^−1.38^ (R^2^ = 86.0%). (C) **Bar plot of the small GTPases that have the greatest number of interactions.** Interaction number is labeled above each of the columns. Columns are colored to correspond to the small GTPase subfamilies. Yellow: Rho, Red: Ras, Green: Rab, Purple: Ran, Cyan: Arf. (D) **The small GTPases protein interaction network.** The graph is shown as a Biolayout (Enright 2001). Color coding for the Small GTPase subfamilies is as described in panel 1C. Non GTPase nodes are colored black. Proteins/nodes are represented as circles and are sized according to the number of connections.

The Search Tool for the Retrieval of Interacting Genes (STRING) database was searched for experimentally validated interactions of 141 unique small GTPases. The STRING database uses confidence scoring giving an estimate of how likely an association is to occur. The score is computed with reference to a benchmark set of known interactions from KEGG. Higher confidence scores are based on frequency of occurrence and reciprocal detection. [35], [36] By these criteria, a medium confidence threshold of 0.4 was specified in this study so as not to exclude interactions and to minimize ambiguity in the dataset. The frequency distribution for all confidence levels is shown in Fig. 1A. Two thirds of all interactions have a medium confidence score within the range of 0.4–0.7. The remaining one third of all interactions have a high confidence score in the range of 0.7–1.0. The average confidence score for all of the interactions is 0.66+/−0.146.

Experimentally verified interactions involving at least one other protein was reported for 98 of the GTPases. Interaction data for the Arf family, which included Arl and Sar proteins was the most incomplete. Only 13 out of 26 of the Arf family included in the search were involved in at least one experimentally verified interaction. This is mainly due to a lack of information concerning the Arl proteins. Experimentally verified interactions were reported for 8 out of 19 Arls. In comparison, 20 out of 22 of the Rho family GTPases, 19 of the 29 Ras family members, and 42 of the 59 Rabs were involved in at least one interaction. Ran and the RGK GTPases were also included in this study but are not taken into account individually because of the small number of members in these subfamilies (1 Ran and 4 RGK GTPases).

To validate the curation approach used to construct the GTPase interaction network, each of the citations provided by the STRING database in support of the interactions for Rac1, RhoA, HRas, Ran, Rab5A, and Arf1 were inspected. These six GTPases together with their interacting partners collectively account for 55% of all the proteins and 22% of all the interactions in the network and can therefore be considered a substantial sampling of the population.

From the re-curated literature, 71% of the interactions are supported by one publication. Sixty-six percent of this group is supported by one detection method whereas, 34% are supported by multiple detection methods. The remaining 29% of the interactions are supported by multiple publications. Of these, 11% of the interactions are supported by a single detection method and 89% are supported by multiple methods (Table S1.).

There were 12 inaccurate citations in total supporting 5 HRas interactions (ARHGEF1, RHEB, RAP1B, HGF, RALA), 2 Rac1 interactions (FLNA, CDC42), 2 RhoA interactions (ARHGEF12, PLEKHG2), 2 Rab5A interactions (RAB7A, RIT2), and 1 Arf1 interaction (PIP5K1A). Five of these interactions were supported by other references (Rac1/FLNA, HRas/RALA, HRas/HGF, RhoA/ARHGEF12, and Arf1/PIP5K1A). Published evidence could not be found in support of 7 interactions (Rac1/CDC42, HRas/RHEB, HRas/RAP1B, HRas/ARHGEF1, RhoA/PLEKHG2, Rab5A/RAB7A, Rab5A/RIT2. In summary, of the 429 protein-protein interactions considered in this validation step, 98.4% were confirmed positive.

This careful inspection of the data reveals a 97.1% accuracy and a corresponding 2.9% error rate. The interactions that could not be validated by the supporting literature were due to either semantic mix-ups or were just simply incorrect. Most of these inconsistencies (42%) occurred in the literature for the HRas interactions. The 7 interactions that could not be validated were excluded from the network.

This validation step was undertaken to determine the authenticity of the references and to assess whether the published data correlate with the detection methods reported by the STRING database. Based on the results, it would appear that the STRING database is a reliable and valuable source of curated protein-protein interaction data.

### Architecture of the Small GTPase Interaction Network

For biological systems, a network analysis can reveal the connectivity underlying cellular interactions by identifying unknown proteins in an established pathway or multi-subunit complex. Networks are typically evaluated on two levels: the topology which describes the architecture of the graph as well as the interactions within. Due to the increased application of network analysis to study biological systems, quantifiable measures to characterize and compare biological networks are in place. Some of the basic network metrics are the node degree distribution, the degree exponent, path length, and the clustering coefficient [32], [37].

The most telling parameter in the evaluation of a network is the node degree distribution. This metric is used to distinguish between different types of networks such as random versus scale-free. [37] In the random network model, most nodes have a similar number of connections and will follow a Poisson distribution whereas, in the scale free model a small number of nodes have many links while the rest of the nodes engage in relatively few interactions. [38]–[40] Scale free networks have a non-uniform distribution and occur most often in cellular systems [32].

The term scale-free indicates the absence of a particular node in the network that can be used to characterize the other nodes. The node degree distribution of a network, P(k), gives the probability that a node has exactly k links. P(k) is obtained by counting the number of nodes N(k) with k = 1,2… links and dividing by the total number of nodes N. By definition, in the scale free model the number of nodes with k links follows a power law distribution; [38], [41].

In this equation γ is the degree exponent.
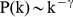
(1)


The small GTPase network described here is comprised of 778 nodes (proteins) and 1943 degrees (interactions); (Fig. 1D, Table S2 and Graph S1). The degree distribution approximates a power law and indicates a scale-free topology (Fig. 1B). The majority of the proteins/nodes in the small GTPases network have less than 15 interactions. Four nodes have a large number of connections: Rac1 (134), Cdc42 (110), RhoA (96), and HRas (77), respectively. These four GTPases account for 21.5% of all the connections in the network. A gap occurs in the distribution and four additional nodes emerge that also have a substantial number of interactions: Ran (53), Rap1A (45), ARF1 (38), and RAB5A (35), as compared to the other proteins/nodes in the network. These account for 8.8% of all interactions. Together these eight highly connected GTPases account for 30.3% of all links in the small GTPase network (Fig. 1C).

In the context of a network the term “hub” is used to describe a node with a very large number of connections. Hubs have a central role in the structure and organization of a network and may be more important biologically than lesser connected nodes. [42] The hub designation itself, however, is somewhat arbitrary. For clarity, hubs are defined here as proteins that are in the top ∼20% of the degree distribution or in other words, proteins that have the ∼20% highest number of neighbors. Based on this criteria, Rac1, Cdc42, RhoA, and HRas are the hubs identified in the small GTPases network. These GTPases have multiple cellular roles that involve the regulation of cytoskeleton dynamics and cell growth processes and are strongly associated with human cancers [12], [13].

Other highly connected proteins identified in the analysis include the GTPases, Ran, Rap1A RalA, Arf1, Arf6, Rab5A, and RhoB. Rap1A and RalA are Ras subfamily members and are associated with endothelial cell adhesion and tumor genesis, respectively. [43], [44] Arf1 and Arf6 regulate vesicular membrane traffic at the Golgi, plasma membrane, and endocytic pathway. [45], [46] Rab5A, is a key regulator of endocytic processes and RhoB is associated with apoptotic signaling. [47], [48] The non-GTPase proteins are V-Raf-1 Murine Leukemia Viral Oncogene Homolog 1 (Raf-1), which is serine/threonine kinase involved in transferring mitogenic signals from the cell membrane to the nucleus; Phosphatidylinositol 3-kinase regulatory subunit 1 (PIK3RI), an adaptor that binds to activated/phosphorylated protein tyrosine kinases; and Rho GTPase dissociation inhibitor (ARHGDI). Some, if not all of these proteins may eventually emerge as hubs in the evolution of the small GTPase network when the interaction profiles are more complete.

### Topology and Organization

Visual inspection of the small GTPases network (Fig. 1D) shows that the Rho (yellow) and Ras (red) GTPases generally occur in the densest regions of the graph. Members of the Arf family (cyan) border the central area but are situated in less dense regions. Also, several of the Arfs (cyan) branch off from the main portion of the graph. The Rab GTPases (green), despite being the largest of the small GTPases subfamilies, are in the sparsest region of the graph and have limited connectivity to each other and to the rest of the network as well. In contrast, Ran GTPase (purple) has many interactions but is connected to the network by a relatively small number of links.

There are also several GTPases that are not connected to the main network at all. These are Arl4a, Arl5A, Arl6, Arl11, RhoBTB2, Miro1, Miro2, Rab24, RasD1, NKIRas1, and NKIRas2. The lack of connectivity for these proteins may be due to the absence of interaction data. Alternatively, they may represent future branch points or sub graphs like that of Ran. Several of these GTPases are rather unique. For example, Arl5A and Rab24 have nuclear/nucleolar localization profiles, while Rho family members Miro1 and Miro are involved in mitochondrial transport. NFKB inhibitor interacting Ras-like 1 (NKIRas1/II) is also considered an atypical small GTPase because it acts as a potent regulator of NF-kappa-B signaling [45], [49]–[53].

Networks grow and evolve. The degree exponent (γ) from equation 1 provides insight into some of the basic properties concerning hubs and the growth and evolution of scale-free networks. It has been demonstrated that both biological and non-biological networks exhibit power law graphs but the degree exponent can fall into different ranges. [54] For non-biological networks such as the internet and social networks, the degree exponent may vary between 2 and 4 whereas for biological networks the degree exponent typically ranges between 1 and 2 [37], [55].

With respect to hubs, the effect of the degree exponent in scale free graphs is as follows. For γ greater than 3, the hubs may not be relevant and the graph behaves in a random-like manner. For cases in which γ is between 2 and 3 there is a hierarchy of hubs, with the most connected hub being in contact with a small fraction of all nodes, and in cases where the degree exponent is equal to 2, the largest hub is in contact with a large fraction of all nodes [37], [55].

In the situation where the degree exponent is less than 2, the graph still behaves in a scale-free manner but indicates that perhaps the growth and evolution of the protein network follows a partial duplication model rather than the preferential attachment scheme. [54] In the small GTPases network, the degree exponent for the node distribution curve is equal to 1.38 and falls within the range of that reported for other biological networks (Fig. 1B). There is also a hierarchy of hubs which in some instances are connected to each other as well as lesser connected nodes within the network (Fig. 1D).

### Navigability

**Figure 2:**
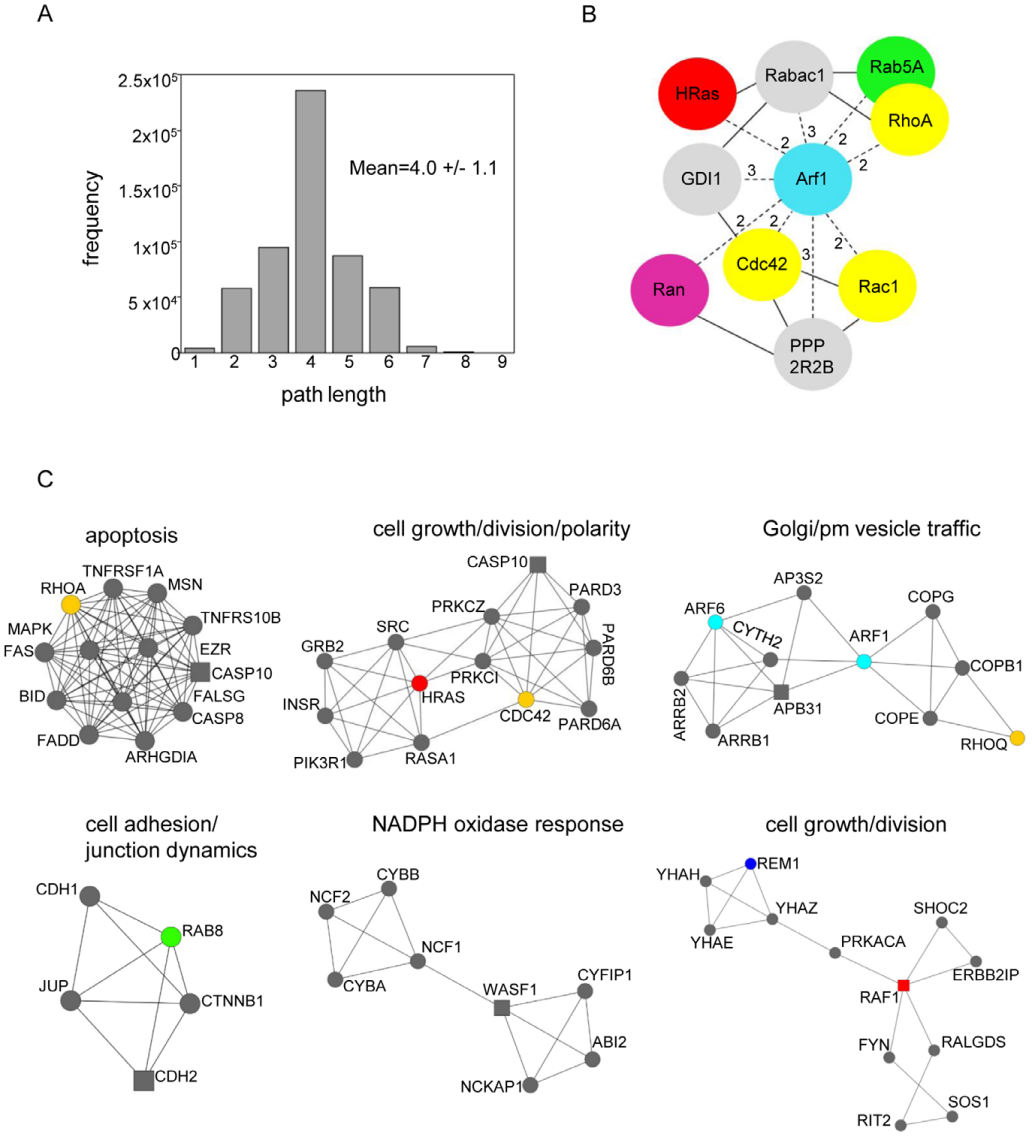
(A) Path length distribution. Frequency distribution of path lengths for all interactions in the network. (B) **Bottlenecks.** Cartoon representation of the top 10 bottleneck proteins. Solid lines signify 1^st^ (immediate) neighbor interactions and dotted lines represent 2^nd^ and 3^rd^ (nearest) neighbor interactions respectively, as labeled in the figure. (C) **Network motifs.** Representative clusters (1, 2, 3, 4, 5, and 10) with biological themes that were identified in the network. The highest scoring (seed) nodes in the cluster are shown as squares.

Path length describes the number of steps/edges along the shortest paths for all possible pairs of nodes in the network and is a measure of the efficiency of information transfer in the network as well as the overall navigability. [56] The path length distribution for the small GTPases network is shown in Fig. 2A. The mean path length is 4.0. A smaller number of path lengths fall into the extreme upper categories (path lengths 8 and 9) as compared to the lower extremes (path lengths 1 and 2), indicating that most of the proteins within the graph can be linked to every other protein by a small number of paths. Generally, short paths are considered more desirable because they facilitate rapid transfer of information at less cost. [37], [40], [56] One drawback however, is that they may be highly vulnerable to local disturbances which can travel throughout the network quickly.

### Bottlenecks

Another intriguing aspect of a network analysis is the potential identification of proteins that act as bridges by connecting different groups of proteins and/or different parts of the network to one another. [57] These nodes/proteins are analogous to heavily used intersections and are referred to as “bottlenecks” and, like hubs, have a special role. Bottlenecks are identified by determining the number of non-redundant shortest paths going through a particular node. [42] Any node in the network can be a bottleneck, but hub-bottlenecks appear to be even more critical because their removal is able to disrupt the network with greater efficiency.

The small GTPases network was analyzed for bottlenecks that bridge signaling pathways. The ten top scoring bottleneck proteins were identified in the network (Fig. 2B). Seven of these are GTPases. The highest scoring bottlenecks are Cdc42, RhoA, and Rac1, which are also the highest ranked hubs, except that in this case the ranking order is changed (hub order: Rac1, Cdc42, RhoA). The other hub, HRas, was also identified as a bottleneck but it ranked lower than the Arf1 GTPase. Ran and Rab5A are the remaining GTPases identified and rank within the top ten bottlenecks. The non-GTPase proteins are Protein Phosphatase 2 beta subunit, Prenylated Rab acceptor protein 1, and Rab GDP Dissociation Inhibitor (GDI1) alpha. Each of the small GTPase families is represented by at least one member. The Rho family is represented by 3. The bottlenecks are all interconnected by either 1, 2, or 3 path lengths. Interestingly, all of the bottlenecks connect to Arf1 which is shown at the center of the sub graph (Fig. 2B).

### Proteins Shared among Multiple Small GTPase Networks

The individual networks of each small GTPase subfamily were cross checked for mutually interacting proteins in order to gain an understanding of how their individual signaling routes may intersect. Eighty-four proteins were identified in total (Table S3). There were no proteins identified that occurred in all of the small GTPase subfamily networks. One protein, cGMP 3′,5′-cyclic phosphodiesterase subunit delta, was present in 4 of the small GTPase subfamily networks (Arf, Rho, Ras, and Rab). Seven proteins were present in 3 subfamily networks: Caveolin 1 (Arf, Rab, Rho); Melatonin receptor 1A and Prenylated Rab acceptor protein 1 (Rab, Ras, Rho); and Insulin receptor (INSR), Phospholipase D1, Protein phosphatase 2, and Protein kinase C iota (Arf, Ras, and Rho). The remaining seventy-six proteins were shared between two small GTPase networks. Most of the possible combinations were represented, except for the Ran/Ras and the Ran/Rab networks. Thirty-six mutually occurring proteins were shared by the Rho/Ras networks. For the other combinations of shared proteins, 9 occurred in both the Rho/Rab and Ras/Rab networks respectively, 8 in the Rab/Arf networks, 6 in the Rho/Arf networks, 5 in the Ras/Arf networks, 2 in the Ran/Rho networks and 1 in the Ran/Arf networks. The first/nearest neighbor interactions for proteins that are present in at least 3 GTPase subfamily networks are listed in Table S4.

To gain additional functional interpretation and organization of the proteins identified in the GTPase network. The Database for Annotation and Integrated Discovery (DAVID) together with its partner databases INTERPRO for domain prediction, and KEGG for pathway mapping, were explored. From the 778 proteins comprising the network, 706 (97.5%) were categorized according to 134 INTERPRO/GO domains at the superfamily, family, and subfamily levels (Spreadsheet S2). Besides the GTPase domain, Protein kinase, Pleckstrin homology and assorted GEF, GAP, and GTPase binding domains for the different GTPase subfamilies were most strongly enriched (Fisher’s exact test, P value ≤0.05). Signaling domains such as SH2, SH3, and C2 calcium-dependent membrane targeting domains were also highly represented.

The KEGG pathway database is considered the gold standard for mapping datasets to a diverse collection of information flow diagrams that incorporate current knowledge of molecular interactions and reaction networks. To this end, the KEGG pathway mapping feature was used to place the GTPase network interactions in a broader biological and physiological context and to help guide future studies aimed at identifying novel interacting partners in a more directed manner.

Three hundred and forty-six (44.3%) of the genes from the GTPase network mapped to 57 distinct KEGG pathways (Fisher’s exact test, P value ≤0.05.). The resulting pathways are representative of diverse cellular systems, processes, and diseases (Spreadsheet S3). Many are cancer related and most often involve either Ras and/or Rho family members. Surprisingly, there are only 2 KEGG pathways that include members of the Rab family (hsa04530:Tight junction, hsa04144:Endocytosis) and there are none that contain Ran or Arf family GTPases. The Endocytosis (hsa04144) map however, does contain ARFGAP1, which is a GTPase activating protein for Arf GTPases. Visual representations of the maps can be accessed by searching the KEGG database with the corresponding “hsa” reference number provided in Spreadsheet S3.

### Clustering and Modularity within the Small GTPases Network

The clustering coefficient is a network parameter that measures the degree to which nodes/proteins in a graph tend to cluster together. [40] This is important because it can provide insight into the overall organization of the relationships within a network and may also indicate the presence of functional modules which, in the case of protein networks, can represent higher order complexes or signaling pathways [34], [37], [58], [59].

The clustering coefficient quantifies the number of connected pairs between a node and its neighbors and can be measured both globally and locally. The global assessment provides an average of the clustering coefficients for all the nodes in the entire network, while the local clustering coefficient represents the embeddedness of single nodes [40], [60].

Generally, the clustering coefficient is a ratio *N*/*M*, where *N* is the number of edges between the neighbors of *n*, and *M* is the maximum number of edges that could possibly exist between the neighbors of *n*. [37], [40], [60] The clustering coefficient of a node is always a number between 0 and 1. The network clustering coefficient is the average of the clustering coefficients for all nodes in the network. Here, nodes with less than two neighbors are assumed to have a clustering coefficient of 0. For the small GTPases graph the global clustering coefficient is 0.33, whereas the clustering coefficient for a randomized network generated on the same vertice set is 0.066+/−0.006. (*z* = −477.81, p<0.0001) indicating that the clustering observed in the small GTPases network did not occur by chance alone.

Local clustering is typically detected with clustering algorithms that consider highly inter-connected dense regions within a network. [34] The term “cliquishness” is often used to describe the behavior of these groupings. Ten clusters were identified in the small GTPases network and are rank ordered according to their density (inter-connectivity) and size (number of proteins) (Table 1, Fig. 2C and Fig. S1). Out of 778 total proteins in the network, 77 (10%) of these are located within clusters. The functional descriptors are based on the STRING, BioGrid, and Gene Ontology terminology.

**Table 1:** Clusters in the small GTPases network.

Cluster theme	Score	Nodes	Edges	Gene symbol
Apoptosis	6.0	13	78	ARHGDIA, BID, CASP10, CASP8, EZR, FADD, FAS, FASLG, MAPK8, MSN, RHOA, TNFRSF10B, TNFRSF1A
Cell growth, division and polarity	3.1	13	40	CDC42, GRB2, HRAS, INSR, PARD3, PARD6A, PARD6B, PARD6G, PIK3R1, PRKCI, PRKCZ, RASA1, SRC
Golgi/pm vesicle traffic	2.0	11	22	AP3B1, AP3S2, ARF1, ARF6, ARRB1, ARRB2, COPB1, COPE, COPG, CYTH2, RHOQ
Cell adhesion and junction dynamics	1.8	5	9	CDH1, CDH2, CTNNB1, JUP, RAB8B
NADPH oxidase response	1.6	8	13	ABI2, CYBA, CYBB, CYFIP1, NCF1, NCF2, NCKAP1, WASF1
Transcription regulation	1.5	4	6	SIN3A, SMARCA2, SMARCB1, TAF2E
Receptor- mediated endocytosis	1.5	4	6	RAB11A, RAB11FIP2, RAB11FIP4, RAB11FIP5
Gene silencing	1.5	4	6	ARL5A, CBX1, CBX2, CBX3, CBX5
Growth factor signaling	1.5	4	6	RALB, RALBP1, REPS1, REPS2
Cell growth and division	1.3	12	16	ERBB2IP, FYN, PRKACA, RAF1, RALGDS, REM1, RIT2, SHOC2, SOS1, YWHAE, YWHAH, YWHAZ
Control 1	2.0	16	32	PAK1, PLCG1, SRC, CHM, RHOB, RHOA, MRAS, RRAS, RAB5A, RAB4A, KRAS, RAN, RAF1, RAB9A, ARAF, ARF1
Control 2	1.6	14	23	CASP10, TNFRSF1A, ARHGAP1, RAB11A, EEA1, NCK1, COPB1, NCF2, RAP1A, RAB2A, RAB11FIP5, CASP8, RALA, HRAS

Score is defined as the product of the cluster density and the number of proteins in the complex sub graph (DC x |V|). Larger and denser complexes are ranked higher.

### Cluster 1 Apoptosis

The overall functional theme of cluster 1 is apoptosis (Fig. 2C). This is the highest scoring cluster and includes RhoA which was identified as a hub in the small GTPases network. RhoA has a regulatory role in actin cytoskeleton dynamics and links plasma membrane receptor signaling to the assembly of stress fibers. RhoA also plays a role in cell adhesion. [12], [13], [16] Most of the other proteins in this cluster have established roles in either the intrinsic or extrinsic apoptotic pathways. BH3 interacting domain death agonist (BID) is a pro-apoptotic member of the B-cell lymphoma 2 (Bcl-2) family or apoptotic regulator proteins and interacts with Bcl-2 associated X protein (BAX), another Bcl-2 family member. [61], [62] The Bid-Bax interaction facilitates the insertion of Bax into the outer mitochondrial membrane and ultimately the release of cytochrome c. [63], [64] Mitogen-activated protein kinase 8 (MAPK8) is also believed to be related to the cytochrome c mediated death pathway. [65] MAPK8 is required for Tumor necrosis factor (TNF) alpha induced apoptosis and is activated in response to environmental stress such as UV radiation. [15] The other apoptotic-related proteins in this cluster are implicated in the TNF and TNF associated factor 1 (Fas) death pathways. [66] These include the TNF receptor superfamily member (Tnfrs) 1A, Tnfrs 10B, and Fas receptors, the Fas associated death domain (FADD) adaptor, and the Fas ligand (FASLG). Fas and FADD are members of the death induced signaling complex (DISC). [67], [68] The Fas-FADD complex recruits and activates Caspase-8 and Caspase-10 which are also present in this cluster. Cleavage of Caspase-8 by DISC initiates the subsequent cascade of caspases mediating apoptosis [69], [70].

In some cell types the Fas-DISC death route initiates a feedback loop that causes an increased release of pro-apoptotic factors from mitochondria. Caspase-8 links the extrinsic and intrinsic paths. [71], [72] The remaining proteins in cluster 1 are Ezrin and Moesin which are implicated in the early stages of apoptosis, where they act as linkers by connecting cytoskeleton structures to the plasma membrane. [73] Both of these proteins associate with the Fas receptor and are substrates of the Rho effector, Rho-associated coiled-coil containing protein kinase 1 (ROCK1). ROCK1 is rendered constitutively active after cleavage by caspases during apoptosis and is responsible for bleb formation in apoptotic cells. [74], [75] The only protein in the cluster that is not directly associated with apoptosis is ARHGDIA which delivers Rho GTPases to target membranes [76]–[78].

### Cluster 2 Cell Division/Cell Growth/Cell Polarity

The majority of the proteins in cluster 2 are strongly associated with cell growth, division and polarity (Fig. 2C). This cluster includes the small GTPases, HRas and Cdc42. HRas is involved in various signal transduction pathways and has a well defined role in regulating cell division in response to growth factor stimulation. Cdc42 is a member of the Rho family and has an important role in the control of cell growth by mediating the establishment of cell polarity. Cdc42 together with Rac1, another Rho family member, are also involved in Ras-mediated oncogenic transformation. [1], [79] Growth factor receptor-bound protein 2 (GRB2) is an adapter protein that links the INSR and the Ras signaling pathway. GRB2 interacts with the INSR and the Ras effector/activator Son of Sevenless (SOS). Ras acts downstream of INSR signaling and initiates either the Raf/MEK/ERK path or an alternate route involving the proteins partitioning defective (PARD). [80] PARDs are self-associating adaptors that are implicated in asymmetric cell division and cell polarization processes. PARD family members also associate with atypical Protein Kinase C (PKC) proteins, and Cdc42. [81] In epithelial cells the Par/PKC/Cdc42 interaction is involved in the formation of tight junctions. [82], [83] In the cluster identified in the small GTPases network, atypical PKC (iota and zeta) are present.

The other proteins in cluster 2 that are involved in cell growth/proliferation are Phosphatidylinositol 3-kinase (PI3K) which also interacts with the INSR and the Src proto-oncogene protein tyrosine kinase. [84] p120-RasGAP is a negative regulator of the Ras/MAPK signaling pathway, which transmits signals from outside the cell to the nucleus. [85] The Ras/Mitogen activated protein kinase (MAPK) signaling pathway is involved in the growth, proliferation, differentiation, and motility of cells [86].

### Cluster 3 Golgi/Plasma Membrane (Pm) Vesicle Traffic

Cluster 3 (Fig. 2C) contains the small GTPases, Arf1, Arf6, and RhoQ as well as adapter proteins, components of the coatomer complex, and proteins that interact with G-protein coupled receptors (GPCRs). Arf1 mediates vesicle budding from donor membranes by promoting coat protein assembly through the recruitment of Adaptor protein 1 (AP-1) and coatomers. Coatomer is a multi-subunit protein complex that reversibly associates with Golgi non-clathrin-coated vesicles and mediates ER to Golgi protein transport. [45] Adaptor-related protein complex 3, beta 1, and sigma 2 are subunits of the Adapter protein 3 (AP-3) which plays a role in protein sorting in the late-Golgi/trans-Golgi network and/or endosomes. [87], [88] RhoQ regulates actin polymerization on membrane transport vesicles and interacts directly with COPI coat proteins. [89] Arf6 regulates endocytic membrane traffic and actin remodeling at the plasma membrane and Golgi. Arf6 also interacts with Beta arrestins 1/2 which are adaptors that link G protein coupled receptors (GPCRs) to endocytic proteins like Arf6. [90], [91] Cytohesin 2 is a guanine-nucleotide exchange for Arf1, Arf3 and Arf6 [92].

### Cluster 4 Cell Adhesion/Junction Dynamics

Cluster 4 (Fig. 2C) is comprised of proteins involved in cell attachment and cell junction processes. The small GTPase Rab8B is associated with polarized membrane transport and tight junction dynamics. [93], [94] Rab8 interacts with other junction proteins such as cadherin and catenin. Cadherin type 1 and 2 are calcium-dependent cell adhesion proteins. Cadherin-associated protein is involved in the regulation of cell adhesion. [95] The other protein in this cluster, Junction plakoglobin, is a membrane associated junctional plaque protein that associates with cadherins and influences the arrangement and function of cells within tissue [96].

### Cluster 5 Nicotinamide Adenine Dinucleotide Phosphate Oxidase Response

Cluster 5 (Fig. 2C) does not contain any of the small GTPase family members but an association with Rac1/2 is implied, based on the literature and the other proteins present in the cluster. [97] This cluster is comprised of proteins involved in the production of superoxide in phagocytes such as the nicotinamide adenine dinucleotide phosphate (NADPH) oxidase subunits, Neutrophil cytosolic factors 1/2 (NCF 1/2, Abl interactor 2, and Cytochrome B-245 alpha/beta, which are part of the microbial oxidase response of the innate immune system. [98] Rac1 is also a component of the NADPH oxidase complex [99].

The other proteins in the cluster are components of the WAVE complex. NCK associated protein 1, Wiskott-Aldrich syndrome protein family member 1, and Cytoplasmic FMR1 interacting protein 1 are all related to Rac dependent actin remodeling and the formation of membrane ruffles/lamellipodia [100], [101].

### Cluster 6 Transcription Regulation

The proteins in cluster 6 (Fig. S1) are involved in transcription regulation and are associated with the network through Ran GTPase. Transcription regulator homolog A acts as a transcriptional repressor, while SWI/SNF-related matrix-associated actin-dependent regulator of chromatin a2/b1 and TAF6 RNA polymerase 2 are part of the chromatin remodeling complex. [102] SNF/SWI is required for the activation of genes that are repressed by chromatin [103].

### Cluster 7 Receptor-mediated Endocytosis

Cluster 7 (Fig. S1) is concerned with receptor-mediated endocytosis and involves Rab11A which modulates endosomal trafficking at the plasma membrane and recycling endosomes. The Rab11 family interacting (Rab11FIP) proteins are effectors for both Rab and Arf GTPases and are involved in protein trafficking from the apical recycling endosome to the apical plasma membrane endosomes. [29], [104]–[106] The different forms of Rab11FIP 2,4,5 represent splice variants.

### Cluster 8 Gene Silencing

The overall theme of cluster 8 (Fig. S1) is gene silencing. Arl5A is a member of the Arf GTPase subfamily and is uniquely localized to the nuclear/nucleolar compartments. [107] The Chromobox homologs 1, 3, and 5 proteins are components of heterochromatin and are involved in epigenetic repression and gene silencing [108].

### Cluster 9 Growth Factor Signaling

Cluster 9 (Fig. S1) is associated with receptor mediated endocytosis and growth factor signaling. RalB is a member of the Ras GTPase sub-family and is involved in a variety of cellular processes including gene expression, cell migration, cell proliferation, oncogenic transformation, and membrane trafficking. [109] RalB binding protein 1 (RALBP1) is a RalB GTPase activating protein. RalBP1 associated Eps domain containing 1/2 (REPS1/2) is implicated in endocytosis and has a negative effect on receptor internalization and inhibits growth factor signaling [110].

### Cluster 10 Cell Growth and Division

Cluster 10 is the lowest scoring cluster in the group (Fig. 2C). It has a low density to interaction ratio (12/16) but in spite of this, the cluster still appears to have an overall theme in cell growth and division. The topology of the cluster has two distinct units. The first involves v-raf-1 murine leukemia viral oncogene homolog 1, Erbb2 interacting protein, and Shoc-2 suppressor of clear homolog which together form a closed triangle. Raf-1 is involved in the transduction of mitogenic signals from the cell membrane to the nucleus. [111] The erbb2 interacting protein is an adapter for the erbb2 receptor in epithelial cells. Erbb2 belongs to the EGF family of receptor tyrosine kinases. [112], [113] Shoc2 is an MRas effector and plays a role in the MAPK pathway where it is a negative regulator of Ras signaling. [114] The other proteins associated with this unit are connected to the triplet but form an open structure that does not have interconnectivity. Still the general theme, cell growth and cell division through Ras signaling, is retained. Ral guanine nucleotide dissociation stimulator is a GEF for RalA and RalB and is also an effector for Ras and Rap. [115], [116] Ras-like without CAAX2 (RIT2) is a Ras-like small GTPase and as the name implies, does not possess the CAAX membrane association motif. SOS1 is a Ras GEF and tyrosine protein kinase isoform (Fyn) is an src related oncogene that is implicated in cell growth. [117] Fyn also plays a role in the regulation of intracellular calcium levels [118].

The second subgroup of cluster 10 has high interconnectivity and is comprised of an RGK family GTPase, Rem1, promotes endothelial cell sprouting and actin cytoskeleton reorganization and is also implicated in angiogenesis and Ca(2+) signaling. [119] The other proteins are different subunits of the 3-monooxygenase/tryptophan 5-monooxygenase activation protein which belong to the 14-3-3 family. (YWHAE, YWHAH, and YWHAZ). The YWHAZ subunit is implicated in mitogenic signaling and the cell cycle through an interaction with phosphatases via Raf1 and cell division cycle 25. [120] The two subgroups of cluster 10 are linked through protein kinase A which is the alpha subunit of a cAMP-dependant protein kinase that has broad substrate specificity for a large number of proteins in the cytoplasm and the nucleus. [121], [122] Cluster 10 may represent a valid complex or a partial signaling pathway despite a low clustering score but at this stage, additional interaction data is needed for verification.

For validation of the clusters, the real GTPase network was randomized and analyzed for sub graphs using the same clustering algorithm and parameters as for the identification of clusters within the real network. Two clusters resulted from the randomization (Labeled control 1 and control 2 in Table 1. Not shown in figure.) Unlike the clusters identified in the real network, the clusters picked out of the random network cannot be ascribed a collective functional role and are nonsensical.

Nevertheless it is conceivable that proteins involved in similar cellular processes could arise from a cluster analysis of a randomized network by chance alone. Interestingly, the control clusters have scores of 2.0 and 1.6 respectively, which are in the same range as some of the lower scoring clusters identified in the real network that have seemingly authentic associations. The MCODE scoring scheme scores larger, more dense complexes, higher than smaller more sparse complexes. [34] The control clusters identified in the random network have a large number of proteins and their scores are attributed to this. Importantly, this indicates that the validity of clusters cannot rely solely on the scoring from the clustering algorithm but need to be further validated with additional information.

The clusters identified in the real graph most probably represent bona fide complexes and signaling pathways, but it is important to keep in mind that the analysis method described here is predictive. Even though clear functional themes emerge, the data should be considered with caution until these multiple interactions are experimentally verified.

## Discussion

This study describes the architectural and functional properties of the small GTPases network that was constructed based on experimentally validated data for protein-protein interactions. The information takes into account the current state of human protein interaction data involving small GTPases and represents all of the possible relations that may occur, irrespective of cell and tissue expression profiles, but does not consider whether or not the interactions are mutually exclusive/inclusive.

The small GTPases network incorporates protein-protein interaction data derived from primarily three kinds of investigations: (1) Studies concerned with identifying interacting partners for specific GTPases and their regulatory factors (GEFs, GAPs, GDIs, Lipid modifying enzymes etc). (2) Studies centered on a variety of other proteins and cellular processes unrelated to GTPase biology in which interactions with GTPases and/or their regulatory factors were identified serendipitously. (3) Large-scale, high-throughput investigations designed with the sole intention of identifying protein-protein interactions and having no motivation to fish out GTPase specific interacting partners. Publication/study bias could conceivably arise in the network based on data obtained from the first type of investigation. However, many of the interactions used in the present analysis come from the latter two types of studies which are unbiased. Further, it is conceivable that some of the interactions included in the network may be false positives. To minimize the occurrence of this, a medium confidence level was used as an initial filter for data collection. Subsequent analysis of the confidence level distribution was used as a guide to assess the overall strength of the data which was further supported by the results obtained from the data validation step which reveal a high level of accuracy.

The small GTPases network is primarily held together by Rho and Ras family members. The hubs, (Rac1, Cdc42, RhoA, and HRas) identified in the network are widely studied proteins due to their association with human cancers and core cellular processes. Rac1, Cdc42, and RhoA belong to the Rho GTPase family and have a special functional relationship. Each of these GTPases has a unique role in actin cytoskeleton dynamics and work sequentially and cooperatively to preserve the fidelity of cell migration processes. Rac1 controls the formation of lamellipodia and membrane ruffles. [12], [13] Cdc42 regulates filipodia formation and is involved in the establishment of cell polarity whereas RhoA controls the formation of stress fibers and focal adhesions. [16], [123] Besides cell motility, these proteins are involved in cell cycle progression and transcription regulation [49], [79], [124].

HRas is a multifunctional GTPase that is involved in cell proliferation, transformation, and apoptosis. Remarkably, impaired Ras signaling is related to 20–30% of all human cancers. [1] The Rho GTPases are also associated with cancer progression and invasiveness, and crosstalk and cooperativity between Rho and Ras GTPases is well established. [79] Each regulates a set of critical overlapping processes in mammalian cells such as gene expression, cell proliferation, and actin-based cell motility. [4], [125] The Rho and Ras pathways are bridged through a wide array of GEFs/GAPs and effectors such as kinases. [74], [126] There is also evidence for simultaneous activation of both GTPase subfamily members like Ras and Rac in response to upstream insulin and integrin receptor signaling [80].

The bimodal nature of the node degree distribution observed in the small GTPases network indicates that Arf1, Ran, Rab5A, and Rap1A may potentially emerge as hubs when the interaction data are more complete. These GTPases have a large number of connections as compared with the other nodes in the network but have ∼1/3 of the interactions of the Rho and Ras hubs This may be because Arf1, Ran, Rab5A, and Rap1A have not been studied as extensively as Rho and Ras, or it may be that the small GTPases network has at least a two tiered hub hierarchy. In addition to hub activity, Rac1, Cdc42, and RhoA, are also bottlenecks in the network along with the GTPases Arf1, Rab5A, and Ran. Proteins that have this special dual role as hubs and bottlenecks are considered more essential to a network than either hubs or bottleneck proteins alone, because they engage in a large number of interactions and are strategically positioned at branching points and/or signaling junctions. Hub bottleneck proteins are also appealing targets to uncouple signaling events for further study or as potential candidates for drug therapies [42], [57].

In addition to bottlenecks, the proteins that are shared between the individual small GTPase subfamily networks are also identified in this study. These proteins are an assortment of kinases, adaptors, receptors, lipid modifying proteins, and various types of effectors and regulatory proteins that are specific to most GTPases such as GEFs, GAPs, and GDIs. The proteins that occur between at least 3 small GTPase subfamily networks are potentially interesting for further investigation because they are linked to multiple GTPase subfamily members through immediate and nearest neighbor interactions. Targeting these proteins may also be instrumental in understanding how the small GTPases signaling routes connect.

The functional modules found in the small GTPase network underscore the diversity of signaling pathways mediated by these proteins. The major themes observed here are apoptosis (cluster 1), cell growth and division (clusters 2 and 9), vesicle traffic (clusters 3 and 7), and transcription regulation (cluster 6 and 8). Perhaps, over time, additional themes will be brought to light as well, such as the role of small GTPases in the NADPH oxidase response (cluster 5).

In this study, the parameters used for cluster identification were conservative, and it is therefore, likely that the associations described here represent the core components. The actual signaling modules are probably more intricate and involve many more proteins. As the protein interaction databases expand, the nature of the interrelationships will become clearer and the complete repertoire of small GTPase mediated processes will be known.

At this stage, there are many disconnected components in the small GTPases network suggesting that a substantial number of interactions remain to be identified. It may be that the network will eventually be entirely connected but it is also conceivable that at least some of these disconnected components represent diverging branches.

Scale-free behavior occurs in a wide variety of networks including non biological systems such as the Internet. [38], [27] Interestingly, this type of network is often found in cellular systems involving protein and gene interactions as well. [37] Scale-free networks typically have a few highly connected nodes and many other nodes that engage in a relatively smaller number of interactions. [38], [40] Because of this, scale-free networks are believed to be robust against accidental failure but highly vulnerable to a coordinated attack. [128] Scale-free networks are thought to arise from the preferential attachment scheme or the “rich get richer” paradigm which is based on the idea that the probability is greater for a new node to connect with a node that already has many attachments. [38] This implies that highly connected nodes will continue to grow at a faster rate than nodes with fewer connections. This is certainly the case for the Internet. [127] However, biological networks are thought to grow by a different process. Partial duplication for example is one model that explains the growth and evolution of biological networks. [54], [55], [129]–[131] According to the theory of gene duplication, the duplication event may result in a new gene copy that is free from selective pressure. The newly copied gene could acquire mutations that encode for a novel protein which may retain the same or similar functionality and interactions as the original protein product or may diverge substantially and gain new functions and interacting partners. [132] The partial duplication model mimics the natural process of gene duplication which has long been considered a major driving force in evolution. Some of the nodes and connections are duplicated in full, while others are duplicated partially. The availability of comprehensive protein-protein interaction and whole genome micro array data sets derived from studies with the yeast *Saccharomyces cerevisiae* has made it possible to analyze the growth properties of gene expression and protein-protein interaction networks. [133], [134] Results from these modeling studies indicate that a portion of the nodes and connections are duplicated in full, while others are duplicated partially which generates a scale free graph that has a degree exponent consistent with that observed for the small GTPases network. [54], [135] Importantly, network growth by full duplication of its components does not result in a power distribution of connectivities. [54] Another relevant and interesting characteristic of growth by partial duplication is that the number of connections multiplies at a faster rate than the number of proteins in the network. [54] This is consistent with the higher interaction to protein ratio observed in the small GTPases network and is believed to be most efficient evolutionarily as the cell would need to expend more energy in order to produce entirely new proteins as opposed to modifying already existing ones. The net result is a larger gain of function at less cost.

In summary, a small GTPases protein interaction network was constructed based on experimentally validated interactions. The network has a scale-free topology and is largely held together by members of the Rho and Ras subfamilies. Visualization of the network architecture and spatial organization offers a global view of the circuitry underlying the signaling routes governed by these enigmatic proteins. This information, together with the identification of potential higher order protein complexes and emerging pathways, may offer valuable guidance for further exploration.

## Supporting Information

Figure S1 **Network motifs.** Representative clusters (6, 7, 8, and 9) with biological themes that were identified in the network. The highest scoring (seed) nodes in the cluster are shown as squares.
(TIF)

Table S1 **Literature validation.**
(DOCX)

Table S2 **Proteins in the small GTPases network.**
(DOCX)

Table S3 **Proteins present in multiple small GTPase networks.**
(DOCX)

Table S4 **Interactions of proteins present in multiple small GTPase networks.**
(DOCX)

Graph S1 **GTPase network.**
(ZIP)

Spreadsheet S1 **GTPase search.**
(XLSX)

Spreadsheet S2 **Predicted protein signatures.**
(XLSX)

Spreadsheet S3 **KEGG pathway mapping.**
(XLSX)
