# Supplementary figures and images for: Topological and Functional Properties of the Small GTPases Protein Interaction Network
Source: PLoS One. 2012 Sep 13;7(9):e44882. doi: 10.1371/journal.pone.0044882 (PMC3441499; doi:10.1371/journal.pone.0044882)

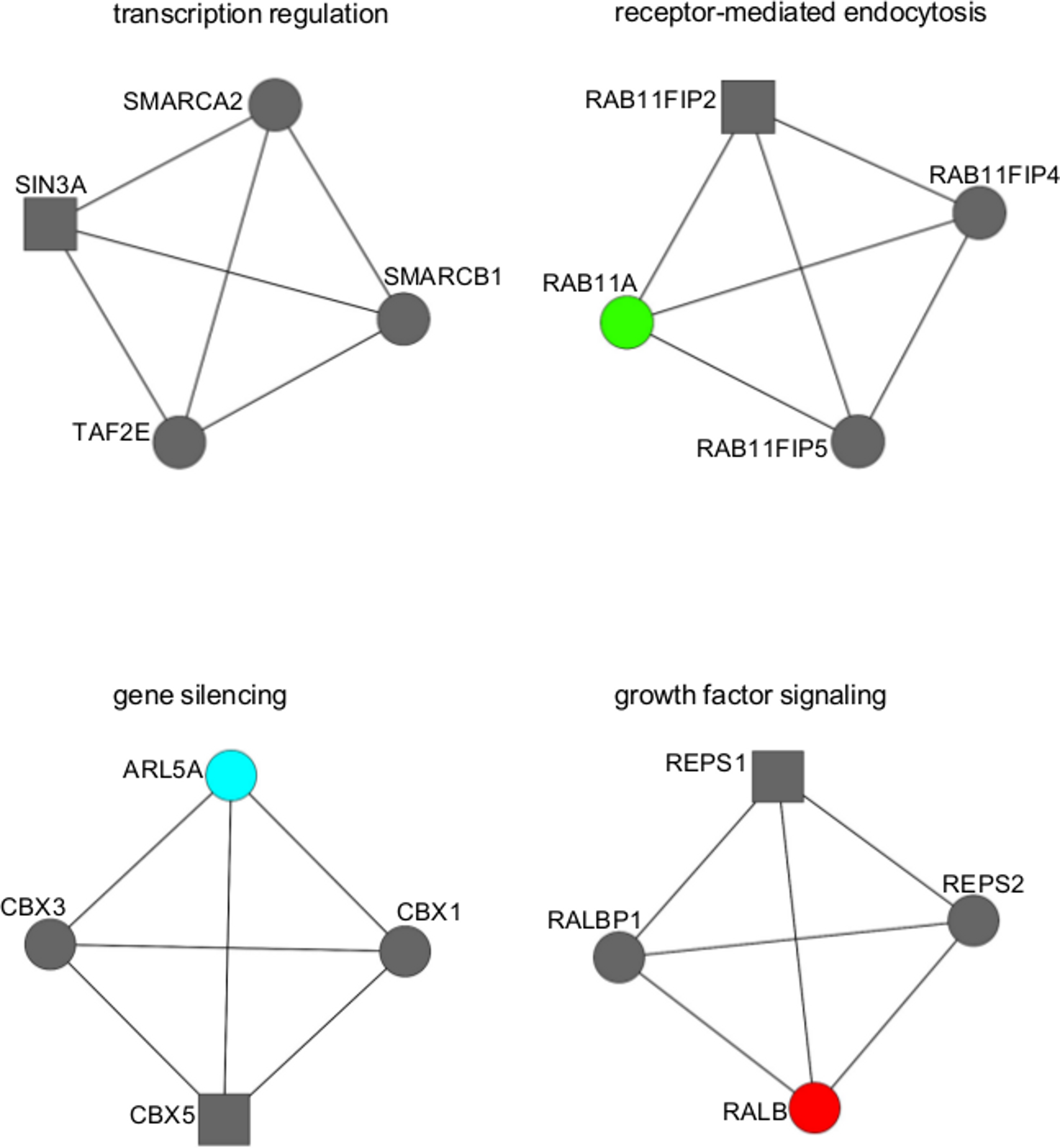

Supplement: Figure S1 — Network motifs. Representative clusters (6, 7, 8, and 9) with biological themes that were identified in the network. The highest scoring (seed) nodes in the cluster are shown as squares. (TIF) [file pone.0044882.s001.tif]
