# Supplementary material for: Topological and Functional Properties of the Small GTPases Protein Interaction Network
Source: PLoS One. 2012 Sep 13;7(9):e44882. doi: 10.1371/journal.pone.0044882 (PMC3441499; doi:10.1371/journal.pone.0044882)
Supplement: Table S1 — Literature validation. (DOCX) [file pone.0044882.s002.docx]

| **Table S1. Literature validation** | |
| --- | --- |
| **GTPase** | **Reference** |
| **RAC1** |  |
| ABI2 | The WAVE regulatory complex is inhibited. Nat Struct Mol Biol (2009). |
| ACTA1 | Direct interaction of actin with p47(phox) of neutrophil NADPH oxidase. Biochem Biophys Res Commun (2000). |
| ACTB | Direct interaction of actin with p47(phox) of neutrophil NADPH oxidase. Biochem Biophys Res Commun (2000).  The composition of Staufen - containing RNA granules from human cells indicates their role in the regulated transport and translation of messenger RNAs. Nucleic Acids Res (2004). |
| AKT1 | Akt protein kinase inhibits Rac1 - GTP binding through phosphorylation at serine 71 of Rac1. J Biol Chem (2000). |
| ARFFIP1 | The structural basis of Arfaptin - mediated cross-talk between Rac and Arf signalling pathways. Nature (2001). |
| ARFFIP2 | Differential binding of arfaptin 2/POR1 to ADP-ribosylation factors and Rac1. Biochem Biophys Res Commun (2001).  A role for POR1, a Rac1 - interacting protein, in ARF6 - mediated cytoskeletal rearrangements. EMBO J (1997).  The structural basis of Arfaptin - mediated cross-talk between Rac and Arf signalling pathways. Nature (2001).  Identification of a novel Rac1 - interacting protein involved in membrane ruffling. EMBO J (1996). |
| ARHGAP1 | Interaction of Rac1 with GTPase - activating proteins and putative effectors. A comparison with Cdc42 and RhoA. J Biol Chem (1998). |
| ARHGAP15 | ArhGAP15, a novel human RacGAP protein with GTPase binding property. FEBS Lett (2003). |
| ARHGAP17 | Rich, a rho GTPase - activating protein domain-containing protein involved in signaling by Cdc42 and Rac1. J Biol Chem (2001). |
| ARHGAP31 | A human MAP kinase interactome. Nat Methods (2010).  CdGAP, a novel proline-rich GTPase - activating protein for Cdc42 and Rac. J Biol Chem (1998). |
| ARHGAP32 | GC-GAP, a Rho family GTPase - activating protein that interacts with signaling adapters Gab1 and Gab2. J Biol Chem (2003).  Characterization of a brain-specific Rho GTPase - activating protein, p200RhoGAP. J Biol Chem (2003). |
| ARHGAP33 | A human MAP kinase interactome. Nat Methods (2010). |
| ARHGAP44 | Rich, a rho GTPase - activating protein domain-containing protein involved in signaling by Cdc42 and Rac1. J Biol Chem (2001). |
| ARHGDIA | Mapping the binding site for the GTP-binding protein Rac-1 on its inhibitor RhoGDI-1. Structure (2000).  Crystal structure of the Rac1 - RhoGDI complex involved in nadph oxidase activation. Biochemistry (2001).  Mechanism of NADPH oxidase activation by the Rac/Rho-GDI complex. Biochemistry (2001).  Interactions between Rho GTPases and Rho GDP dissociation inhibitor (Rho-GDI). Biochimie (2001).  Rac1 - induced cell migration requires membrane recruitment of the nuclear oncogene SET. EMBO J (2007).  Differential properties of D4 / LyGDI versus RhoGDI: phosphorylation and rho GTPase selectivity. FEBS Lett (1998).  The interaction between rac1 and its guanine nucleotide dissociation inhibitor (GDI), monitored by a single fluorescent coumarin attached to GDI. Biochemistry (1999).  Large-scale mapping of human protein-protein interactions by mass spectrometry. Mol Syst Biol (2007). |
| ARHGDIB | Stimulation of phospholipase C-beta2 by the Rho GTPases Cdc42Hs and Rac1. EMBO J (1998). |
| ARHGEF19 | WGEF activates Rho in the Wnt - PCP pathway and controls convergent extension in Xenopus gastrulation. EMBO J (2008). |
| ARHGEF2 | Trp(56) of rac1 specifies interaction with a subset of guanine nucleotide exchange factors. J Biol Chem (2001)  Cloning and characterization of GEF-H1, a microtubule-associated guanine nucleotide exchange factor for Rac and Rho GTPases. J Biol Chem (1998). |
| ARHGEF25 | A Rac/Cdc42-specific exchange factor, GEFT, induces cell proliferation, transformation, and migration. J Biol Chem (2003). |
| ARHGEF4 | Asef, a link between the tumor suppressor APC and G-protein signaling. Science (2000).  A human MAP kinase interactome. Nat Methods (2010). |
| ARHGEF7 | Basic fibroblast growth factor stimulates activation of Rac1 through a p85 betaPIX phosphorylation-dependent pathway. J Biol Chem (2004). |
| ARL2BP | A human MAP kinase interactome. Nat Methods (2010). |
| BAIAP2 | IRSp53 is an essential intermediate between Rac and WAVE in the regulation of membrane ruffling. Nature (2000).  WAVE2 serves a functional partner of IRSp53 by regulating its interaction with Rac. Biochem Biophys Res Commun (2002). |
| BCR | Interaction of Rac1 with GTPase - activating proteins and putative effectors. A comparison with Cdc42 and RhoA.  J Biol Chem (1998). |
| CASP3, CASP7 | Cdc42 is a substrate for caspases and influences Fas-induced apoptosis. J Biol Chem (2001). |
| CAV1 | Caveolin-1 is essential for activation of Rac1 and NAD(P)H oxidase after angiotensin II type 1 receptor stimulation in vascular smooth muscle cells: role in redox signaling and vascular hypertrophy. Arterioscler Thromb Vasc Biol (2005). |
| CCDC104 | A human MAP kinase interactome. Nat Methods (2010). |
| CDC42BPG | Expression of the human myotonic dystrophy kinase - related Cdc42 - binding kinase gamma is regulated by promoter DNA methylation and Sp1 binding. J Biol Chem (2004). |
| CDC42SE1, CDC42SE2 | SPECs, small binding proteins for Cdc42. J Biol Chem (2000). |
| CHN1 | A novel functional target for tumor-promoting phorbol esters and lysophosphatidic acid. The p21rac-GTPase activating protein n-chimaerin. J Biol Chem (1993). |
| CHN2 | Phospholipase Cgamma/diacylglycerol-dependent activation of beta2-chimaerin restricts EGF - induced Rac signaling. EMBO J (2006). |
| CIT | A novel partner for the GTP-bound forms of rho and rac. FEBS Lett (1995). |
| CLIP1 | Rac1 and Cdc42 capture microtubules through IQGAP1 and CLIP-170. Cell (2002). |
| CNTNAP1 | Interaction of Rac1 with GTPase - activating proteins and putative effectors. A comparison with Cdc42 and RhoA.  J Biol Chem (1998). |
| CYBA | Rac1 disrupts p67phox / p40phox binding: a novel role for Rac in NADPH oxidase activation. Biochem Biophys Res Commun (1999). |
| CYBB | Intracellular localization and preassembly of the NADPH oxidase complex in cultured endothelial cells. J Biol Chem (2002). |
| CYFIP1 | The WAVE regulatory complex is inhibited. Nat Struct Mol Biol (2009).  p140Sra-1 (specifically Rac1 - associated protein) is a novel specific target for Rac1 small GTPase.  J Biol Chem (1998). |
| DEF6 | DEF6, a novel PH-DH-like domain protein, is an upstream activator of the Rho GTPases Rac1, Cdc42, and RhoA.  Exp Cell Res (2004). |
| DIAPH1 | The formin/diaphanous-related protein, FHOS, interacts with Rac1 and activates transcription from the serum response element. J Biol Chem (2001). |
| DMPK | Rac-1 and Raf-1 kinases, components of distinct signaling pathways, activate myotonic dystrophy protein kinase.  FEBS Lett (2000) |
| DOCK1 | Activation of Rac1 by a Crk SH3 - binding protein, DOCK180. Genes Dev (1998).  Unconventional Rac - GEF activity is mediated through the Dock180 - ELMO complex. Nat Cell Biol (2002).  PH domain of ELMO functions in trans to regulate Rac activation via Dock180. Nat Struct Mol Biol (2004). |
| DOCK8 | Isolation and charachtarization of DOCK8, a member of the DOCK180 - related regulators of cell morphology.  FEBS Lett (2004).  DOCK8 regulates interstitial DC migration by spatially controlling Cdc42 activity. DOCK8 KO FUKUI, Y 2012 BLOOD |
| DVL1, DVL2 | Coactivation of Rac and Rho by Wnt / Frizzled signaling is required for vertebrate gastrulation. Genes Dev (2003). |
| EIF2AK2 | A 68-kDa kinase and NADPH oxidase component p67phox are targets for Cdc42Hs and Rac1 in neutrophils.  J Biol Chem (1995). |
| ELMO1 | PH domain of ELMO functions in trans to regulate Rac activation via Dock180. Nat Struct Mol Biol (2004). |
| FHOD1 | The formin/diaphanous-related protein, FHOS, interacts with Rac1 and activates transcription from the serum response element. J Biol Chem (2001).  Formin homology domain protein (FHOD1) is a cyclic GMP-dependent protein kinase I-binding protein and substrate in vascular smooth muscle cells. J Biol Chem (2004).  Oligomerization of the diaphanous - related formin FHOD1 requires a coiled-coil motif critical for its cytoskeletal and transcriptional activities. FEBS Lett (2005). |
| FLNA | *Filamin A regulates monocyte migration through Rho small GTPases during osteoclastogenesis. J Bone Miner Res 2010 |
| FMNL1 | FRL, a novel formin - related protein, binds to Rac and regulates cell motility and survival of macrophages.  Mol Cell Biol (2000). |
| FRK | Semaphorin-4A, an activator for T-cell-mediated immunity, suppresses angiogenesis via Plexin-D1. EMBO J (2007). |
| HPS4 | A human MAP kinase interactome. Nat Methods (2010) |
| ICMT | Role of isoprenylcysteine carboxyl methyltransferase in tumor necrosis factor-alpha stimulation of expression of vascular cell adhesion molecule-1 in endothelial cells. Arterioscler Thromb Vasc Biol (2002). |
| IFNGR1 | Rac1 contributes to maximal activation of STAT1 and STAT3 in IFN-gamma - stimulated rat astrocytes.  J Immunol (2004). |
| IL1RAP | Transactivation by the p65 subunit of NF-kappaB in response to interleukin-1 (IL-1) involves MyD88, IL-1 receptor-associated kinase 1, TRAF-6, and Rac1. Mol Cell Biol (2001). |
| IQGAP1^1-6^, IQGAP2^4,7^ | ^1^Identification of IQGAP as a putative target for the small GTPases, Cdc42 and Rac1. J Biol Chem (1996).  ^2^IQGAP1, a calmodulin - binding protein with a rasGAP - related domain, is a potential effector for cdc42Hs.EMBO J (1996).  ^3^Identification of a putative effector for Cdc42Hs with high sequence similarity to the RasGAP - related protein IQGAP1 and a Cdc42Hs binding partner with similarity to IQGAP2. J Biol Chem (1996).  ^4^Identification of protein-protein interactions using in vivo cross-linking and mass spectrometry. Proteomics (2004).  ^5^Rac1 and Cdc42 capture microtubules through IQGAP1 and CLIP-170. Cell (2002).  ^6^Interaction of Rac1 with GTPase - activating proteins and putative effectors. A comparison with Cdc42 and RhoA. J Biol Chem (1998).  ^7^The Ras GTPase-activating-protein - related human protein IQGAP2 harbors a potential actin binding domain and interacts with calmodulin and Rho family GTPases. Mol Cell Biol (1996). |
| KALRN | Kalirin, a cytosolic protein with spectrin - like and GDP/GTP exchange factor-like domains that interacts with peptidylglycine alpha-amidating monooxygenase, an integral membrane peptide-processing enzyme. J Biol Chem (1997). |
| KIAA0209 | Non-adherent cell-specific expression of DOCK2, a member of the human CDM - family proteins. Biochim Biophys Acta (1999).  Unconventional Rac - GEF activity is mediated through the Dock180 - ELMO complex. Nat Cell Biol (2002). |
| KPNA4 | A human MAP kinase interactome. Nat Methods (2010). |
| KPNA6 | A human MAP kinase interactome. Nat Methods (2010). |
| KTN1 | Interaction of the Rho family small G proteins with kinectin, an anchoring protein of kinesin motor.  Biochem Biophys Res Commun (1996). |
| LATS1 | A human MAP kinase interactome. Nat Methods (2010). |
| MAGI1 | A human MAP kinase interactome. Nat Methods (2010). |
| MAP3K10 | The MAP kinase kinase kinase MLK2 co-localizes with activated JNK along microtubules and associates with kinesin superfamily motor KIF3. EMBO J (1998). |
| MAP3K11 | The MAP kinase kinase kinase MLK2 co-localizes with activated JNK along microtubules and associates with kinesin superfamily motor KIF3. EMBO J (1998). |
| MAP3K4 | Cloning of a novel mitogen - activated protein kinase kinase kinase, MEKK4, that selectively regulates the c-Jun amino terminal kinase pathway. J Biol Chem (1997).  MEK kinases are regulated by EGF and selectively interact with Rac / Cdc42. EMBO J (1997). |
| MCF2L | Pleckstrin homology domain-mediated activation of the rho - specific guanine nucleotide exchange factor Dbs by Rac1. J Biol Chem (2004).  A novel oncogene, ost, encodes a guanine nucleotide exchange factor that potentially links Rho and Rac signaling pathways. EMBO J (1994). |
| METAP2 | A 68-kDa kinase and NADPH oxidase component p67phox are targets for Cdc42Hs and Rac1 in neutrophils.  J Biol Chem (1995). |
| MTNR1A | Purification and identification of G protein-coupled receptor protein complexes under native conditions.  Mol Cell Proteomics (2007). |
| MYD88 | Transactivation by the p65 subunit of NF-kappaB in response to interleukin-1 (IL-1) involves MyD88, IL-1 receptor-associated kinase 1, TRAF-6, and Rac1. Mol Cell Biol (2001). |
| NCF1 | Signal transduction by Rac small G proteins in phagocytes. C R Seances Soc Biol Fil (1997). |
| NCF2 | Cryptic Rac - binding and p21(Cdc42Hs/Rac)-activated kinase phosphorylation sites of NADPH oxidase component p67(phox). J Biol Chem (1998).  A 68-kDa kinase and NADPH oxidase component p67phox are targets for Cdc42Hs and Rac1 in neutrophils.  J Biol Chem (1995).  Phagocyte NADPH oxidase p67-phox possesses a novel carboxylterminal binding site for the GTPases Rac2 and Cdc42. Biochem Biophys Res Commun (1998).  Structure of the TPR domain of p67phox in complex with Rac.GTP. Mol Cell (2000). |
| NCK1 | The Drosophila HEM-2 / NAP1 homolog KETTE controls axonal path finding and cytoskeletal organization.  Genes Dev (2000). |
| NCKAP1 | The WAVE regulatory complex is inhibited. Nat Struct Mol Biol (2009).  Interaction of Nck - associated protein 1 with activated GTP-binding protein Rac. Biochem J (1997).  p140Sra-1 (specifically Rac1 - associated protein) is a novel specific target for Rac1 small GTPase.  J Biol Chem (1998). |
| NCKIPSD | SPIN90 - IRSp53 complex participates in Rac - induced membrane ruffling. Exp Cell Res (2009). |
| NGEF | WGEF activates Rho in the Wnt - PCP pathway and controls convergent extension in Xenopus gastrulation.  EMBO J (2008).  Regulation of ephexin1, a guanine nucleotide exchange factor of Rho family GTPases, by fibroblast growth factor receptor-mediated tyrosine phosphorylation. J Biol Chem (2007). |
| NME1 | Tumor metastasis suppressor nm23H1 regulates Rac1 GTPase by interaction with Tiam1. Proc Natl Acad Sci U S A (2001). |
| NOS2 | Specific association of nitric oxide synthase - 2 with Rac isoforms in activated murine macrophages. Am J Physiol Renal Physiol (2001).  Endothelial nitric oxide synthase and caveolin-1 are co-localized in sinusoidal endothelial fenestrae. Liver (2001). |
| NOXA1 | Novel human homologues of p47phox and p67phox participate in activation of superoxide - producing NADPH oxidases. J Biol Chem (2003). |
| OCRL | Lowe syndrome protein OCRL1 interacts with Rac GTPase in the trans-Golgi network. Hum Mol Genet (2003). |
| OPHN1 | Oligophrenin-1 encodes a rhoGAP protein involved in X-linked mental retardation. Nature (1998).  Rho proteins, mental retardation and the cellular basis of cognition. Trends Neurosci (2002). |
| PAK1 | ArhGAP15, a novel human RacGAP protein with GTPase binding property. FEBS Lett (2003).  A human MAP kinase interactome. Nat Methods (2010)  Rac1 - induced cell migration requires membrane recruitment of the nuclear oncogene SET. EMBO J (2007).  14-3-3zeta Cooperates with ErbB2 to promote ductal carcinoma in situ progression to invasive breast cancer by inducing epithelial-mesenchymal transition. Cancer Cell (2009).  Localized Rac activation dynamics visualized in living cells. Science (2000).  Interaction of Rac1 with GTPase - activating proteins and putative effectors. A comparison with Cdc42 and RhoA. J Biol Chem (1998).  A novel serine kinase activated by rac1/CDC42Hs-dependent autophosphorylation is related to PAK65 and STE20. EMBO J (1995).  RhoG activates Rac1 by direct interaction with the Dock180 - binding protein Elmo. Nature (2003). |
| PAK2 | Interaction of Rac1 with GTPase - activating proteins and putative effectors. A comparison with Cdc42 and RhoA. J Biol Chem (1998).  POSH2 is a RING finger E3 ligase with Rac1 binding activity through a partial CRIB domain. FEBS Lett (2010).  A human MAP kinase interactome. Nat Methods (2010). |
| PAK3 | Differential binding of arfaptin 2/POR1 to ADP-ribosylation factors and Rac1. Biochem Biophys Res Commun (2001). |
| PAK4, PAK5, PAK7 | a new brain-specific kinase, promotes neurite outgrowth in N1E-115 cells. Mol Cell Biol (2002) |
| PARD6A | A human homolog of the C. elegans polarity determinant Par-6 links Rac and Cdc42 to PKCzeta signaling and cell transformation. Curr Biol (2000).  Human homologues of the Caenorhabditis elegans cell polarity protein PAR6 as an adaptor that links the small GTPases Rac and Cdc42 to atypical protein kinase C. Genes Cells (2001).  PAR-6-PAR-3 mediates Cdc42 - induced Rac activation through the Rac GEFs STEF / Tiam1. Nat Cell Biol (2005). |
| PARD6B, PARD6G | Human homologues of the Caenorhabditis elegans cell polarity protein PAR6 as an adaptor that links the small GTPases Rac and Cdc42 to atypical protein kinase C. Genes Cells (2001). |
| PIK3R1, PIP4K2A | Rho family GTPases bind to phosphoinositide kinases. J Biol Chem (1995). |
| PLCB2 | Crystal structure of Rac1 bound to its effector phospholipase C-beta2. Nat Struct Mol Biol (2006). |
| PLD1 | Antigen-stimulated activation of phospholipase D1b by Rac1, ARF6, and PKCalpha in RBL - 2H3 cells. Mol Biol Cell (2002). |
| PLKHG2 | Activation of clg, a novel dbl family guanine nucleotide exchange factor gene, by proviral insertion at evi24, a common integration site in B cell and myeloid leukemias. J Biol Chem (2002). |
| PLXNB1 | Plexin-B1 directly interacts with PDZ-RhoGEF / LARG to regulate RhoA and growth cone morphology. Neuron (2002). |
| PPP2R2B | Rac1 - induced cell migration requires membrane recruitment of the nuclear oncogene SET. EMBO J (2007). |
| PRKCA | Interaction of protein kinase C isozymes with Rho GTPases. Biochemistry (2001).  Phospholipase Cgamma/diacylglycerol-dependent activation of beta2-chimaerin restricts EGF - induced Rac signaling. EMBO J (2006). |
| PRKCD | Celastrol binds to ERK and inhibits FcepsilonRI signaling to exert an anti-allergic effect. Eur J Pharmacol (2009). |
| PRKCI | Human homologues of the Caenorhabditis elegans cell polarity protein PAR6 as an adaptor that links the small GTPases Rac and Cdc42 to atypical protein kinase C. Genes Cells (2001).  PAR-6-PAR-3 mediates Cdc42 - induced Rac activation through the Rac GEFs STEF / Tiam1. Nat Cell Biol (2005). |
| PTK2 | A human MAP kinase interactome. Nat Methods (2010). |
| PTPLAD1 | B-ind1, a novel mediator of Rac1 signaling cloned from sodium butyrate - treated fibroblasts. J Biol Chem (2000). |
| RAB35 | Rab35 regulates neurite outgrowth and cell shape. FEBS Lett (2009). |
| RALBP1 | Bridging Ral GTPase to Rho pathways. RLIP76, a Ral effector with CDC42 / Rac GTPase - activating protein activity. J Biol Chem (1995). |
| RAP1GDS1 | A human MAP kinase interactome. Nat Methods (2010).  SmgGDS displays differential binding and exchange activity towards different Ras isoforms. Oncogene (2002). |
| RASGRF1 | G protein beta gamma subunit-dependent Rac - guanine nucleotide exchange activity of Ras-GRF1 / CDC25(Mm). Proc Natl Acad Sci U S A (1999). |
| RCC2 | The mammalian passenger protein TD-60 is an RCC1 family member with an essential role in prometaphase to metaphase progression. Dev Cell (2003). |
| RGL2 | Identification of a novel RalGDS - related protein as a candidate effector for Ras and Rap1.  J Biol Chem (1996). |
| RPS6KB1 | Visualization of biochemical networks in living cells. Proc Natl Acad Sci U S A (2001). |
| RTKN | WGEF activates Rho in the Wnt - PCP pathway and controls convergent extension in Xenopus gastrulation. EMBO J (2008). |
| SET | Rac1 - induced cell migration requires membrane recruitment of the nuclear oncogene SET.  EMBO J (2007). |
| SFPQ | A human MAP kinase interactome. Nat Methods (2010). |
| SH3BP1 | Sema3A - induced growth-cone collapse is mediated by Rac1 amino acids 17-32. Curr Biol (1999). |
| SH3D20 | Identification and characterization of a novel Rho GTPase activating protein implicated in receptor-mediated endocytosis. FEBS Lett (2004). |
| SH3FR1^1,2^, SH3FR3^2^ | A human MAP kinase interactome. Nat Methods (2010).  POSH2 is a RING finger E3 ligase with Rac1 binding activity through a partial CRIB domain. FEBS Lett (2010). |
| STAT1 | Rac1 contributes to maximal activation of STAT1 and STAT3 in IFN-gamma - stimulated rat astrocytes.  J Immunol (2004). |
| STAT3 | Regulation of STAT3 by direct binding to the Rac1 GTPase. Science (2000). |
| STAU1 | The composition of Staufen - containing RNA granules from human cells indicates their role in the regulated transport and translation of messenger RNAs. Nucleic Acids Res (2004). |
| SYNJ2 | Synaptojanin 2, a novel Rac1 effector that regulates clathrin - mediated endocytosis. Curr Biol (2000).  A human MAP kinase interactome. Nat Methods (2010). |
| TBC1D3F | The TRE17 oncogene encodes a component of a novel effector pathway for Rho GTPases Cdc42 and Rac1 and stimulates actin remodeling. Mol Cell Biol (2003). |
| TEC | Activation and association of the Tec tyrosine kinase with the human prolactin receptor: mapping of a Tec/Vav1-receptor binding site. Mol Endocrinol (2001). |
| TIAM1 | Toll-like receptor 2-mediated NF-kappa B activation requires a Rac1 - dependent pathway. Nat Immunol (2000). |
| TLR2 | Toll-like receptor 2-mediated NF-kappa B activation requires a Rac1 - dependent pathway. Nat Immunol (2000). |
| TNF | Toll-like receptor 2-mediated NF-kappa B activation requires a Rac1 - dependent pathway. Nat Immunol (2000). |
| TNFRSF12A | Fibroblast growth factor-inducible-14 is induced in axotomized neurons and promotes neurite outgrowth. J Neurosci (2003). |
| TRIO | Trp(56) of rac1 specifies interaction with a subset of guanine nucleotide exchange factors. J Biol Chem (2001). |
| TUBA4A | The Ras - related GTPase Rac1 binds tubulin. J Biol Chem (1996). |
| VAV1^1-3^, VAV2^3^ | ^1^Vav-Rac1-mediated activation of the c-Jun N-terminal kinase/c-Jun/AP-1 pathway plays a major role in stimulation of the distal NFAT site in the interleukin-2 gene promoter. Mol Cell Biol (2001).  ^2^Crucial structural role for the PH and C1 domains of the Vav1 exchange factor. EMBO Rep (2008).  ^3^Activation of Vav / Rho GTPase signaling by CXCL12 controls membrane-type matrix metalloproteinase-dependent melanoma cell invasion. Cancer Res (2006). |
| VAV3 | Biological and regulatory properties of Vav-3, a new member of the Vav family of oncoproteins. Mol Cell Biol (1999). |
| WAS | Direct interaction of the Wiskott-Aldrich syndrome protein with the GTPase Cdc42. Proc Natl Acad Sci U S A (1996). |
| WASF1 | The WAVE regulatory complex is inhibited. Nat Struct Mol Biol (2009).  WAVE, a novel WASP - family protein involved in actin reorganization induced by Rac. EMBO J (1998). |
| **RHOA** |  |
| AGER | Advanced glycation end products increase endothelial permeability through the RAGE / Rho signaling pathway.  FEBS Lett (2010). |
| AKAP13 | Ht31: the first protein kinase A anchoring protein to integrate protein kinase A and Rho signaling. FEBS Lett (2001).  Characterization of the interactions between the small GTPase RhoA and its guanine nucleotide exchange factors. Anal Biochem (2002). |
| ARHGAP1 | MgF(3)(-) as a transition state analog of phosphoryl transfer. Chem Biol (2002).  Regulation of RhoA GTP hydrolysis by the GTPase - activating proteins p190, p50RhoGAP, Bcr, and 3BP-1. Biochemistry (1998).  Structural determinants required for the interaction between Rho GTPase and the GTPase - activating domain of p190. J Biol Chem (1997).  The BNIP-2 and Cdc42GAP homology (BCH) domain of p50RhoGAP / Cdc42GAP sequesters RhoA from inactivation by the adjacent GTPase - activating protein domain. Mol Biol Cell (2010).  Structure at 1.65 A of RhoA and its GTPase - activating protein in complex with a transition-state analogue. Nature (1997).  Interaction of Rac1 with GTPase - activating proteins and putative effectors. A comparison with Cdc42 and RhoA. J Biol Chem (1998).  Towards a proteome-scale map of the human protein-protein interaction network. Nature (2005). |
| ARHGAP10 | PKNbeta interacts with the SH3 domains of Graf and a novel Graf related protein, Graf2, which are GTPase activating proteins for Rho family. J Biochem (2001). |
| ARHGAP21 | Golgi-localized GAP for Cdc42 functions downstream of ARF1 to control Arp2/3 complex and F - actin dynamics.  Nat Cell Biol (2005). |
| ARHGAP26 | PKNbeta interacts with the SH3 domains of Graf and a novel Graf related protein, Graf2, which are GTPase activating proteins for Rho family. J Biochem (2001). |
| ARHGAP32 | p250GAP, a novel brain-enriched GTPase - activating protein for Rho family GTPases, is involved in the N-methyl-d-aspartate receptor signaling. Mol Biol Cell (2003).  Characterization of a brain-specific Rho GTPase - activating protein, p200RhoGAP. J Biol Chem (2003).  Grit, a GTPase - activating protein for the Rho family, regulates neurite extension through association with the TrkA receptor and N-Shc and CrkL / Crk adapter molecules. Mol Cell Biol (2002). |
| ARHGAP5 | Structural determinants required for the interaction between Rho GTPase and the GTPase - activating domain of p190. J Biol Chem (1997).  Rnd proteins function as RhoA antagonists by activating p190 RhoGAP. Curr Biol (2003). |
| ARHGDIA | How RhoGDI binds Rho. Acta Crystallogr D Biol Crystallogr (1999).  Differential localization of Rho GTPases in live cells: regulation by hypervariable regions and RhoGDI binding. J Cell Biol (2001).  Interactions between Rho GTPases and Rho GDP dissociation inhibitor (Rho-GDI). Biochimie (2001).  Cytoskeleton-mediated death receptor and ligand concentration in lipid rafts forms apoptosis-promoting clusters in cancer chemotherapy. J Biol Chem (2005).  Differential properties of D4 / LyGDI versus RhoGDI: phosphorylation and rho GTPase selectivity. FEBS Lett (1998).  Large-scale mapping of human protein-protein interactions by mass spectrometry. Mol Syst Biol (2007). |
| ARHGDIB | Stimulation of phospholipase C-beta2 by the Rho GTPases Cdc42Hs and Rac1. EMBO J (1998). |
| ARHGDIG | RhoGDIgamma: a GDP-dissociation inhibitor for Rho proteins with preferential expression in brain and pancreas.  Proc Natl Acad Sci U S A (1997). |
| ARHGEF1 | Hyaluronan - mediated CD44 interaction with RhoGEF and Rho kinase promotes Grb2 - associated binder-1 phosphorylation and phosphatidylinositol 3-kinase signaling leading to cytokine (macrophage-colony stimulating factor) production and breast tumor progression. J Biol Chem (2003). |
| ARHGEF11 | The crystal structure of RhoA in complex with the DH / PH fragment of PDZRhoGEF, an activator of the Ca(2+) sensitization pathway in smooth muscle. Structure (2004).  Rho - specific binding and guanine nucleotide exchange catalysis by KIAA0380, a dbl family member. FEBS Lett (1999).  Activated RhoA binds to the pleckstrin homology (PH) domain of PDZ-RhoGEF, a potential site for autoregulation. J Biol Chem (2010). |
| ARHGEF12 | Structural determinants of RhoA binding and nucleotide exchange in leukemia-associated Rho guanine - nucleotide exchange factor. J Biol Chem (2004).  Galpha 12 activates Rho GTPase through tyrosine-phosphorylated leukemia-associated RhoGEF.  Proc Natl Acad Sci U S A (2003).  Leukemia-associated Rho guanine nucleotide exchange factor, a Dbl family protein found mutated in leukemia, causes transformation by activation of RhoA. J Biol Chem (2001). |
| ARHGEF18 | Identification and characterization of a novel Rho - specific guanine nucleotide exchange factor. Biochem J (2000). |
| ARHGEF19 | WGEF activates Rho in the Wnt - PCP pathway and controls convergent extension in Xenopus gastrulation. EMBO J (2008). |
| ARHGEF2 | Cloning and characterization of GEF-H1, a microtubule-associated guanine nucleotide exchange factor for Rac and Rho GTPases. J Biol Chem (1998). |
| ARHGEF25 | p63RhoGEF and GEFT are Rho - specific guanine nucleotide exchange factors encoded by the same gene. Naunyn Schmiedebergs Arch Pharmacol (2004).  Structure of Galphaq-p63RhoGEF-RhoA complex reveals a pathway for the activation of RhoA by GPCRs. Science (2007). |
| ARHGEF3 | XPLN, a guanine nucleotide exchange factor for RhoA and RhoB, but not RhoC. J Biol Chem (2002). |
| ARHGEF4 | Asef, a link between the tumor suppressor APC and G-protein signaling. Science (2000). |
| ARHGEF5 | Structural basis for the selective activation of Rho GTPases by Dbl exchange factors. Nat Struct Biol (2002). |
| BCR | Interaction of Rac1 with GTPase - activating proteins and putative effectors. A comparison with Cdc42 and RhoA. J Biol Chem (1998). |
| BID | Cytoskeleton-mediated death receptor and ligand concentration in lipid rafts forms apoptosis-promoting clusters in cancer chemotherapy. J Biol Chem (2005). |
| BPGAP1 | Concerted regulation of cell dynamics by BNIP-2 and Cdc42GAP homology/Sec14p-like, proline-rich, and GTPase - activating protein domains of a novel Rho GTPase - activating protein, BPGAP1. J Biol Chem (2003). |
| CASP10, CASP8 | Cytoskeleton-mediated death receptor and ligand concentration in lipid rafts forms apoptosis-promoting clusters in cancer chemotherapy. J Biol Chem (2005). |
| CAV1 | Localization of RhoA GTPase to endothelial caveolae-enriched membrane domains. Biochem Biophys Res Commun (1998). |
| CIT | RhoE binds to ROCK I and inhibits downstream signaling. Mol Cell Biol (2003).  A novel partner for the GTP-bound forms of rho and rac. FEBS Lett (1995). |
| CNKSR1 | Human CNK1 acts as a scaffold protein, linking Rho and Ras signal transduction pathways. Mol Cell Biol (2004).  Bioluminescence resonance energy transfer identify scaffold protein CNK1 interactions in intact cells. FEBS Lett (2005). |
| CNTNAP1 | Interaction of Rac1 with GTPase - activating proteins and putative effectors. A comparison with Cdc42 and RhoA. J Biol Chem (1998).  Structural determinants required for the interaction between Rho GTPase and the GTPase - activating domain of p190. J Biol Chem (1997). |
| CRMP1 | p80 ROKalpha binding protein is a novel splice variant of CRMP-1 which associates with CRMP-2 and modulates RhoA - induced neuronal morphology. FEBS Lett (2002). |
| DAAM1 | Wnt / Frizzled activation of Rho regulates vertebrate gastrulation and requires a novel Formin homology protein Daam1. Cell (2001). |
| DEF6 | DEF6, a novel PH-DH-like domain protein, is an upstream activator of the Rho GTPases Rac1, Cdc42, and RhoA. Exp Cell Res (2004). |
| DGKQ | Diacylglycerol kinase theta binds to and is negatively regulated by active RhoA. J Biol Chem (1999). |
| DIAPH1 | RhoE binds to ROCK I and inhibits downstream signaling. Mol Cell Biol (2003).  Structural and mechanistic insights into the interaction between Rho and mammalian Dia. Nature (2005). |
| DOCK7 | Identification of an evolutionarily conserved superfamily of DOCK180 - related proteins with guanine nucleotide exchange activity. J Cell Sci (2002). |
| DPYSL2 | p80 ROKalpha binding protein is a novel splice variant of CRMP-1 which associates with CRMP-2 and modulates RhoA - induced neuronal morphology. FEBS Lett (2002). |
| DVL2 | Coactivation of Rac and Rho by Wnt / Frizzled signaling is required for vertebrate gastrulation. Genes Dev (2003). |
| EZR | Cytoskeleton-mediated death receptor and ligand concentration in lipid rafts forms apoptosis-promoting clusters in cancer chemotherapy. J Biol Chem (2005). |
| FADD, FAS, FASLG | Cytoskeleton-mediated death receptor and ligand concentration in lipid rafts forms apoptosis-promoting clusters in cancer chemotherapy. J Biol Chem (2005). |
| FLNA | The small GTPase RalA targets filamin to induce filopodia. Proc Natl Acad Sci U S A (1999). |
| GMIP | A novel Rho GTPase-activating-protein interacts with Gem, a member of the Ras superfamily of GTPases.  Biochem J (2002). |
| GNA12 | Galpha 12 activates Rho GTPase through tyrosine-phosphorylated leukemia-associated RhoGEF. Proc Natl Acad Sci U S A (2003). |
| GRLF1 | Regulation of RhoA GTP hydrolysis by the GTPase - activating proteins p190, p50RhoGAP, Bcr, and 3BP-1.  Biochemistry (1998). |
| HSPA1A | Protective role of HSP72 against Clostridium difficile toxin A - induced intestinal epithelial cell dysfunction.  Am J Physiol Cell Physiol (2003). |
| ICMT | Mammalian prenylcysteine carboxyl methyltransferase is in the endoplasmic reticulum. J Biol Chem (1998). |
| ITPR1 | RhoA interaction with inositol 1,4,5-trisphosphate receptor and transient receptor potential channel-1 regulates Ca2+ entry. Role in signaling increased endothelial permeability. J Biol Chem (2003). |
| KCNA2 | The small GTP-binding protein RhoA regulates a delayed rectifier potassium channel. Cell (1998). |
| KTN1 | RhoE binds to ROCK I and inhibits downstream signaling. Mol Cell Biol (2003).  Interaction of the Rho family small G proteins with kinectin, an anchoring protein of kinesin motor. Biochem Biophys Res Commun (1996).  PIST: a novel PDZ/coiled-coil domain binding partner for the rho - family GTPase TC10. Biochem Biophys Res Commun (2001).  Kinectin is a key effector of RhoG microtubule-dependent cellular activity. Mol Cell Biol (2001). |
| MAPK8 | Cytoskeleton-mediated death receptor and ligand concentration in lipid rafts forms apoptosis-promoting clusters in cancer chemotherapy. J Biol Chem (2005). |
| MCF2 | Identification of Rho GTPase - dependent sites in the Dbl homology domain of oncogenic Dbl that are required for transformation. J Biol Chem (2000). |
| MPRIP | Identification of a novel, putative Rho - specific GDP/GTP exchange factor and a RhoA - binding protein: control of neuronal morphology. J Cell Biol (1997).  Myosin phosphatase-Rho interacting protein. A new member of the myosin phosphatase complex that directly binds RhoA. J Biol Chem (2003). |
| MSN | Cytoskeleton-mediated death receptor and ligand concentration in lipid rafts forms apoptosis-promoting clusters in cancer chemotherapy. J Biol Chem (2005). |
| NET1 | Activation of clg, a novel dbl family guanine nucleotide exchange factor gene, by proviral insertion at evi24, a common integration site in B cell and myeloid leukemias. J Biol Chem (2002). |
| NGEF | WGEF activates Rho in the Wnt - PCP pathway and controls convergent extension in Xenopus gastrulation.  EMBO J (2008). |
| OPHN1 | Oligophrenin-1 encodes a rhoGAP protein involved in X-linked mental retardation. Nature (1998).  Rho proteins, mental retardation and the cellular basis of cognition. Trends Neurosci (2002). |
| OTUB1 | Post-translational modification of the deubiquitinating enzyme otubain 1 modulates active RhoA levels and susceptibility to Yersinia invasion. FEBS J (2010). |
| PDE6D | The delta subunit of retinal rod cGMP phosphodiesterase regulates the membrane association of Ras and Rap GTPases. J Biol Chem (2002). |
| PITPNM1 | Mitotic phosphorylation of the peripheral Golgi protein Nir2 by Cdk1 provides a docking mechanism for Plk1 and affects cytokinesis completion. Mol Cell (2004). |
| PKN1 | RhoE binds to ROCK I and inhibits downstream signaling. Mol Cell Biol (2003).  Analysis of RhoA - binding proteins reveals an interaction domain conserved in heterotrimeric G protein beta subunits and the yeast response regulator protein Skn7. J Biol Chem (1998).  Multiple interactions of PRK1 with RhoA. Functional assignment of the Hr1 repeat motif. J Biol Chem (1998).  Identification of a putative target for Rho as the serine-threonine kinase protein kinase N. Science (1996).  The structural basis of Rho effector recognition revealed by the crystal structure of human RhoA complexed with the effector domain of PKN / PRK1. Mol Cell (1999). |
| PKN2 | Isolation of a NCK - associated kinase, PRK2, an SH3 - binding protein and potential effector of Rho protein signaling. J Biol Chem (1996)  The PRK2 kinase is a potential effector target of both Rho and Rac GTPases and regulates actin cytoskeletal organization. Mol Cell Biol (1997).  Multiple interactions of PRK1 with RhoA. Functional assignment of the Hr1 repeat motif. J Biol Chem (1998). |
| PLCG1 | Leukotriene D4 induces association of active RhoA with phospholipase C-gamma1 in intestinal epithelial cells.  Biochem J (2002). |
| PLD1 | Activation of phospholipase D1 by ADP-ribosylated RhoA. Biochem Biophys Res Commun (2003).  Determination of interaction sites of phospholipase D1 for RhoA. Biochem J (2001).  Determination of interaction sites on the small G protein RhoA for phospholipase D. J Biol Chem (1998). |
| PLXNB1 | Semaphorin 4D signaling requires the recruitment of phospholipase C gamma into the plexin-B1 receptor complex. Mol Cell Biol (2009). |
| PPP1R12A | Regulation of myosin phosphatase through phosphorylation of the myosin - binding subunit in platelet activation.  Blood (1997). |
| PRKACA | Serine phosphorylation negatively regulates RhoA in vivo. J Biol Chem (2003). |
| PRKCA | Interaction of protein kinase C isozymes with Rho GTPases. Biochemistry (2001). |
| PRKCZ | The low molecular weight GTPase RhoA and atypical protein kinase Czeta are required for TLR2 - mediated gene transcription. J Immunol (2004). |
| RABAC1 | Prenylated Rab acceptor protein is a receptor for prenylated small GTPases. J Biol Chem (2001). |
| RAP1GDS1 | SmgGDS displays differential binding and exchange activity towards different Ras isoforms. Oncogene (2002). |
| RASGRF1 | G protein beta gamma subunit-dependent Rac - guanine nucleotide exchange activity of Ras-GRF1 / CDC25(Mm). Proc Natl Acad Sci U S A (1999). |
| RGNEF | Identification of a novel, putative Rho - specific GDP/GTP exchange factor and a RhoA - binding protein: control of neuronal morphology. J Cell Biol (1997). |
| RHOH | Bioluminescence resonance energy transfer identify scaffold protein CNK1 interactions in intact cells. FEBS Lett (2005). |
| RHPN1^1^, RHPN2^1,2^ | ^1^The RhoA - binding protein, rhophilin-2, regulates actin cytoskeleton organization. J Biol Chem (2002).  ^2^RhoE binds to ROCK I and inhibits downstream signaling. Mol Cell Biol (2003). |
| RIPK4 | Identification and characterization of a novel Rho - specific guanine nucleotide exchange factor. Biochem J (2000). |
| ROCK1 | RhoE binds to ROCK I and inhibits downstream signaling. Mol Cell Biol (2003).  Identification of the Rho - binding domain of p160ROCK, a Rho - associated coiled-coil containing protein kinase. J Biol Chem (1996).  Structural insights into the interaction of ROCKI with the switch regions of RhoA. J Biol Chem (2004). |
| ROCK2 | Role of transglutaminase II in retinoic acid-induced activation of RhoA - associated kinase - 2. EMBO J (2001). |
| RTKN | Activation of phospholipase D1 by ADP-ribosylated RhoA. Biochem Biophys Res Commun (2003).  Rhotekin, a new putative target for Rho bearing homology to a serine/threonine kinase, PKN, and rhophilin in the rho - binding domain. J Biol Chem (1996).  RhoG activates Rac1 by direct interaction with the Dock180 - binding protein Elmo. Nature (2003).  WGEF activates Rho in the Wnt - PCP pathway and controls convergent extension in Xenopus gastrulation. EMBO J (2008).  Semaphorin 4D signaling requires the recruitment of phospholipase C gamma into the plexin-B1 receptor complex. Mol Cell Biol (2009).  Advanced glycation end products increase endothelial permeability through the RAGE / Rho signaling pathway. FEBS Lett (2010). |
| SH3BP1, SMURF1 | Regulation of RhoA GTP hydrolysis by the GTPase - activating proteins p190, p50RhoGAP, Bcr, and 3BP-1. Biochemistry (1998). |
| SMURF2 | Autoinhibition of the HECT - type ubiquitin ligase Smurf2 through its C2 domain. Cell (2007).  Ubiquitination of RhoA by Smurf1 promotes neurite outgrowth. FEBS Lett (2005).  Degradation of RhoA by Smurf1 ubiquitin ligase. Methods Enzymol (2006). |
| SPRED2 | The Sprouty - related protein, Spred, inhibits cell motility, metastasis, and Rho - mediated actin reorganization.  Oncogene (2004). |
| SRGAP1 | Signal transduction in neuronal migration: roles of GTPase activating proteins and the small GTPase Cdc42 in the Slit - Robo pathway. Cell (2001). |
| TEC | Galpha 12 activates Rho GTPase through tyrosine-phosphorylated leukemia-associated RhoGEF. Proc Natl Acad Sci U S A (2003). |
| TGM2 | Role of transglutaminase II in retinoic acid-induced activation of RhoA - associated kinase - 2. EMBO J (2001). |
| TNFRSF10B, TNFRSF1A | Cytoskeleton-mediated death receptor and ligand concentration in lipid rafts forms apoptosis-promoting clusters in cancer chemotherapy. J Biol Chem (2005). |
| TRIO | The trio guanine nucleotide exchange factor is a RhoA target. Binding of RhoA to the trio immunoglobulin-like domain. J Biol Chem (2000). |
| TRPC1 | RhoA interaction with inositol 1,4,5-trisphosphate receptor and transient receptor potential channel-1 regulates Ca2+ entry. Role in signaling increased endothelial permeability. J Biol Chem (2003). |
| UBB^1^, UBC^1-3^ | ^1^Autoinhibition of the HECT - type ubiquitin ligase Smurf2 through its C2 domain. Cell (2007).  ^2^Ubiquitination of RhoA by Smurf1 promotes neurite outgrowth. FEBS Lett (2005).  ^3^Synaptopodin orchestrates actin organization and cell motility via regulation of RhoA signalling. Nat Cell Biol (2006). |
| VAV1 | Activation of Vav / Rho GTPase signaling by CXCL12 controls membrane-type matrix metalloproteinase-dependent melanoma cell invasion. Cancer Res (2006).  Crucial structural role for the PH and C1 domains of the Vav1 exchange factor. EMBO Rep (2008). |
| VAV2 | Critical but distinct roles for the pleckstrin homology and cysteine-rich domains as positive modulators of Vav2 signaling and transformation. Mol Cell Biol (2002). |
| VAV3 | Biological and regulatory properties of Vav-3, a new member of the Vav family of oncoproteins. Mol Cell Biol (1999). |
| **HRAS** |  |
| ABL2 | RIN1 is an ABL tyrosine kinase activator and a regulator of epithelial-cell adhesion and migration. Curr Biol (2005) |
| AGTR1 | Regulation of neuromodulatory actions of angiotensin II in the brain neurons by the Ras - dependent mitogen - activated protein kinase pathway. J Neurosci (1996). |
| ARAF | Novel raf kinase protein-protein interactions found by an exhaustive yeast two-hybrid analysis. Genomics (2003). |
| BCL2 | Bcl-2 differentially targets K-, N-, and H-Ras to mitochondria in IL-2 supplemented or deprived cells: implications in prevention of apoptosis. Oncogene (1999). |
| BRAF | Identification of signalling proteins interacting with B-Raf in the yeast two-hybrid system. Oncogene (1996).  Biochemical analysis of MEK activation in NIH3T3 fibroblasts. Identification of B-Raf and other activators. J Biol Chem (1995). |
| BRAP | Ras regulates assembly of mitogenic signalling complexes through the effector protein IMP. Nature (2004). |
| CAV1 | Co-purification and direct interaction of Ras with caveolin, an integral membrane protein of caveolae microdomains. Detergent-free purification of caveolae microdomains. J Biol Chem (1996). |
| CDC25A, CDC25B | Raf1 interaction with Cdc25 phosphatase ties mitogenic signal transduction to cell cycle activation. Genes Dev (1995). |
| CDC25C | Interaction of activated Ras with Raf-1 alone may be sufficient for transformation of rat2 cells. Mol Cell Biol (1997). |
| DGKZ | Diacylglycerol kinase zeta  regulates Ras activation by a novel mechanism. J Cell Biol (2001). |
| ERBB2IP | Erbin suppresses the MAP kinase pathway. J Biol Chem (2003).  Erbin inhibits RAF activation by disrupting the sur-8-Ras-Raf complex. J Biol Chem (2006). |
| FNTA, FNTB | Identification and preliminary characterization of protein-cysteine farnesyltransferase.  Proc Natl Acad Sci U S A (1990). |
| FYN | Fyn is induced by Ras/PI3K/Akt signaling and is required for enhanced invasion/migration. *Mol Carcinog* (2011). |
| GPSM2 | Identification of tetratricopeptide repeat 1 as an adaptor protein that interacts with heterotrimeric G proteins and the small GTPase Ras. Mol Cell Biol (2003). |
| GRB2 | Electroconvulsive shock increases the phosphorylation of Pyk2 in the rat hippocampus. Biochem Biophys Res Commun (2001).  Human Sos1: a guanine nucleotide exchange factor for Ras that binds to GRB2. Science (1993). |
| HGF | *Coupling of Gab1 to c-Met, Grb2, and Shp2 mediates biological responses. J. Cell Biol. (2000). |
| ICMT | Targeting Ras signaling through inhibition of carboxyl methylation: an unexpected property of methotrexate. Proc Natl Acad Sci U S A (2003). |
| IZKF3 | Aiolos transcription factor controls cell death in T cells by regulating Bcl-2 expression and its cellular localization. EMBO J (1999). |
| IL3 | Survival function of ERK1/2 as IL-3 - activated, staurosporine - resistant Bcl2 kinases. Proc Natl Acad Sci U S A (2000). |
| INSR | Interaction of the human insulin receptor with the ras oncogene product p21. FEBS Lett (1987). |
| IRAK1, IRAK2 | Ras participates in the activation of p38 MAPK by interleukin-1 by associating with IRAK, IRAK2, TRAF6, and TAK-1. J Biol Chem (2002). |
| ITSN1 | Intersectin activates Ras but stimulates transcription through an independent pathway involving JNK. J Biol Chem (2003). |
| LGALS1 | Galectin-1 binds oncogenic H-Ras to mediate Ras membrane anchorage and cell transformation.  Oncogene (2001). |
| MAPK8 | JNK1: a protein kinase stimulated by UV light and Ha-Ras that binds and phosphorylates the c-Jun activation domain. Cell (1994). |
| MLLT4 | The junctional multidomain protein AF-6 is a binding partner of the Rap1A GTPase and associates with the actin cytoskeletal regulator profilin. Proc Natl Acad Sci U S A (2000).  In vivo interaction of AF-6 with activated Ras and ZO-1. Biochem Biophys Res Commun (1999). |
| NF1 | Identification of neurofibromin mutants that exhibit allele specificity or increased Ras affinity resulting in suppression of activated ras alleles. Mol Cell Biol (1996). |
| PDE6D | The complex of Arl2 - GTP and PDE delta: from structure to function. EMBO J (2002).  The delta subunit of retinal rod cGMP phosphodiesterase regulates the membrane association of Ras and Rap GTPases. J Biol Chem (2002) |
| PDGFB | NF-kappaB is a target of AKT in anti-apoptotic PDGF signaling Nature (1999). |
| PIK3CA | The small GTP-binding protein, Rhes, regulates signal transduction from G protein-coupled receptors. Oncogene (2004).  The leucine-rich repeat protein SUR-8 enhances MAP kinase activation and forms a complex with Ras and Raf. Genes Dev (2000).  Activation of phosphoinositide 3-kinase by interaction with Ras and by point mutation. EMBO J (1996).  Role of phosphoinositide 3-OH kinase in cell transformation and control of the actin cytoskeleton by Ras. Cell (1997). |
| PIK3CD | The junctional multidomain protein AF-6 is a binding partner of the Rap1A GTPase and associates with the actin cytoskeletal regulator profilin. Proc Natl Acad Sci U S A (2000)  P110delta, a novel phosphoinositide 3-kinase in leukocytes. Proc Natl Acad Sci U S A (1997). |
| PIK3CG | Crystal structure and functional analysis of Ras binding to its effector phosphoinositide 3-kinase gamma. Cell (2000). |
| PIK3RI | A novel pathway for tumor necrosis factor-alpha and ceramide signaling involving sequential activation of tyrosine kinase, p21(ras), and phosphatidylinositol 3-kinase. J Biol Chem (1999)  Role of phosphoinositide 3-OH kinase in cell transformation and control of the actin cytoskeleton by Ras. Cell (1997). |
| PLAU | Myosin light chain kinase functions downstream of Ras / ERK to promote migration of urokinase - type plasminogen activator-stimulated cells in an integrin-selective manner. J Cell Biol (1999). |
| PLCE1 | Structural and mechanistic insights into ras association domains of phospholipase C epsilon. Mol Cell (2006).  A human protein-protein interaction network: a resource for annotating the proteome. Cell (2005). |
| PRKCI | Mapping of atypical protein kinase C within the nerve growth factor signaling cascade: relationship to differentiation and survival of PC12 cells. Mol Cell Biol (2000). |
| PRKCZ | Role of Ras/PKCzeta/MEK/ERK1/2 signaling pathway in angiotensin II-induced vascular smooth muscle cell proliferation. Regul Pept (2005). |
| RABAC | Prenylated Rab acceptor protein is a receptor for prenylated small GTPases. J Biol Chem (2001). |
| RAF1 | Interaction of Ras and Raf in intact mammalian cells upon extracellular stimulation. J Biol Chem (1994).  The ubiquitously expressed Syp phosphatase interacts with c-kit and Grb2 in hematopoietic cells. J Biol Chem (1994).  Regulation of Raf-1 by direct feedback phosphorylation. Mol Cell (2005).  SIAH-1 interacts with CtIP and promotes its degradation by the proteasome pathway. Oncogene (2003).  Downregulation of the Ras-mitogen-activated protein kinase pathway by the EphB2 receptor tyrosine kinase is required for ephrin - induced neurite retraction. Mol Cell Biol (2001).  14-3-3 zeta negatively regulates raf-1 activity by interactions with the Raf-1 cysteine-rich domain. J Biol Chem (1997).  Interaction of activated Ras with Raf-1 alone may be sufficient for transformation of rat2 cells. Mol Cell Biol (1997).  Identification and characterization of rain, a novel Ras - interacting protein with a unique subcellular localization. J Biol Chem (2004).  The leucine-rich repeat protein SUR-8 enhances MAP kinase activation and forms a complex with Ras and Raf. Genes Dev (2000). |
| RALA | *Activation of RalA is critical for Ras-induced tumorogenesis of human cells. Cancer Cell (2005).  *RalA suppresses early stages of Ras-induced squamous cell carcinoma progression. Oncogene (2010). |
| RALGDS | Activated Ras interacts with the Ral guanine nucleotide dissociation stimulator. Proc Natl Acad Sci U S A (1994).  Three-dimensional structure of the Ras - interacting domain of RalGDS. Nat Struct Biol (1997).  Structural basis for the interaction of Ras with RalGDS. Nat Struct Biol (1998).  The leucine-rich repeat protein SUR-8 enhances MAP kinase activation and forms a complex with Ras and Raf. Genes Dev (2000).  RalGDS functions in Ras- and cAMP-mediated growth stimulation. J Biol Chem (1997).  Identification of the guanine nucleotide dissociation stimulator for Ral as a putative effector molecule of R-ras, H-ras, K-ras, and Rap. Proc Natl Acad Sci U S A (1994).  Identification and characterization of rain, a novel Ras - interacting protein with a unique subcellular localization. J Biol Chem (2004).  The junctional multidomain protein AF-6 is a binding partner of the Rap1A GTPase and associates with the actin cytoskeletal regulator profilin. Proc Natl Acad Sci U S A (2000).  Activated Ras interacts with the Ral guanine nucleotide dissociation stimulator. Proc Natl Acad Sci U S A (1994).  Role of phosphoinositide 3-OH kinase in cell transformation and control of the actin cytoskeleton by Ras. Cell (1997).  A human MAP kinase interactome. Nat Methods (2010). |
| RAP1GDS | SmgGDS displays differential binding and exchange activity towards different Ras isoforms. Oncogene (2002). |
| RAPGEF1 | CrkL mediates Ras - dependent activation of the Raf / ERK pathway through the guanine nucleotide exchange factor C3G in hematopoietic cells stimulated with erythropoietin or interleukin-3. J Biol Chem (1999). |
| RARRES3 | RIG1 inhibits the Ras/mitogen-activated protein kinase pathway by suppressing the activation of Ras. Cell Signal (2006). |
| RASA1 | Refined crystal structure of the triphosphate conformation of H-ras p21 at 1.35 A resolution: implications for the mechanism of GTP hydrolysis. EMBO J (1990).  A cytoplasmic protein stimulates normal N-ras p21 GTPase, but does not affect oncogenic mutants. Science (1987).  Biological properties of human c-Ha-ras1 genes mutated at codon 12. Nature 1984  Formation of a transition-state analog of the Ras GTPase reaction by Ras - GDP, tetrafluoroaluminate, and GTPase - activating proteins. Science (1996).  Crystal structure of the GTPase - activating domain of human p120GAP and implications for the interaction with Ras. Nature (1996).  The Ras - RasGAP complex: structural basis for GTPase activation and its loss in oncogenic Ras mutants. Science (1997).  The interaction of Ras with GTPase - activating proteins. FEBS Lett (1997).  Ras binding to a C-terminal region of GAP. FEBS Lett (1995).  Guanosine triphosphatase activating protein (GAP) interacts with the p21 ras effector binding domain. Science (1988).  The Ras - RasGAP complex: structural basis for GTPase activation and its loss in oncogenic Ras mutants. Science (1997)  Differential actions of p60c-Src and Lck kinases on the Ras regulators p120-GAP and GDP/GTP exchange factor CDC25Mm. Eur J Biochem (2001). |
| RASGRF1 | Interaction of activated Ras with Raf-1 alone may be sufficient for transformation of rat2 cells.  Mol Cell Biol (1997). |
| RASGRP1 | RASGRP1, a Ras guanyl nucleotide- releasing protein with calcium- and diacylglycerol-binding motifs.  Science (1998). |
| RASGRP4 | RasGRP4 is a novel Ras activator isolated from acute myeloid leukemia. J Biol Chem (2002). |
| RASIP1 | Identification and characterization of rain, a novel Ras - interacting protein with a unique subcellular localization. J Biol Chem (2004). |
| RASSF1 | Ras uses the novel tumor suppressor RASSF1 as an effector to mediate apoptosis.  J Biol Chem (2000).  Novel type of Ras effector interaction established between tumour suppressor NORE1A and Ras switch II. EMBO J (2008).  The putative tumor suppressor RASSF1A homodimerizes and heterodimerizes with the Ras - GTP binding protein Nore1. Oncogene (2002). |
| RASSF2 | RASSF2 is a novel K-Ras - specific effector and potential tumor suppressor.  J Biol Chem (2003). |
| RASSF3 | Identification of Nore1 as a potential Ras effector. J Biol Chem (1998). |
| RASSF5 | Novel type of Ras effector interaction established between tumour suppressor NORE1A and Ras switch II. EMBO J (2008).  Identification of Nore1 as a potential Ras effector. J Biol Chem (1998). |
| RGL1 | A human MAP kinase interactome. Nat Methods (2010).  Biochemical characterization of the Ras - related GTPases Rit and Rin. Arch Biochem Biophys (1999). |
| RGL2 | Identification and characterization of rain, a novel Ras - interacting protein with a unique subcellular localization. J Biol Chem (2004).  Identification of a novel RalGDS - related protein as a candidate effector for Ras and Rap1. J Biol Chem (1996).  A human MAP kinase interactome. Nat Methods (2010). |
| RGL4 | Interaction of activated Ras with Raf-1 alone may be sufficient for transformation of rat2 cells.  Mol Cell Biol (1997).  rgr oncogene: activation by elimination of translational controls and mislocalization.  Cancer Res (2003). |
| RGS12 | Selective role for RGS12 as a Ras/Raf/MEK scaffold in nerve growth factor-mediated differentiation.  EMBO J (2007). |
| RHOD | The CD40 - induced signaling pathway in endothelial cells resulting in the overexpression of vascular endothelial growth factor involves Ras and phosphatidylinositol 3-kinase. J Immunol (2004). |
| RIN1 | Identification and characterization of rain, a novel Ras - interacting protein with a unique subcellular localization. J Biol Chem (2004).  A human protein selected for interference with Ras function interacts directly with Ras and competes with Raf1. Mol Cell Biol (1995).  Protein binding and signaling properties of RIN1 suggest a unique effector function.  Proc Natl Acad Sci U S A (1997).  RIN1 is an ABL tyrosine kinase activator and a regulator of epithelial-cell adhesion and migration.  Curr Biol (2005). |
| RIT2 | Biochemical characterization of the Ras - related GTPases Rit and Rin. Arch Biochem Biophys (1999). |
| SHOC2 | The leucine-rich repeat protein SUR-8 enhances MAP kinase activation and forms a complex with Ras and Raf. Genes Dev (2000).  SUR-8, a conserved Ras - binding protein with leucine-rich repeats, positively regulates Ras - mediated signaling in C. elegans. Cell (1998).  Erbin inhibits RAF activation by disrupting the sur-8-Ras-Raf complex. J Biol Chem (2006). |
| SNRPE | Interaction of activated Ras with Raf-1 alone may be sufficient for transformation of rat2 cells. Mol Cell Biol (1997). |
| SOS1 | Membrane-dependent signal integration by the Ras activator Son of sevenless. Nat Struct Mol Biol (2008).  Structural evidence for feedback activation by Ras.GTP of the Ras - specific nucleotide exchange factor SOS. Cell (2003).  Structural analysis of autoinhibition in the Ras activator Son of sevenless. Cell (2004).  Catalytic competence of the Ras-GEF domain of hSos1 requires intra-REM domain interactions mediated by phenylalanine 577. FEBS Lett (2006).  The structural basis of the activation of Ras by Sos. Nature (1998).  Basis for signaling specificity difference between Sos and Ras-GRF guanine nucleotide exchange factors. J Biol Chem (2001).  Regulation of Sos activity by intramolecular interactions. Mol Cell Biol (1998). |
| SOS2 | The structural basis of the activation of Ras by Sos. Nature (1998).  Human Sos1: a guanine nucleotide exchange factor for Ras that binds to GRB2. Science (1993).  Structural evidence for feedback activation by Ras.GTP of the Ras - specific nucleotide exchange factor SOS. Cell (2003). |
| SRC | H-Ras modulates N-methyl-D-aspartate receptor function via inhibition of Src tyrosine kinase activity. J Biol Chem (2003). |
| TIAM1 | Tiam1 mediates Ras activation of Rac by a PI(3)K-independent mechanism. Nat Cell Biol (2002). |
| TLR2 | Toll-like receptor 2 and mitogen- and stress-activated kinase 1 are effectors of Mycobacterium avium-induced cyclooxygenase-2 expression in macrophages. J Biol Chem (2004). |
| TLR9 | Ras participates in CpG oligodeoxynucleotide signaling through association with toll-like receptor 9 and promotion of interleukin-1 receptor-associated kinase / tumor necrosis factor receptor-associated factor 6 complex formation in macrophages. J Biol Chem (2003). |
| TP73 | p19ras interacts with and activates p73 by involving the MDM2 protein. J Biol Chem (2006). |
| TTC1 | Identification of tetratricopeptide repeat 1 as an adaptor protein that interacts with heterotrimeric G proteins and the small GTPase Ras. Mol Cell Biol (2003). |
| VAV1 | Molecular analysis of Ras activation by tyrosine phosphorylated Vav. Biochem Biophys Res Commun (1995). |
| ZBTB12 | Identification of a novel RalGDS - related protein as a candidate effector for Ras and Rap1. J Biol Chem (1996). |
| **RAN** |  |
| ABL1 | Specific association of Type I c-Abl with Ran GTPase in lipopolysaccharide-mediated differentiation. Oncogene (2001). |
| AR | The linkage of Kennedy's neuron disease to ARA24, the first identified androgen receptor polyglutamine region-associated coactivator. J Biol Chem (1999). |
| ASAP2 | Identification of a new Pyk2 target protein with Arf-GAP activity. Mol Cell Biol (1999). |
| CDKN1B | 14-3-3 suppresses the nuclear localization of threonine 157-phosphorylated p27(Kip1). EMBO J (2004). |
| ENSG00000206525 | Proteomics identification of nuclear Ran GTPase as an inhibitor of human VRK1 and VRK2 (vaccinia-related kinase) activities. Mol Cell Proteomics (2008). |
| EP300 | E1A deregulates the centrosome cycle in a Ran GTPase - dependent manner. Cancer Res (2003). |
| H2AFY | The histone variant mH2A1.1 interferes with transcription by down-regulating PARP-1 enzymatic activity. Genes Dev (2006). |
| IPO11 | Ribosomal protein L12 uses a distinct nuclear import pathway mediated by importin 11. Mol Cell Biol (2002).  The histones H2A / H2B and H3 / H4 are imported into the yeast nucleus by different mechanisms. Eur J Cell Biol (2004).  Kap120 functions as a nuclear import receptor for ribosome assembly factor Rpf1 in yeast. Mol Cell Biol (2006).  High-quality binary protein interaction map of the yeast interactome network. Science (2008).  Network organization of the human autophagy system. Nature (2010). |
| IPO5^1,2^, IPO7^2-4^ | ^1^Cloning and characterization of human karyopherin beta3. Proc Natl Acad Sci U S A (1997).  ^2^Network organization of the human autophagy system. Nature (2010).  ^3^A novel class of RanGTP binding proteins. J Cell Biol (1997).  ^4^Identification of two novel RanGTP-binding proteins belonging to the importin beta superfamily. J Biol Chem (2000). |
| KPNB1 | Network organization of the human autophagy system. Nature (2010).  An integrated workflow for charting the human interaction proteome: insights into the PP2A system. Mol Syst Biol (2009).  Sequence and characterization of cytoplasmic nuclear protein import factor p97. J Cell Biol (1995).  Dominant-negative mutants of importin-beta block multiple pathways of import and export through the nuclear pore complex. EMBO J (1997).  Facilitated nucleocytoplasmic shuttling of the Ran binding protein RanBP1. Mol Cell Biol (2000).  Structural view of the Ran-Importin beta interaction at 2.3 A resolution. Cell (1999).  Molecular interactions between the importin alpha/beta heterodimer and proteins involved in vertebrate nuclear protein import. J Mol Biol (1997).  Ran binding domains promote the interaction of Ran with p97/beta-karyopherin, linking the docking and translocation steps of nuclear import. J Biol Chem (1996). |
| MBP | Nercc1, a mammalian NIMA - family kinase, binds the Ran GTPase and regulates mitotic progression. Genes Dev (2002). |
| MIP | Structure of the nuclear transport complex karyopherin-beta2-Ran x GppNHp. Nature (1999). |
| NEK9 | Nercc1, a mammalian NIMA - family kinase, binds the Ran GTPase and regulates mitotic progression. Genes Dev (2002).  Network organization of the human autophagy system. Nature (2010). |
| NR3C1 | Differential regulation of glucocorticoid receptor transcriptional activation via AF-1 - associated proteins. EMBO J (1999). |
| NUP153 | Nup153 is an M9 - containing mobile nucleoporin with a novel Ran-binding domain. EMBO J (1999). |
| NUP50 | Npap60 / Nup50 is a tri-stable switch that stimulates importin-alpha:beta-mediated nuclear protein import. Cell (2002). |
| NUTF2 | Structural basis for molecular recognition between nuclear transport factor 2 (NTF2) and the GDP-bound form of the Ras-family GTPase Ran. J Mol Biol (1998)  The 1.6 angstroms resolution crystal structure of nuclear transport factor 2 (NTF2). J Mol Biol (1996).  14-3-3 suppresses the nuclear localization of threonine 157-phosphorylated p27(Kip1). EMBO J (2004).  Computational and biochemical identification of a nuclear pore complex binding site on the nuclear transport carrier NTF2. J Mol Biol (2004). |
| NXF1 | Karyopherin beta 2B participates in mRNA export from the nucleus. Proc Natl Acad Sci U S A (2002). |
| NXT1 | Identification of an NTF2 - related factor that binds Ran-GTP and regulates nuclear protein export. Mol Cell Biol (1999). |
| PPP2R2B | An integrated workflow for charting the human interaction proteome: insights into the PP2A system. Mol Syst Biol (2009).  Network organization of the human autophagy system. Nature (2010). |
| PTMA | Identification of nuclear-import and cell-cycle regulatory proteins that bind to prothymosin alpha. Biochem Cell Biol (2001). |
| RANBP1 | RNA1 encodes a GTPase - activating protein specific for Gsp1p, the Ran/TC4 homologue of Saccharomyces cerevisiae. J Biol Chem (1995).  The mammalian Mog1 protein is a guanine nucleotide release factor for Ran. J Biol Chem (2000).  Separate domains of the Ran GTPase interact with different factors to regulate nuclear protein import and RNA processing. Mol Cell Biol (1995).  Mutations in the nuclear export signal of human ran-binding protein RanBP1 block the Rev-mediated posttranscriptional regulation of human immunodeficiency virus type 1. J Biol Chem (1997).  Facilitated nucleocytoplasmic shuttling of the Ran binding protein RanBP1. Mol Cell Biol (2000).  Co-activation of RanGTPase and inhibition of GTP dissociation by Ran-GTP binding protein RanBP1. EMBO J (1995). |
| RANBP10 | A novel MET-interacting protein shares high sequence similarity with RanBPM, but fails to stimulate MET-induced Ras / Erk signaling.  Biochem Biophys Res Commun (2004). |
| RANBP2 | A giant nucleopore protein that binds Ran/TC4. Nature (1995).  Structure of a Ran-binding domain complexed with Ran bound to a GTP analogue: implications for nuclear transport. Nature (1999).  Network organization of the human autophagy system. Nature (2010).  Two distinct classes of Ran-binding sites on the nucleoporin Nup-358. Proc Natl Acad Sci U S A (1999). |
| RANBP3 | Human RanBP3, a group of nuclear RanGTP binding proteins. FEBS Lett (1998).  Ran-binding protein 3 links Crm1 to the Ran guanine nucleotide exchange factor. J Biol Chem (2002). |
| RANBP9 | When overexpressed, a novel centrosomal protein, RanBPM, causes ectopic microtubule nucleation similar to gamma-tubulin.  J Cell Biol (1998). |
| RANGAP1 | Systematic identification of protein complexes in Saccharomyces cerevisiae by mass spectrometry. Nature (2002).  The crystal structure of rna1p: a new fold for a GTPase - activating protein. Mol Cell (1999).  RanGAP1 induces GTPase activity of nuclear Ras - related Ran. Proc Natl Acad Sci U S A (1994)  Network organization of the human autophagy system. Nature (2010).  RNA1 encodes a GTPase - activating protein specific for Gsp1p, the Ran/TC4 homologue of Saccharomyces cerevisiae. J Biol Chem (1995). |
| RANGRF | The mammalian Mog1 protein is a guanine nucleotide release factor for Ran. J Biol Chem (2000).  Identification and characterization of the human MOG1 gene. Gene (2001).  Systematic identification of protein complexes in Saccharomyces cerevisiae by mass spectrometry. Nature (2002). |
| RCC1 | Structural basis for guanine nucleotide exchange on Ran by the regulator of chromosome condensation (RCC1). Cell (2001).  Model of the ran-RCC1 interaction using biochemical and docking experiments. J Mol Biol (1999).  A mutant form of the Ran/TC4 protein disrupts nuclear function in Xenopus laevis egg extracts by inhibiting the RCC1 protein, a regulator of chromosome condensation. EMBO J (1994).  Proteomics identification of nuclear Ran GTPase as an inhibitor of human VRK1 and VRK2 (vaccinia-related kinase) activities. Mol Cell Proteomics (2008).  The mammalian Mog1 protein is a guanine nucleotide release factor for Ran. J Biol Chem (2000).  Separate domains of the Ran GTPase interact with different factors to regulate nuclear protein import and RNA processing. Mol Cell Biol (1995).  Co-activation of RanGTPase and inhibition of GTP dissociation by Ran-GTP binding protein RanBP1. EMBO J (1995).  Mitotic regulator protein RCC1 is complexed with a nuclear ras - related polypeptide. Proc Natl Acad Sci U S A (1991). |
| RGPD5 | Characterization and potential function of a novel testis-specific nucleoporin BS-63. Mol Reprod Dev (2002).  Network organization of the human autophagy system. Nature (2010). |
| RPL7 | Importin Î²3 mediates the nuclear import of human ribosomal protein L7 through its interaction with the multifaceted basic clusters of L7. FEBS Lett (2010).RPTOR  Analysis of proteins copurifying with the CD4 / lck complex using one-dimensional polyacrylamide gel electrophoresis and mass spectrometry: comparison with affinity-tag based protein detection and evaluation of different solubilization methods. J Am Soc Mass Spectrom (2004).  Network organization of the human autophagy system. Nature (2010). |
| RPTOR | Structure of the nuclear transport complex karyopherin-beta2-Ran x GppNHp. Nature (1999).  Network organization of the human autophagy system. Nature (2010). |
| SIN3A | ALL-1 is a histone methyltransferase that assembles a supercomplex of proteins involved in transcriptional regulation. Mol Cell (2002). |
| SMAD2 | Smad2 nucleocytoplasmic shuttling by nucleoporins CAN/Nup214 and Nup153 feeds TGFbeta signaling complexes in the cytoplasm and nucleus. Mol Cell (2002). |
| SMARCA2 | ALL-1 is a histone methyltransferase that assembles a supercomplex of proteins involved in transcriptional regulation. Mol Cell (2002). |
| SMARCB1 | A masked NES in INI1 / hSNF5 mediates hCRM1-dependent nuclear export: implications for tumorigenesis. EMBO J (2002).  Network organization of the human autophagy system. Nature (2010)  Complementary quantitative proteomics reveals that transcription factor AP-4 mediates E-box-dependent complex formation for transcriptional repression of HDM2. Mol Cell Proteomics (2009). |
| SNUPN | CRM1 - mediated recycling of snurportin 1 to the cytoplasm. J Cell Biol (1999).  NES consensus redefined by structures of PKI-type and Rev-type nuclear export signals bound to CRM1. Nat Struct Mol Biol (2010).  Crystal structure of the nuclear export receptor CRM1 in complex with Snurportin1 and RanGTP. Science (2009). |
| SPAG8 | Sperm membrane protein (hSMP-1) and RanBPM complex in the microtubule-organizing centre. J Mol Med (2004). |
| SSRP1 | Nercc1, a mammalian NIMA - family kinase, binds the Ran GTPase and regulates mitotic progression. Genes Dev (2002).  Network organization of the human autophagy system. Nature (2010).  Analysis of proteins copurifying with the CD4 / lck complex using one-dimensional polyacrylamide gel electrophoresis and mass spectrometry: comparison with affinity-tag based protein detection and evaluation of different solubilization methods. J Am Soc Mass Spectrom (2004). |
| TNPO1 | Structure of the nuclear transport complex karyopherin-beta2-Ran x GppNHp. Nature (1999).  Network organization of the human autophagy system. Nature (2010).  Analysis of proteins copurifying with the CD4 / lck complex using one-dimensional polyacrylamide gel electrophoresis and mass spectrometry: comparison with affinity-tag based protein detection and evaluation of different solubilization methods. J Am Soc Mass Spectrom (2004).  Karyopherin beta 2B participates in mRNA export from the nucleus. Proc Natl Acad Sci U S A (2002).  Identification of two novel RanGTP-binding proteins belonging to the importin beta superfamily. J Biol Chem (2000). |
| TNPO2 | Karyopherin beta 2B participates in mRNA export from the nucleus. Proc Natl Acad Sci U S A (2002). |
| VRK2, VRK3 | Proteomics identification of nuclear Ran GTPase as an inhibitor of human VRK1 and VRK2 (vaccinia-related kinase) activities. Mol Cell Proteomics (2008). |
| XPO1 | The coiled coil region (amino acids 129-250) of the tumor suppressor protein adenomatous polyposis coli (APC). Its structure and its interaction with chromosome maintenance region 1 (Crm-1). J Biol Chem (2002).  Facilitated nucleocytoplasmic shuttling of the Ran binding protein RanBP1. Mol Cell Biol (2000).  Network organization of the human autophagy system. Nature (2010).  CRM1 is an export receptor for leucine-rich nuclear export signals. Cell (1997). |
| XPO4 | Exportin 4: a mediator of a novel nuclear export pathway in higher eukaryotes. EMBO J (2000). |
| XPO5 | Exportin-5, a novel karyopherin, mediates nuclear export of double-stranded RNA binding proteins. J Cell Biol (2002).  A genetic defect in exportin-5 traps precursor microRNAs in the nucleus of cancer cells. Cancer Cell (2010).  Network organization of the human autophagy system. Nature (2010). |
| XPO6 | Exportin 6: a novel nuclear export receptor that is specific for profilin.actin complexes. EMBO J (2003). |
| XPO7 | Identification of two novel RanGTP-binding proteins belonging to the importin beta superfamily. J Biol Chem (2000).  Network organization of the human autophagy system. Nature (2010). |
| XPOT | Steady-state nuclear localization of exportin-t involves RanGTP binding and two distinct nuclear pore complex interaction domains. Mol Cell Biol (2002).  Identification of two novel RanGTP-binding proteins belonging to the importin beta superfamily. J Biol Chem (2000).  Network organization of the human autophagy system. Nature (2010).  Identification of a tRNA - specific nuclear export receptor. Mol Cell (1998). |
| RAB5A |  |
| AGTR1 | Rab5 association with the angiotensin II type 1A receptor promotes Rab5 GTP binding and vesicular fusion.  J Biol Chem (2002). |
| ALS2CL | ALS2CL, the novel protein highly homologous to the carboxy-terminal half of ALS2, binds to Rab5 and modulates endosome dynamics. FEBS Lett (2004). |
| ANKFY1 | The Rab5 effector Rabankyrin-5 regulates and coordinates different endocytic mechanisms. PLoS Biol (2004). |
| AP1G1, AP1G2 | Definition of the consensus motif recognized by gamma-adaptin ear domains.  J Biol Chem (2004). |
| APPL1 | APPL proteins link Rab5 to nuclear signal transduction via an endosomal compartment. Cell (2004).  Structure of the APPL1 BAR-PH domain and characterization of its interaction with Rab5. EMBO J (2007). |
| CHM | REP-2, a Rab escort protein encoded by the choroideremia-like gene. J Biol Chem (1994). |
| CHML | Mechanism of Rab geranylgeranylation: formation of the catalytic ternary complex. Biochemistry (1998).  REP-2, a Rab escort protein encoded by the choroideremia-like gene. J Biol Chem (1994). |
| EEA1 | Structural basis for Rab GTPase recognition and endosome tethering by the C2H2 zinc finger of Early Endosomal Autoantigen 1 (EEA1) Proc Natl Acad Sci U S A (2010).  Identification of rabaptin-5, rabex-5, and GM130 as putative effectors of rab33b, a regulator of retrograde traffic between the Golgi apparatus and ER. FEBS Lett (2001).  Membrane targeting and activation of the Lowe syndrome protein OCRL1 by rab GTPases. EMBO J (2006).  The small GTPase Rab22 interacts with EEA1 and controls endosomal membrane trafficking. J Cell Sci (2002).  Direct interaction of EEA1 with Rab5b. Eur J Biochem (1999)  Visualization of Rab5 activity in living cells by FRET microscopy and influence of plasma-membrane-targeted Rab5 on clathrin - dependent endocytosis. J Cell Sci (2003).  FYVE and coiled-coil domains determine the specific localisation of Hrs to early endosomes. J Cell Sci (2001).  EEA1 links PI(3)K function to Rab5 regulation of endosome fusion. Nature (1998). |
| GDI2 | General role of GDP dissociation inhibitor 2 in membrane release of Rab proteins: modulations of its functional interactions by in vitro and in vivo structural modifications. Biochemistry (1999). |
| GGA1, GGA2, GGA3 | Definition of the consensus motif recognized by gamma-adaptin ear domains. J Biol Chem (2004). |
| ITGB1 | Small GTPase Rab21 regulates cell adhesion and controls endosomal traffic of beta1-integrins. J Cell Biol (2006). |
| OCRL | Membrane targeting and activation of the Lowe syndrome protein OCRL1 by rab GTPases. EMBO J (2006). |
| RAB37 | Rab37 is a novel mast cell specific GTPase localized to secretory granules. FEBS Lett (2000) |
| RAB4A | Distinct Rab - binding domains mediate the interaction of Rabaptin-5 with GTP-bound Rab4 and Rab5. EMBO J (1998). |
| RAB5C | Large-scale mapping of human protein-protein interactions by mass spectrometry. Mol Syst Biol (2007). |
| RABAC1 | Interaction cloning and characterization of the cDNA encoding the human prenylated rab acceptor (PRA1). Biochem Biophys Res Commun (1999). |
| RABGGTB | Rab geranylgeranyl transferase catalyzes the geranylgeranylation of adjacent cysteines in the small GTPases Rab1A, Rab3A, and Rab5A.Proc Natl Acad Sci USA (1994) |
| RASA1 | Tyrosine phosphorylation of p190 RhoGAP by Fyn regulates oligodendrocyte differentiation. J Neurobiol (2001). |
| RIN1 | Ras - activated endocytosis is mediated by the Rab5 guanine nucleotide exchange activity of RIN1. Dev Cell (2001).  Rin1 interacts with signal-transducing adaptor molecule (STAM) and mediates epidermal growth factor receptor trafficking and degradation. J Biol Chem (2007). |
| RIN2^1,2^, Rin3^2^ | ^1^A novel binding protein composed of homophilic tetramer exhibits unique properties for the small GTPase Rab5. J Biol Chem (2002).  ^2^RIN3: a novel Rab5 GEF interacting with amphiphysin II involved in the early endocytic pathway. J Cell Sci (2003). |
| SDCBP | Role of Unc51.1 and its binding partners in CNS axon outgrowth. Genes Dev (2004). |
| STX4 | Rab5a GTPase regulates fusion between pathogen-containing phagosomes and cytoplasmic organelles in human neutrophils. J Cell Sci (2002). |
| TBC1D3F | The TRE17 oncogene encodes a component of a novel effector pathway for Rho GTPases Cdc42 and Rac1 and stimulates actin remodeling. Mol Cell Biol (2003). |
| TM9SF4 | The human homologue of Dictyostelium discoideum phg1A is expressed by human metastatic melanoma cells. EMBO Rep (2009). |
| TSC2 | Multicompartmental distribution of the tuberous sclerosis gene products, hamartin and tuberin. Arch Biochem Biophys (2002).  The tuberous sclerosis 2 gene product, tuberin, functions as a Rab5 GTPase activating protein (GAP) in modulating endocytosis. J Biol Chem (1997). |
| USP6NL | The Eps8 protein coordinates EGF receptor signalling through Rac and trafficking through Rab5. Nature (2000). |
| VPS45^1^, ZFYVE20^1,2^ | Rabenosyn-5, a novel Rab5 effector, is complexed with hVPS45 and recruited to endosomes through a FYVE finger domain. J Cell Biol (2000).  Structural basis of family-wide Rab GTPase recognition by rabenosyn-5. Nature (2005). |
| **ARF1** |  |
| AP1B1^1^, AP1G1^1^, AP3B1^1^, AP3S2^1^, AP3D1^1,2^ | ^1^Site-specific cross-linking reveals a differential direct interaction of class 1, 2, and 3 ADP-ribosylation factors with adaptor protein complexes 1 and 3. Biochemistry, 2002  ^2^Network organization of the human autophagy system. Nature 2010 |
| AP4E1, AP4M1 | Functional and physical interactions of the adaptor protein complex AP-4 with ADP-ribosylation factors (ARFs). EMBO 2001 |
| ARFGAP1 | Requirement for both the amino-terminal catalytic domain and a noncatalytic domain for in vivo activity of ADP-ribosylation factor GTPase - activating protein. J Biol Chem (1998). |
| ARFFIP1, ARFFIP1 | Arfaptin 1, a putative cytosolic target protein of ADP-ribosylation factor, is recruited to Golgi membranes. J Biol Chem (1997). |
| ARHGAP21 | Golgi-localized GAP for Cdc42 functions downstream of ARF1 to control Arp2/3 complex and F - actin dynamics. Nat Cell Biol (2005). |
| ASAP1, ASAP2 | AlFx affects the formation of focal complexes by stabilizing the Arf-GAP ASAP1 in a complex with Arf1. FEBS Lett (2005). |
| CHRM3 | ADP-ribosylation factor-dependent phospholipase D activation by the M3 muscarinic receptor. J Biol Chem (2003). |
| COPB1^1,2,3,4^  COPE^3,4^ | ^1^Site-specific photocrosslinking to probe interactions of Arf1 with proteins involved in budding of COPI vesicles. Methods (2000).  ^2^GTP-dependent binding of ADP-ribosylation factor to coatomer in close proximity to the binding site for dilysine retrieval motifs and p23. J Biol Chem (1999).  ^3^Network organization of the human autophagy system. Nature (2010).  ^4^COP I domains required for coatomer integrity, and novel interactions with ARF and ARF-GAP. EMBO J (2000). |
| CYTH1 | Solution structure of the cytohesin-1 (B2-1) Sec7 domain and its interaction with the GTPase ADP ribosylation factor 1. Proc Natl Acad Sci U S A (1998). |
| CYTH2 | ARNO is a guanine nucleotide exchange factor for ADP-ribosylation factor 6. Nature (2003).  Brefeldin A acts to stabilize an abortive ARF-GDP-Sec7 domain protein complex: involvement of specific residues of the Sec7 domain. Mol Cell (1999).  Structural snapshots of the mechanism and inhibition of a guanine nucleotide exchange factor. Nature (2003). |
| GGA1^1,2,3^, GGA2^3^, GGA3^3,4^ | ^1^The structure of the GGA1 - GAT domain reveals the molecular basis for ARF binding and membrane association of GGAs. Dev Cell (2003).  ^2^The trihelical bundle subdomain of the GGA proteins interacts with multiple partners through overlapping but distinct sites. J Biol Chem (2004).  ^3^GGAs: a family of ADP ribosylation factor-binding proteins related to adaptors and associated with the Golgi complex. J Cell Biol (2000).  ^4^The GGAs promote ARF - dependent recruitment of clathrin to the TGN.Cell (2001) |
| GOSR2 | Targeting of Arf-1 to the early Golgi by membrin, an ER-Golgi SNARE. J Cell Biol (2005). |
| INSR | ARF proteins mediate insulin - dependent activation of phospholipase D. Curr Biol (1997). |
| KDELR1 | KDEL-cargo regulates interactions between proteins involved in COPI vesicle traffic: measurements in living cells using FRET. Dev Cell (2001). |
| MDM4 | MdmX binding to ARF affects Mdm2 protein stability and p53 transactivation. J Biol Chem (2001). |
| NPM1 | Tumor suppressor ARF degrades B23, a nucleolar protein involved in ribosome biogenesis and cell proliferation. Mol Cell (2003).  Network organization of the human autophagy system. Nature (2010). |
| OPRM1 | ADP-ribosylation factor-dependent phospholipase D2 activation is required for agonist-induced mu-opioid receptor endocytosis. J Biol Chem (2003). |
| PIP5K1A | *Type I phosphatidylinositol 4-phosphate 5-kinase directly interacts with ADP-ribosylation factor 1 and is responsible for phosphatidylinositol 4,5-bisphosphate synthesis in the golgi compartment. J Biol Chem (2000). |
| PLD1 | Activation of phospholipase D1 by direct interaction with ADP-ribosylation factor 1 and RalA. FEBS Lett (1998). |
| PLD2 | Actin directly interacts with phospholipase D, inhibiting its activity. J Biol Chem (2001).  ADP-ribosylation factor-dependent phospholipase D2 activation is required for agonist-induced mu-opioid receptor endocytosis. J Biol Chem (2003).  Cardiac phospholipase D2 localizes to sarcolemmal membranes and is inhibited by alpha-actinin in an ADP-ribosylation factor-reversible manner. J Biol Chem (2000). |
| PLEKHA8 | FAPPs control Golgi-to-cell-surface membrane traffic by binding to ARF and PtdIns(4)P. Nat Cell Biol (2004). |
| PLIN2 | ADRP is dissociated from lipid droplets by ARF1 - dependent mechanism. Biochem Biophys Res Commun (2004). |
| RALA | Activation of phospholipase D1 by direct interaction with ADP-ribosylation factor 1 and RalA. FEBS Lett (1998).  Elevated phospholipase D activity in H - Ras- but not K-Ras - transformed cells by the synergistic action of RalA and ARF6. Mol Cell Biol (2003). |
| TMED10 | Recruitment to Golgi membranes of ADP-ribosylation factor 1 is mediated by the cytoplasmic domain of p23. EMBO J (2001).  Network organization of the human autophagy system. Nature (2010). |
| TMED2 | KDEL-cargo regulates interactions between proteins involved in COPI vesicle traffic: measurements in living cells using FRET. Dev Cell (2001). |
