# Supplementary material for: Topological and Functional Properties of the Small GTPases Protein Interaction Network
Source: PLoS One. 2012 Sep 13;7(9):e44882. doi: 10.1371/journal.pone.0044882 (PMC3441499; doi:10.1371/journal.pone.0044882)
Supplement: Table S2 — Proteins in the small GTPases network. (DOCX) [file pone.0044882.s003.docx]

| **Table S2. Proteins in the small GTPases network** | |
| --- | --- |
| **Gene symbol** | **Description** |
| ABI2 | abl-interactor 2; May act in regulation of cell growth and transformation by interacting with nonreceptor tyrosine kinases ABL1 and/or ABL2. May be involved in cytoskeletal reorganization. |
| ABL1 | c-abl oncogene 1, receptor tyrosine kinase; Regulates cytoskeleton remodeling during cell differentiation, cell division and cell adhesion. Localizes to dynamic actin structures, and phosphorylates CRK and CRKL, DOK1, and other proteins controlling cytoskeleton dynamics. |
| ABL2 | v-abl Abelson murine leukemia viral oncogene homolog 2 (arg, Abelson-related gene); Regulates cytoskeleton remodeling during cell differentiation, cell division and cell adhesion. |
| ACTA1 | actin, alpha 1, skeletal muscle; Actins are highly conserved proteins that are involved in various types of cell motility and are ubiquitously expressed in all eukaryotic cells (By similarity) |
| ACTB | actin, beta; Actins are highly conserved proteins that are involved in various types of cell motility and are ubiquitously expressed in all eukaryotic cells (By similarity) |
| ACTR3 | ARP3 actin-related protein 3 homolog (yeast); Plays a role in the organization of the actin cytoskeleton. May function as ATP-binding component of the Arp2/3 complex which is involved in regulation of actin polymerization and together with an activating nucleation-promoting factor (NPF) mediates the formation of branched actin networks. May decrease the metastatic potential of tumors |
| AGER | advanced glycosylation end product-specific receptor; Mediates interactions of advanced glycosylation end products (AGE). These are nonenzymatically glycosylated proteins which accumulate in vascular tissue in aging and at an accelerated rate in diabetes. Receptor for amyloid beta peptide |
| AGTR1 | angiotensin II receptor, type 1; Receptor for angiotensin II. Mediates its action by association with G proteins that activate a phosphatidylinositol- calcium second messenger system |
| AHSG | alpha-2-HS-glycoprotein; Promotes endocytosis, possesses opsonic properties and influences the mineral phase of bone. Shows affinity for calcium and barium ions |
| AKAP13 | A kinase (PRKA) anchor protein 13; Anchors cAMP-dependent protein kinase (PKA) and acts as an adapter protein to selectively couple G alpha-13 and Rho. |
| AKT1 | v-akt murine thymoma viral oncogene homolog 1; General protein kinase capable of phosphorylating several known proteins. Phosphorylates TBC1D4. Signals downstream of phosphatidylinositol 3-kinase (PI(3)K) to mediate the effects of various growth factors such as platelet-derived growth factor (PDGF), epidermal growth factor (EGF), insulin and insulin-like growth factor I (IGF-I). |
| AKT2 | v-akt murine thymoma viral oncogene homolog 2; General protein kinase capable of phosphorylating several known proteins |
| ALK | anaplastic lymphoma receptor tyrosine kinase; Orphan receptor with a tyrosine-protein kinase activity. Appears to play an important role in the normal development and function of the nervous system. |
| ALS2CL | ALS2 C-terminal like; Acts as a guanine nucleotide exchange factor (GEF) for Rab5 GTPase. |
| ANKFY1 | ankyrin repeat and FYVE domain containing 1 |
| ANKRD11 | ankyrin repeat domain 11; May recruit HDACs to the p160 coactivators/nuclear receptor complex to inhibit ligand-dependent transactivation |
| ANXA2 | annexin A2 pseudogene 1; Calcium-regulated membrane-binding protein whose affinity for calcium is greatly enhanced by anionic phospholipids (By similarity). |
| AP1B1 | adaptor-related protein complex 1, beta 1 subunit; Subunit of clathrin-associated adaptor protein complex 1 that plays a role in protein sorting in the late-Golgi/trans-Golgi network (TGN) and/or endosomes. |
| AP1G1 | adaptor-related protein complex 1, gamma 1 subunit; Subunit of clathrin-associated adaptor protein complex 1 that plays a role in protein sorting in the late-Golgi/trans-Golgi network (TGN) and/or endosomes. |
| AP1G2 | adaptor-related protein complex 1, gamma 1 subunit; Subunit of clathrin-associated adaptor protein complex 1 that plays a role in protein sorting in the late-Golgi/trans-Golgi network (TGN) and/or endosomes. |
| AP3D1 | adaptor-related protein complex 3, delta 1 subunit; Part of the AP-3 complex, an adaptor-related complex which is not clathrin-associated. The complex is associated with the Golgi region as well as more peripheral structures. |
| AP3S2 | adaptor-related protein complex 3, sigma 2 subunit; Part of the AP-3 complex, an |
| AP4E1 | adaptor-related protein complex 4, epsilon 1 subunit; Subunit of novel type of clathrin- or non-clathrin- associated protein coat involved in targeting proteins from the trans-Golgi network (TGN) to the endosomal-lysosomal system |
| AP4M1 | adaptor-related protein complex 4, mu 1 subunit; Subunit of novel type of clathrin- or non-clathrin- associated protein coat involved in targeting proteins from the trans-Golgi network (TGN) to the endosomal-lysosomal system |
| APBB1IP | amyloid beta (A4) precursor protein-binding, family B, member 1 interacting protein; Appears to function in the signal transduction from Ras activation to actin cytoskeletal remodeling. |
| APPL1 | adaptor protein, phosphotyrosine interaction, PH domain and leucine zipper containing 1; Required for the regulation of cell proliferation in response to extracellular signals from an early endosomal compartment. Links Rab5 to nuclear signal transduction |
| APPL2 | adaptor protein, phosphotyrosine interaction, PH domain and leucine zipper containing 2; Required for the regulation of cell proliferation in response to extracellular signals mediated by an early endosomal compartment. Links Rab5 to nuclear signal transduction |
| AR | androgen receptor; Steroid hormone receptors are ligand-activated transcription factors that regulate eukaryotic gene expression and affect cellular proliferation and differentiation in target tissues. |
| ARAF | v-raf murine sarcoma 3611 viral oncogene homolog; Involved in the transduction of mitogenic signals from the cell membrane to the nucleus |
| ARF1 | ADP-ribosylation factor 1; GTP-binding protein that functions as an allosteric activator of the cholera toxin catalytic subunit, an ADP- ribosyltransferase. Involved in protein trafficking among different compartments. Modulates vesicle budding and uncoating within the Golgi complex. |
| ARF3 | ADP-ribosylation factor 3; GTP-binding protein that functions as an allosteric activator of the cholera toxin catalytic subunit, an ADP- ribosyltransferase. Involved in protein trafficking among different compartments. |
| ARF4 | ADP-ribosylation factor 4; GTP-binding protein that functions as an allosteric activator of the cholera toxin catalytic subunit, an ADP- ribosyltransferase. Involved in protein trafficking; may modulate vesicle budding and uncoating within the Golgi apparatus |
| ARF5 | ADP-ribosylation factor 5; GTP-binding protein that functions as an allosteric activator of the cholera toxin catalytic subunit, an ADP- ribosyltransferase. Involved in protein trafficking; may modulate vesicle budding and uncoating within the Golgi apparatus" |
| ARF6 | ADP-ribosylation factor 6; GTP-binding protein that functions as an allosteric activator of the cholera toxin catalytic subunit, an ADP- ribosyltransferase. Involved in protein trafficking; may modulate vesicle budding and uncoating within the Golgi apparatus" |
| ARFGAP1 | ADP-ribosylation factor GTPase activating protein 1; GTPase-activating protein (GAP) for the ADP ribosylation factor 1 (ARF1). |
| ARFIP1 | ADP-ribosylation factor interacting protein 1; Putative target protein of ADP-ribosylation factor |
| ARFIP2 | ADP-ribosylation factor interacting protein 2; Putative target protein of ADP-ribosylation factor. Involved in membrane ruffling |
| ARFRP1 | ADP-ribosylation factor related protein 1; Possibly involved in plasma membrane-related signaling events |
| ARHGAP1 | Rho GTPase activating protein 1; GTPase activator for the Rho, Rac and Cdc42 proteins |
| ARHGAP10 | Rho GTPase activating protein 10; GTPase activator for the small GTPases RhoA and Cdc42 by converting them to an inactive GDP-bound state. |
| ARHGAP15 | Rho GTPase activating protein 15; GTPase activator for the Rho-type GTPases by converting them to an inactive GDP-bound state. Has activity toward RAC1. Overexpression results in an increase in actin stress fibers and cell contraction |
| ARHGAP17 | Rho GTPase activating protein 17; Rho GTPase-activating protein involved in the maintenance of tight junction by regulating the activity of CDC42, thereby playing a central role in apical polarity of epithelial cells. |
| ARHGAP21 | Rho GTPase activating protein 21; Functions as a GTPase-activating protein (GAP) for RHOA and CDC42. Downstream partner of ARF1 which may control Golgi apparatus structure and function. Also required for CTNNA1 recruitment to adherens junctions |
| ARHGAP24 | Rho GTPase activating protein 24; Rho GTPase-activating protein involved in cell polarity, cell morphology and cytoskeletal organization. |
| ARHGAP26 | Rho GTPase activating protein 26; GTPase-activating protein for RHOA and CDC42 |
| ARHGAP29 | Rho GTPase activating protein 29; GTPase activator for the Rho-type GTPases by converting them to an inactive GDP-bound state. Has strong activity toward RHOA, and weaker activity toward RAC1 and CDC42. |
| ARHGAP31 | Cdc42 GTPase-activating protein ; Functions as a GTPase-activating protein (GAP) for RAC1 and CDC42. Required for cell spreading, polarized lamellipodia formation and cell migration |
| ARHGAP32 | Rho/Cdc42/Rac GTPase-activating protein RICS GTPase-activating protein (GAP) promoting GTP hydrolysis on RHOA, CDC42 and RAC1 small GTPases. May be involved in the differentiation of neuronal cells during the formation of neurite extensions. |
| ARHGAP33 | sorting nexin 26; May be involved in several stages of intracellular trafficking. Could play an important role in the regulation of glucose transport by insulin. |
| ARHGAP4 | Inhibitory effect on stress fiber organization. May down-regulate Rho-like GTPase in hematopoietic cells |
| ARHGAP44 | Rho GTPase-activating protein RICH2 |
| ARHGAP5 | Rho GTPase activating protein 5; GTPase-activating protein for Rho family members |
| ARHGDIA | Rho GDP dissociation inhibitor (GDI) alpha |
| ARHGDIB | Rho GDP dissociation inhibitor (GDI) beta |
| ARHGDIG | Rho GDP dissociation inhibitor (GDI) gamma |
| ARHGEF1 | Rho guanine nucleotide exchange factor (GEF) 1; Seems to play a role in the regulation of RhoA GTPase by guanine nucleotide-binding alpha-12 (GNA12) and alpha-13 (GNA13) subunits. Acts as GTPase-activating protein (GAP) for GNA12 and GNA13, and as guanine nucleotide exchange factor (GEF) for RhoA GTPase. |
| ARHGEF11 | Rho guanine nucleotide exchange factor |
| ARHGEF12 | Rho guanine nucleotide exchange factor |
| ARHGEF18 | Rho/Rac guanine nucleotide exchange factor |
| ARHGEF19 | Rho guanine nucleotide exchange factor |
| ARHGEF2 | Rho/Rac guanine nucleotide exchange factor (GEF) 2; Activates Rho-GTPases by promoting the exchange of GDP for GTP. May be involved in epithelial barrier permeability, cell motility and polarization, dendritic spine morphology, antigen presentation, leukemic cell differentiation, cell cycle regulation, and cancer. |
| ARHGEF25 | Guanine nucleotide exchange factor GEF |
| ARHGEF3 | guanine nucleotide exchange factor |
| ARHGEF4 | Rho guanine nucleotide exchange factor |
| ARHGEF5 | Rho guanine nucleotide exchange factor |
| ARHGEF6 | Rac/Cdc42 guanine nucleotide exchange factor (GEF) 6; Acts as a RAC1 guanine nucleotide exchange factor |
| ARHGEF7 | Rho guanine nucleotide exchange factor |
| ARL1 | ADP-ribosylation factor-like 1; GTP-binding protein that has very low efficiency as allosteric activator of the cholera toxin catalytic subunit, an ADP-ribosyltransferase. |
| ARL11 | ADP-ribosylation factor-like 11; May play a role in apoptosis. May act as a tumor suppressor |
| ARL16 | ADP-ribosylation factor-like 16 |
| ARL2 | ADP-ribosylation factor-like 2; GTP-binding protein that does not act as an allosteric activator of the cholera toxin catalytic subunit. |
| ARL2BP | ADP-ribosylation factor-like 2 binding protein; May play a role as an effector of the ADP-ribosylation factor-like protein 2, ARL2 |
| ARL3 | ADP-ribosylation factor-like 2 binding protein; May play a role as an effector of the ADP-ribosylation factor-like protein 2, ARL2 |
| ARL4A | ADP-ribosylation factor-like 4A; Does not act as an allosteric activator of the cholera toxin catalytic subunit (By similarity) |
| ARL5A | ADP-ribosylation factor-like 5A; Lacks ADP-ribosylation enhancing activity |
| ARL6 | ADP-ribosylation factor-like 6 |
| ARL6IP1 | ADP-ribosylation factor-like 6 interacting protein 1; May be involved in protein transport, membrane trafficking, or cell signaling during hematopoietic maturation |
| ARL6IP4 | ADP-ribosylation-like factor 6 interacting protein 4; In case of infection by Herpes simplex virus (HSVI), may act as a splicing inhibitor of HSVI pre-mRNA |
| ARL6IP5 | ADP-ribosylation-like factor 6 interacting protein 5; Regulates intracellular concentrations of taurine and glutamate. |
| ARL6IP6 | ADP-ribosylation-like factor 6 interacting protein 6 |
| ARRB1 | arrestin, beta 1; Functions in regulating agonist-mediated G-protein coupled receptor (GPCR) signaling by mediating both receptor desensitization and resensitization processes. |
| ARRB2 | Functions in regulating agonist-mediated G-protein coupled receptor (GPCR) signaling by mediating both receptor desensitization and resensitization processes. |
| ASAP1 | Functions in regulating agonist-mediated G-protein coupled receptor (GPCR) signaling by mediating both receptor desensitization and resensitization processes. |
| ASAP2 | ArfGAP with SH3 domain, ankyrin repeat and PH domain 2; Activates the small GTPases ARF1, ARF5 and ARF6. Regulates the formation of post-Golgi vesicles and modulates constitutive secretion. |
| ASAP2 | ArfGAP with SH3 domain, ankyrin repeat and PH domain 2; Activates the small GTPases ARF1, ARF5 and ARF6. Regulates the formation of post-Golgi vesicles and modulates constitutive secretion. |
| ASAP3 | ArfGAP with SH3 domain, ankyrin repeat and PH domain 3; Promotes cell proliferation |
| ATL2 | atlastin GTPase 2; GTPase tethering membranes through formation of trans- homooligomer and mediating homotypic fusion of endoplasmic reticulum membranes. |
| ATP6V0C | ATPase, H+ transporting, lysosomal 16kDa, V0 subunit c; Proton-conducting pore forming subunit of the membrane integral V0 complex of vacuolar ATPase. |
| ATP6V1C1 | ATPase, H+ transporting, lysosomal 42kDa, V1 subunit C1; Subunit of the peripheral V1 complex of vacuolar ATPase. |
| BAIAP2 | Adapter protein that links membrane-bound small G- proteins to cytoplasmic effector proteins. Necessary for CDC42- mediated reorganization of the actin cytoskeleton and for RAC1- mediated membrane ruffling. |
| BBS1 | Bardet-Biedl syndrome 1; The BBSome complex is required for ciliogenesis but is dispensable for centriolar satellite function. This ciliogenic function is mediated in part by the Rab8 GDP/GTP exchange factor, which localizes to the basal body and contacts the BBSome. Rab8(GTP) enters the primary cilium and promotes extension of the ciliary membrane. |
| BCL2 | B-cell CLL/lymphoma 2; Suppresses apoptosis in a variety of cell systems including factor-dependent lymphohematopoietic and neural cells. Regulates cell death by controlling the mitochondrial membrane permeability. |
| BCL3 | B-cell CLL/lymphoma 3; Could be a transcriptional activating factor. Functions as a form of I-kappa-B specific for NF-kappa-B p50 subunit inhibiting its translocation to the nucleus |
| BCR | breakpoint cluster region; GTPase-activating protein for RAC1 and CDC42. |
| BICD1 | bicaudal D homolog 1 (Drosophila); Regulates coat complex coatomer protein I (COPI)- independent Golgi-endoplasmic reticulum transport by recruiting the dynein-dynactin motor complex |
| BICD2 | bicaudal D homolog 2 (Drosophila); May play a role in the dynein-dynactin interactions on the surface of membranous organelles, by associating with these complexes. Regulates coat complex coatomer protein I (COPI)- independent Golgi-endoplasmic reticulum transport by recruiting the dynein-dynactin motor complex |
| BID | BH3 interacting domain death agonist; The major proteolytic product p15 BID allows the release of cytochrome c (By similarity). |
| BIN1 | bridging integrator 1; May be involved in regulation of synaptic vesicle endocytosis. May act as a tumor suppressor and inhibits malignant cell transformation |
| BIRC6 | baculoviral IAP repeat-containing 6; May protect cells from undergoing apoptosis |
| BIW | bellwether; Mitochondrial membrane ATP synthase (F(1)F(0) ATP synthase or Complex V) produces ATP from ADP in the presence of a proton gradient across the membrane which is generated by electron transport complexes of the respiratory chain. |
| BLZF1 | basic leucine zipper nuclear factor 1; Required for normal Golgi structure and for protein transport from the endoplasmic reticulum (ER) through the Golgi apparatus to the cell surface |
| BMX | BMX non-receptor tyrosine kinase; Activity is required for interleukin 6 (IL-6) induced differentiation. May play a role in the growth and differentiation of hematopoietic cells. May be involved in signal transduction in endocardial and arterial endothelial cells |
| BNIP2 | BCL2/adenovirus E1B 19kDa interacting protein 2; Implicated in the suppression of cell death. |
| BNIPL | BCL2/adenovirus E1B 19kD interacting protein like; May be a bridge molecule between BCL2 and ARHGAP1/CDC42 in promoting cell death |
| BPGAP1 | Rho GTPase activating protein 8; GTPase activator for the Rho-type GTPases by converting them to an inactive GDP-bound state (By similarity) |
| BRAF | v-raf murine sarcoma viral oncogene homolog B1; Involved in the transduction of mitogenic signals from the cell membrane to the nucleus. |
| BRAP | BRCA1 associated protein; Negatively regulates MAP kinase activation by limiting the formation of Raf/MEK complexes probably by inactivation of the KSR1 scaffold protein. |
| BSN | bassoon (presynaptic cytomatrix protein); Is thought to be involved in the organization of the cytomatrix at the nerve terminals active zone (CAZ) which regulates neurotransmitter release. |
| BSND | Bartter syndrome, infantile, with sensorineural deafness (Barttin); Functions as a beta-subunit for CLCNKA and CLCNKB chloride channels. In the kidney CLCNK/BSND heteromers mediate chloride reabsorption by facilitating its basolateral efflux. |
| BTF3 | basic transcription factor 3; General transcription factor. BTF3 can form a stable complex with RNA polymerase II. Required for the initiation of transcription |
| C19orf62 | BRCA1-A complex subunit MERIT40 (Mediator of RAP80 interactions and targeting subunit of 40 kDa)(New component of the BRCA1-A complex); Component of the BRCA1-A complex, a complex that specifically recognizes 'Lys-63'-linked ubiquitinated histones H2A and H2AX at DNA lesions sites, leading to target the BRCA1-BARD1 heterodimer to sites of DNA damage at double-strand breaks (DSBs). |
| C2 | complement component 2 |
| CACNB1 | calcium channel, voltage-dependent, beta 1 subunit; The beta subunit of voltage-dependent calcium channels contributes to the function of the calcium channel by increasing peak calcium current, shifting the voltage dependencies of activation and inactivation, modulating G protein inhibition and controlling the alpha-1 subunit membrane targeting |
| CACNB2 | calcium channel, voltage-dependent, beta 2 subunit; The beta subunit of voltage-dependent calcium channels contributes to the function of the calcium channel by increasing peak calcium current, shifting the voltage dependencies of activation and inactivation, modulating G protein inhibition and controlling the alpha-1 subunit membrane targeting |
| CACNB4 | calcium channel, voltage-dependent, beta 4 subunit; The beta subunit of voltage-dependent calcium channels contributes to the function of the calcium channel by increasing peak calcium current, shifting the voltage dependencies of activation and inactivation, modulating G protein inhibition and controlling the alpha-1 subunit membrane targeting |
| CALM1 | calmodulin 1 (phosphorylase kinase, delta); Calmodulin mediates the control of a large number of enzymes and other proteins by Ca(2+). Among the enzymes to be stimulated by the calmodulin-Ca(2+) complex are a number of protein kinases and phosphatases. |
| CAMK2G | calcium/calmodulin-dependent protein kinase II gamma; CaM-kinase II (CAMK2) is a prominent kinase in the central nervous system that may function in long-term potentiation and neurotransmitter release |
| CAPN1 | calpain 1, (mu/I) large subunit; Calcium-regulated non-lysosomal thiol-protease which catalyze limited proteolysis of substrates involved in cytoskeletal remodeling and signal transduction |
| CAPON | nitric oxide synthase 1 (neuronal) adaptor protein; Adapter protein involved in neuronal nitric-oxide (NO) synthesis regulation via its association with nNOS/NOS1. |
| CASK | calcium/calmodulin-dependent serine protein kinase (MAGUK family); Multidomain scaffolding protein with a role in synaptic transmembrane protein anchoring and ion channel trafficking. |
| CASP10 | caspase 10, apoptosis-related cysteine peptidase; Involved in the activation cascade of caspases responsible for apoptosis execution. |
| CASP3 | caspase 3, apoptosis-related cysteine peptidase; Involved in the activation cascade of caspases responsible for apoptosis execution. |
| CASP7 | caspase 7, apoptosis-related cysteine peptidase; Involved in the activation cascade of caspases responsible for apoptosis execution. Cleaves and activates sterol regulatory element binding proteins (SREBPs). |
| CASP8 | caspase 8, apoptosis-related cysteine peptidase; Most upstream protease of the activation cascade of caspases responsible for the TNFRSF6/FAS mediated and TNFRSF1A induced cell death. |
| CAV1 | caveolin 1, caveolae protein, 22kDa; May act as a scaffolding protein within caveolar membranes. Interacts directly with G-protein alpha subunits and can functionally regulate their activity |
| CBX1 | chromobox homolog 1 (HP1 beta homolog Drosophila ); Component of heterochromatin. Recognizes and binds histone H3 tails methylated at 'Lys-9', leading to epigenetic repression. |
| CBX3 | chromobox homolog 3 (HP1 gamma homolog, Drosophila); Seems to be involved in transcriptional silencing in heterochromatin-like complexes. |
| CBX5 | chromobox homolog 5 (HP1 alpha homolog, Drosophila); Component of heterochromatin. |
| CCDC104 | coiled-coil domain containing 104 |
| CD2AP | CD2-associated protein; Seems to act as an adapter protein between membrane proteins and the actin cytoskeleton. May play a role in receptor clustering and cytoskeletal polarity in the junction between T- cell and antigen-presenting cell. |
| CDC25A | cell division cycle 25 homolog A (S. pombe); Tyrosine protein phosphatase which functions as a dosage-dependent inducer of mitotic progression. |
| CDC25B | cell division cycle 25 homolog B (S. pombe); Tyrosine protein phosphatase which functions as a dosage-dependent inducer of mitotic progression. |
| CDC25C | cell division cycle 25 homolog C (S. pombe); Functions as a dosage-dependent inducer in mitotic control. It is a tyrosine protein phosphatase required for progression of the cell cycle. |
| CDC42 | cell division cycle 42 (GTP binding protein, 25kDa); Plasma membrane-associated small GTPase which cycles between an active GTP-bound and an inactive GDP-bound state. |
| CDC42BPA | CDC42 binding protein kinase alpha (DMPK-like); May act as a downstream effector of CDC42 in cytoskeletal reorganization. |
| CDC42BPG | CDC42 binding protein kinase gamma (DMPK-like); May act as a downstream effector of CDC42 in cytoskeletal reorganization. |
| CDC42EP1 | CDC42 effector protein (Rho GTPase binding) 1; Probably involved in the organization of the actin cytoskeleton. Induced membrane extensions in fibroblasts |
| CDC42EP2 | effector protein (Rho GTPase binding) 2; Probably involved in the organization of the actin cytoskeleton. |
| CDC42EP3 | CDC42 effector protein (Rho GTPase binding) 3; Probably involved in the organization of the actin cytoskeleton. |
| CDC42EP4 | CDC42 effector protein (Rho GTPase binding) 4; Probably involved in the organization of the actin cytoskeleton. |
| CDC42EP5 | CDC42 effector protein (Rho GTPase binding) 5; Probably involved in the organization of the actin cytoskeleton. |
| CDC42SE1 | CDC42 small effector 1; Probably involved in the organization of the actin cytoskeleton by acting downstream of CDC42, inducing actin filament assembly. |
| CDC42SE2 | CDC42 small effector 2; Probably involved in the organization of the actin cytoskeleton by acting downstream of CDC42, inducing actin filament assembly. |
| CDH1 | cadherin 1, type 1, E-cadherin (epithelial); Cadherins are calcium-dependent cell adhesion proteins. |
| CDH2 | cadherin 2, type 1, N-cadherin (neuronal); Cadherins are calcium dependent cell adhesion proteins. |
| CDK1 | cyclin-dependent kinase 1; Plays a key role in the control of the eukaryotic cell cycle. |
| CDKN1B | cyclin-dependent kinase inhibitor 1B (p27, Kip1); Important regulator of cell cycle progression. Involved in G1 arrest. |
| CHM | choroideremia (Rab escort protein 1); Binds unprenylated Rab proteins, presents it to the catalytic Rab GGTase dimer, and remains bound to it after the geranylgeranyl transfer reaction. |
| CHML | choroideremia-like (Rab escort protein 2); Binds unprenylated Rab proteins, presents it to the catalytic Rab GGTase dimer, and remains bound to it after the geranylgeranyl transfer reaction. |
| CHMP6 | chromatin modifying protein 6; Probable core component of the endosomal sorting required for transport complex III (ESCRT-III) which is involved in multivesicular bodies (MVBs) formation and sorting of endosomal cargo proteins into MVBs. |
| CHN1 | chimerin (chimaerin) 1; GTPase-activating protein for p21-rac and a phorbol ester receptor. |
| CHN2 | chimerin (chimaerin) 2; GTPase-activating protein for p21-rac. |
| CHRM3 | cholinergic receptor, muscarinic 3; The muscarinic acetylcholine receptor mediates various cellular responses, including inhibition of adenylate cyclase, breakdown of phosphoinositides and modulation of potassium channels through the action of G proteins. |
| CIB1 | calcium and integrin binding 1 (calmyrin); May convert the inactive conformation of integrin alpha- IIb/beta3 to an active form through binding to the integrin cytoplasmic domain. |
| CIT | citron (rho-interacting, serine/threonine kinase 21); Required for KIF14 localization to the central spindle and midbody. |
| CLIP1 | CAP-GLY domain containing linker protein 1; Seems to be a intermediate filament associated protein that links endocytic vesicles to microtubules |
| CNKSR1 | connector enhancer of kinase suppressor of Ras 1; May function as an adapter protein or regulator of Ras signaling pathways |
| CNKSR2 | connector enhancer of kinase suppressor of Ras 2; May function as an adapter protein or regulator of Ras signaling pathways |
| CNTNAP1 | contactin associated protein 1; Seems to play a role in the formation of functional distinct domains critical for saltatory conduction of nerve impulses in myelinated nerve fibers. |
| COPB1 | coatomer protein complex, subunit beta 1; The coatomer is a cytosolic protein complex that binds to dilysine motifs and reversibly associates with Golgi non- clathrin-coated vesicles, which further mediate biosynthetic protein transport from the ER, via the Golgi up to the trans Golgi network. |
| COPE | coatomer protein complex, subunit epsilon; The coatomer is a cytosolic protein complex that binds to dilysine motifs and reversibly associates with Golgi non- clathrin-coated vesicles, which further mediate biosynthetic protein transport from the ER, via the Golgi up to the trans Golgi network. |
| COPG | coatomer protein complex, subunit gamma; The coatomer is a cytosolic protein complex that binds to dilysine motifs and reversibly associates with Golgi non- clathrin-coated vesicles, which further mediate biosynthetic protein transport from the ER, via the Golgi up to the trans Golgi network. |
| COPG2 | coatomer protein complex, subunit gamma 2; The coatomer is a cytosolic protein complex that binds to dilysine motifs and reversibly associates with Golgi non- clathrin-coated vesicles, which further mediate biosynthetic protein transport from the ER, via the Golgi up to the trans Golgi network. |
| CREB3L2 | cAMP responsive element binding protein 3-like 2 |
| CRMP1 | collapsin response mediator protein 1; Necessary for signaling by class 3 semaphorins and subsequent remodeling of the cytoskeleton. |
| CSDA | cold shock domain protein A pseudogene 1; Binds to the GM-CSF promoter. Seems to act as a repressor. Binds also to full length mRNA and to short RNA sequences containing the consensus site 5'-UCCAUCA-3'. |
| CSE1L | CSE1 chromosome segregation 1-like (yeast); Export receptor for importin-alpha. Mediates importin- alpha re-export from the nucleus to the cytoplasm after import substrates (cargos) have been released into the nucleoplasm. |
| CSNK2A1 | casein kinase 2, alpha 1 polypeptide pseudogene; Casein kinases are operationally defined by their preferential utilization of acidic proteins such as caseins as substrates. The alpha and alpha' chains contain the catalytic site. |
| CSPG4 | chondroitin sulfate proteoglycan 4; Proteoglycan playing a role in cell proliferation and migration which stimulates endothelial cells motility during microvascular morphogenesis. |
| CTNNB1 | catenin (cadherin-associated protein), beta 1, 88kDa; Involved in the regulation of cell adhesion and in signal transduction through the Wnt pathway |
| CUL1 | cullin 1; Core component of multiple cullin-RING-based SCF (SKP1- CUL1-F-box protein) E3 ubiquitin-protein ligase complexes, which mediate the ubiquitination of proteins involved in cell cycle progression, signal transduction and transcription. In the SCF complex, serves as a rigid scaffold that organizes the SKP1-F-box protein and RBX1 subunits. |
| CYBA1 | cytochrome b-245, alpha polypeptide; Critical component of the membrane-bound oxidase of phagocytes that generates superoxide. |
| CYBB | cytochrome b-245, beta polypeptide; Critical component of the membrane-bound oxidase of phagocytes that generates superoxide. It is the terminal component of a respiratory chain that transfers single electrons from cytoplasmic NADPH across the plasma membrane to molecular oxygen on the exterior. |
| CYFIP1 | cytoplasmic FMR1 interacting protein 1; Involved in formation of membrane ruffles and lamellipodia protrusions and in axon outgrowth. Binds to F-actin but not to RNA |
| CYTH1 | cytohesin 1; Promotes guanine-nucleotide exchange on ARF1 and ARF5. Promotes the activation of ARF through replacement of GDP with GTP |
| CYTH2 | Promotes guanine-nucleotide exchange on ARF1, ARF3 and ARF6. Promotes the activation of ARF through replacement of GDP with GTP |
| D2HGDH | D-2-hydroxyglutarate dehydrogenase; Catalyzes the oxidation of D-2-hydroxyglutarate to alpha-ketoglutarate |
| DAAM1 | dishevelled associated activator of morphogenesis 1; Binds to disheveled (Dvl) and Rho, and mediates Wnt- induced Dvl-Rho complex formation. |
| DCTN1 | dynactin 1 (p150, glued homolog, Drosophila); Required for the cytoplasmic dynein-driven retrograde movement of vesicles and organelles along microtubules. Dynein- dynactin interaction is a key component of the mechanism of axonal transport of vesicles and organelles |
| DEF6 | differentially expressed in FDCP 6 homolog (mouse); Phosphatidylinositol 3,4,5-trisphosphate-dependent guanine nucleotide exchange factor (GEF) which plays a role in the activation of Rho GTPases RAC1, RhoA and CDC42. |
| DGKQ | diacylglycerol kinase, theta 110kDa; Phosphorylates diacylglycerol (DAG) to generate phosphatidic acid (PA). |
| DGKZ | diacylglycerol kinase, zeta 104kDa; Displays a strong preference for 1,2-diacylglycerols over 1,3-diacylglycerols, but lacks substrate specificity among molecular species of long chain diacylglycerols. |
| DIAPH1 | diaphanous homolog 1 (Drosophila); Acts in a Rho-dependent manner to recruit PFY1 to the membrane. Required for the assembly of F-actin structures, such as actin cables and stress fibers. |
| DIAPH2 | diaphanous homolog 2 (Drosophila); Could be involved in oogenesis. |
| DIRAS2 | DIRAS family, GTP-binding RAS-like 2; Displays low GTPase activity and exist predominantly in the GTP-bound form |
| DMPK | dystrophia myotonica-protein kinase; Critical to the modulation of cardiac contractility and to the maintenance of proper cardiac conduction activity. |
| DMXL2 | Dmx-like 2; May serve as a scaffold protein for MADD and RAB3GA on synaptic vesicles |
| DOCK1 | dedicator of cytokinesis 1; Involved in cytoskeletal rearrangements required for phagocytosis of apoptotic cells and cell motility. |
| DOCK10 | dedicator of cytokinesis 10; Potential guanine nucleotide exchange factor (GEF). |
| DOCK7 | dedicator of cytokinesis 7; Functions as a guanine nucleotide exchange factor (GEF) |
| DOCK8 | dedicator of cytokinesis 8; Potential guanine nucleotide exchange factor (GEF). |
| DOCK9 | dedicator of cytokinesis 9; Guanine nucleotide-exchange factor (GEF) that activates CDC42 by exchanging bound GDP for free GTP. |
| DPYSL2 | dihydropyrimidinase-like 2; Necessary for signaling by class 3 semaphorins and subsequent remodeling of the cytoskeleton. |
| DSTYK | dual serine/threonine and tyrosine protein kinase; May induce both caspase-dependent apoptosis and caspase- independent cell death |
| DTNBP1 | dystrobrevin binding protein 1; Plays a role in the biogenesis of lysosome-related organelles such as platelet dense granule and melanosomes |
| DUT | deoxyuridine triphosphatase; This enzyme is involved in nucleotide metabolism: it produces dUMP, the immediate precursor of thymidine nucleotides and it decreases the intracellular concentration of dUTP so that uracil cannot be incorporated into DNA |
| DVL1 | dishevelled, dsh homolog 1 (Drosophila); May play a role in the signal transduction pathway mediated by multiple Wnt genes |
| DVL2 | dishevelled, dsh homolog 2 (Drosophila); May play a role in the signal transduction pathway mediated by multiple Wnt genes |
| DYNC1LI1 | dynein, cytoplasmic 1, light intermediate chain 1; May play a role in binding dynein to membranous organelles or chromosomes. |
| DYNLL1 | dynein, light chain, LC8-type 1; May be involved in some aspects of dynein-related intracellular transport and motility. |
| EEA1 | early endosome antigen 1; Binds phospholipid vesicles containing phosphatidylinositol 3-phosphate and participates in endosomal trafficking |
| EGFR | epidermal growth factor receptor (erythroblastic leukemia viral (v-erb-b) oncogene homolog, avian); Receptor for EGF, but also for other members of the EGF family, as TGF-alpha, amphiregulin, betacellulin, heparin-binding EGF-like growth factor, GP30 and vaccinia virus growth factor. |
| EIF1B | eukaryotic translation initiation factor 1B; Necessary for scanning and involved in initiation site selection. Promotes the assembly of 48S ribosomal complexes at the authentic initiation codon of a conventional capped mRNA |
| EIF2AK2 | eukaryotic translation initiation factor 2-alpha kinase 2; Following activation by double-stranded RNA in the presence of ATP, the kinase becomes autophosphorylated and can catalyze the phosphorylation of the translation initiation factor EIF2S1, which leads to an inhibition of the initiation of protein synthesis. |
| EIF2C4 | eukaryotic translation initiation factor 2C, 4; Required for RNA-mediated gene silencing (RNAi). Binds to short RNAs such as microRNAs (miRNAs) and represses the translation of mRNAs which are complementary to them. |
| EIF6 | eukaryotic translation initiation factor 6; Binds to the 60S ribosomal subunit and prevents its association with the 40S ribosomal subunit to form the 80S initiation complex |
| ELMO1 | engulfment and cell motility 1; Involved in cytoskeletal rearrangements required for phagocytosis of apoptotic cells and cell motility. |
| ELMO3 | engulfment and cell motility 3; Involved in cytoskeletal rearrangements required for phagocytosis of apoptotic cells and cell motility. |
| ENSG00000206525 olfactory receptor 2W1 | |
| ENSG00000237441 Ral guanine nucleotide dissociation stimulator-like 2 | |
| EP300 | microRNA 1281; Functions as histone acetyltransferase and regulates transcription via chromatin remodeling. |
| EPB42 | Probably plays an important role in the regulation of erythrocyte shape and mechanical properties |
| EPHB2 | EPH receptor B2; Receptor for members of the ephrin-B family. Acts as a tumor suppressor |
| ERAS | ES cell expressed Ras; Ras proteins bind GDP/GTP and possess intrinsic GTPase activity. Plays an important role in the tumor-like growth properties of embryonic stem cells (By similarity) |
| ERBB2IP | erbb2 interacting protein; Acts as an adapter for the receptor ERBB2, in epithelia. |
| ERC1 | ELKS/RAB6-interacting/CAST family member 1; Regulatory subunit of the IKK complex. |
| ERRFI1 | ERBB receptor feedback inhibitor 1 |
| EXOC1 | exocyst complex component 1; Component of the exocyst complex involved in the docking of exocystic vesicles with fusion sites on the plasma membrane |
| EXOC2 | exocyst complex component 2; Component of the exocyst complex involved in the docking of exocystic vesicles with fusion sites on the plasma membrane |
| EXOC3 | exocyst complex component 3; Component of the exocyst complex involved in the docking of exocystic vesicles with fusion sites on the plasma membrane |
| EXOC4 | exocyst complex component 4; Component of the exocyst complex involved in the docking of exocystic vesicles with fusion sites on the plasma membrane (By similarity) |
| EXOC5 | exocyst complex component 5; Component of the exocyst complex involved in the docking of exocystic vesicles with fusion sites on the plasma membrane |
| EXOC6B | exocyst complex component 6B; Component of the exocyst complex involved in the docking of exocystic vesicles with fusion sites on the plasma membrane |
| EXOC7 | exocyst complex component 7; Component of the exocyst complex involved in the docking of exocystic vesicles with fusion sites on the plasma membrane |
| EXOC8 | exocyst complex component 8; Component of the exocyst complex involved in the docking of exocystic vesicles with fusion sites on the plasma membrane |
| EXPH5 | exophilin 5; May act as Rab effector protein and play a role in vesicle trafficking |
| EZR | ezrin; Probably involved in connections of major cytoskeletal structures to the plasma membrane. |
| FADD | Fas (TNFRSF6)-associated via death domain; Apoptotic adaptor molecule that recruits caspase-8 or caspase-10 to the activated Fas (CD95) or TNFR-1 receptors. The resulting aggregate called the death-inducing signaling complex (DISC) performs caspase-8 proteolytic activation. |
| FAF1 | Fas (TNFRSF6) associated factor 1; Potentiates but cannot initiate FAS-induced apoptosis |
| FAM71C | family with sequence similarity 71, member C |
| FAS | Fas (TNF receptor superfamily, member 6); Receptor for TNFSF6/FASLG. The adapter molecule FADD recruits caspase-8 to the activated receptor. The resulting death- inducing signaling complex (DISC) performs caspase-8 proteolytic activation which initiates the subsequent cascade of caspases (aspartate-specific cysteine proteases) mediating apoptosis. |
| FASLG | Fas ligand (TNF superfamily, member 6); Cytokine that binds to TNFRSF6/FAS, a receptor that transduces the apoptotic signal into cells. |
| FBN1 | fibrillin 1; Fibrillins are structural components of 10-12 nm extracellular calcium-binding microfibrils, which occur either in association with elastin or in elastin-free bundles. |
| FBXO42 | F-box protein 42; Substrate-recognition component of some SCF (SKP1-CUL1- F-box protein)-type E3 ubiquitin ligase complex. Specifically recognizes p53/TP53, promoting its ubiquitination and degradation |
| FGD1 | RhoGEF and PH domain containing 1; Activates CDC42, a member of the Ras-like family of Rho- and Rac proteins, by exchanging bound GDP for free GTP. |
| FHOD1 | formin homology 2 domain containing 1; Required for the assembly of F-actin structures, such as stress fibers. Depends on the Rho-ROCK cascade for its activity. |
| FLNA | filamin A, alpha; Promotes orthogonal branching of actin filaments and links actin filaments to membrane glycoproteins. Anchors various transmembrane proteins to the actin cytoskeleton and serves as a scaffold for a wide range of cytoplasmic signaling proteins. |
| FMNL1 | formin-like 1; May play a role in the control of cell motility and survival of macrophages (By similarity) |
| FNBP1 | formin binding protein 1; May act as a link between RND2 signaling and regulation of the actin cytoskeleton (By similarity). Required to coordinate membrane tubulation with reorganization of the actin cytoskeleton during endocytosis. |
| FNBP1L | formin binding protein 1-like; Required to coordinate membrane tubulation with reorganization of the actin cytoskeleton during endocytosis. |
| FNTA | farnesyltransferase, CAAX box, alpha; Catalyzes the transfer of a farnesyl or geranyl-geranyl moiety from farnesyl or geranyl-geranyl pyrophosphate to a cysteine at the fourth position from the C-terminus of several proteins having the C-terminal sequence Cys-aliphatic-aliphatic-X. |
| FNTB | farnesyltransferase, CAAX box, beta; Catalyzes the transfer of a farnesyl moiety from farnesyl pyrophosphate to a cysteine at the fourth position from the C-terminus of several proteins. |
| FOXC1 | forkhead box C1; Binding of FREAC-3 and FREAC-4 to their cognate sites results in bending of the DNA at an angle of 80-90 degrees |
| FRK | fyn-related kinase; Positively regulates PTEN protein stability through phosphorylation of PTEN on 'Tyr-336', which in turn prevents its ubiquitination and degradation possibly by reducing its binding to NEDD4. |
| FRS2 | fibroblast growth factor receptor substrate 2; Adapter protein that links FGR and NGF receptors to downstream signaling pathways. Involved in the activation of MAP kinases. |
| FRS3 | fibroblast growth factor receptor substrate 3; Adapter protein that links FGR and NGF receptors to downstream signaling pathways. Involved in the activation of MAP kinases. |
| FYN | FYN oncogene related to SRC, FGR, YES; Implicated in the control of cell growth. Plays a role in the regulation of intracellular calcium levels, with isoform 2 showing the greater ability to mobilize cytoplasmic calcium in comparison to isoform 1. |
| GABARAPL2 | receptor-associated protein-like 2; Involved in intra-Golgi traffic. Modulates intra-Golgi transport through coupling between NSF activity and SNAREs activation. |
| GAPDH | glyceraldehyde-3-phosphate dehydrogenase; Independent of its glycolytic activity it is also involved in membrane trafficking in the early secretory pathway |
| GCC1 | GRIP and coiled-coil domain containing 1; Probably involved in maintaining Golgi structure |
| GCC2 | GRIP and coiled-coil domain containing 2; Probably involved in maintaining Golgi structure (By similarity) |
| GCH1 | GTP cyclohydrolase 1; Positively regulates nitric oxide synthesis in umbilical vein endothelial cells (HUVECs). May be involved in dopamine synthesis. |
| GDI1 | GDP dissociation inhibitor 1; Regulates the GDP/GTP exchange reaction of most Rab proteins by inhibiting the dissociation of GDP from them, and the subsequent binding of GTP to them |
| GDI2 | GDP dissociation inhibitor 2; Regulates the GDP/GTP exchange reaction of most Rab proteins by inhibiting the dissociation of GDP from them, and the subsequent binding of GTP to them |
| GEM | GTP binding protein overexpressed in skeletal muscle; Could be a regulatory protein, possibly participating in receptor-mediated signal transduction at the plasma membrane. |
| GGA1 | golgi associated, gamma adaptin ear containing, ARF binding protein 1; Plays a role in protein sorting and trafficking between the trans-Golgi network (TGN) and endosomes |
| GGA2 | golgi associated, gamma adaptin ear containing, ARF binding protein 2; Plays a role in protein sorting and trafficking between the trans-Golgi network (TGN) and endosomes. |
| GGA3 | golgi associated, gamma adaptin ear containing, ARF binding protein 3; Plays a role in protein sorting and trafficking between the trans-Golgi network (TGN) and endosomes. |
| GMIP | GEM interacting protein; Stimulates, in vitro and in vivo, the GTPase activity of RhoA |
| GNA12 | guanine nucleotide binding protein (G protein) alpha 12; Guanine nucleotide-binding proteins (G proteins) are involved as modulators or transducers in various transmembrane signaling systems |
| GNAI1 | guanine nucleotide binding protein (G protein), alpha inhibiting activity polypeptide 1; Guanine nucleotide-binding proteins (G proteins) are involved as modulators or transducers in various transmembrane signaling systems. |
| GNAI2 | guanine nucleotide binding protein (G protein), alpha inhibiting activity polypeptide 2; Guanine nucleotide-binding proteins (G proteins) are involved as modulators or transducers in various transmembrane signaling systems. |
| GNB1 | guanine nucleotide binding protein (G protein), beta polypeptide 1; Guanine nucleotide-binding proteins (G proteins) are involved as a modulator or transducer in various transmembrane signaling systems. |
| GOLGA1 | golgi autoantigen, golgin subfamily a, 1; Probably involved in maintaining Golgi structure |
| GOLGA2 | golgi autoantigen, golgin subfamily a, 2; Golgi auto-antigen; probably involved in maintaining cis-Golgi structure |
| GOLGA4 | golgi autoantigen, golgin subfamily a, 4; May play a role in vesicular transport from the trans- Golgi |
| GOLGA5 | golgi autoantigen, golgin subfamily a, 5; Involved in maintaining Golgi structure. Stimulates the formation of Golgi stacks and ribbons. |
| GOPC | golgi associated PDZ and coiled-coil motif containing; Plays a role in intracellular protein trafficking and degradation. May regulate CFTR chloride currents and acid-induced ACCN3 currents by modulating cell surface expression of both channels. |
| GORASP2 | golgi reassembly stacking protein 2, 55kDa; May be involved in assembly and membrane stacking of the Golgi cisternae, and in the process by which Golgi stacks reform after mitotic breakdown. |
| GOSR1 | golgi SNAP receptor complex member 1; Involved in transport from the ER to the Golgi apparatus as well as in intra-Golgi transport (By similarity) |
| GOSR2 | golgi SNAP receptor complex member 2; Involved in transport of proteins from the cis/medial- Golgi to the trans-Golgi network |
| GPSM2 | G-protein signaling modulator 2 (AGS3-like, C. elegans); Plays an important role in spindle pole orientation. Interacts and contributes to the functional activity of G(i) alpha proteins. |
| GRB10 | growth factor receptor-bound protein 10; Plays a functional role in insulin and IGF-I signaling. |
| GRB14 | growth factor receptor-bound protein 14; Interacts with the cytoplasmic domain of the autophosphorylated insulin receptor which is then inhibited. |
| GRB2 | growth factor receptor-bound protein 2; Adapter protein that provides a critical link between cell surface growth factor receptors and the Ras signaling pathway |
| GRB7 | growth factor receptor-bound protein 7; Interacts with the cytoplasmic domain of the epidermal growth factor receptor which is then inhibited. |
| GRLF1 | glucocorticoid receptor DNA binding factor 1; Represses transcription of the glucocorticoid receptor by binding to the cis-acting regulatory sequence 5'- GAGAAAAGAAACTGGAGAAACTC-3'. |
| GZMB | granzyme B (granzyme 2, cytotoxic T-lymphocyte-associated serine esterase 1); This enzyme is necessary for target cell lysis in cell- mediated immune responses. |
| H2AFY | H2A histone family, member Y; Variant histone H2A which replaces conventional H2A in a subset of nucleosomes where it represses transcription. Nucleosomes wrap and compact DNA into chromatin, limiting DNA accessibility to the cellular machineries which require DNA as a template. Histones thereby play a central role in transcription regulation, DNA repair, DNA replication and chromosomal stability. |
| HDAC1 | histone deacetylase 1; Responsible for the deacetylation of lysine residues on the N-terminal part of the core histones (H2A, H2B, H3 and H4). Histone deacetylation gives a tag for epigenetic repression and plays an important role in transcriptional regulation, cell cycle progression and developmental events. |
| HGF | hepatocyte growth factor (hepapoietin A; scatter factor); HGF is a potent mitogen for mature parenchymal hepatocyte cells, seems to be an hepatotrophic factor, and acts as growth factor for a broad spectrum of tissues and cell types. |
| HGS | hepatocyte growth factor-regulated tyrosine kinase substrate; Involved in intracellular signal transduction mediated by cytokines and growth factors. |
| HNF1A | HNF1 homeobox A; Required for the expression of several liver specific genes. Binds to the inverted palindrome 5'-GTTAATNATTAAC-3' |
| HPS4 | Hermansky-Pudlak syndrome 4; May function in the pathway of organelle biogenesis |
| HRAS | v-Ha-ras Harvey rat sarcoma viral oncogene homolog; Ras proteins bind GDP/GTP and possess intrinsic GTPase activity |
| HSPA1A | heat shock 70kDa protein 1A |
| ICMT | isoprenylcysteine carboxyl methyltransferase; Catalyzes the post-translational methylation of isoprenylated C-terminal cysteine residues |
| ID3 | inhibitor of DNA binding 3, dominant negative helix-loop-helix protein; ID (inhibitor of DNA binding) HLH proteins lack a basic DNA-binding domain but are able to form heterodimers with other HLH proteins, thereby inhibiting DNA binding. |
| IFNGR1 | interferon gamma receptor 1; Receptor for interferon gamma. |
| IKBKG | inhibitor of kappa light polypeptide gene enhancer in B-cells, kinase gamma; Regulatory subunit of the IKK core complex which phosphorylates inhibitors of NF-kappa-B thus leading to the dissociation of the inhibitor/NF-kappa-B complex and ultimately the degradation of the inhibitor. |
| IKZF3 | IKAROS family zinc finger 3 (Aiolos); Transcription factor that plays an important role in the regulation of lymphocyte differentiation |
| IL1RAP | interleukin 1 receptor accessory protein; Mediates interleukin-1-dependent activation of NF-kappa- B. Isoform 1 is part of the membrane-bound form of the IL-1 receptor. |
| IL3 | interleukin 3 (colony-stimulating factor, multiple); Granulocyte/macrophage colony-stimulating factors are cytokines that act in hematopoiesis by controlling the production, differentiation, and function of 2 related white cell populations of the blood, the granulocytes and the monocytes-macrophages |
| INSR | insulin receptor; This receptor binds insulin and has a tyrosine-protein kinase activity. Isoform Short has a higher affinity for insulin. |
| IPO11 | Functions in nuclear protein import as nuclear transport receptor. |
| IPO5 | importin 5; Functions in nuclear protein import as nuclear transport receptor. Serves as receptor for nuclear localization signals (NLS) in cargo substrates. |
| IPO7 | importin 7; Functions in nuclear protein import, either by acting as autonomous nuclear transport receptor or as an adapter-like protein in association with the importin-beta subunit KPNB1. |
| IQGAP1 | IQ motif containing GTPase activating protein 1; Binds to activated CDC42 but does not stimulate its GTPase activity. It associates with calmodulin. |
| IQGAP2 | IQ motif containing GTPase activating protein 2; Binds to activated CDC42 and RAC1 but does not seem to stimulate their GTPase activity. Associates with calmodulin |
| IRAK1 | interleukin-1 receptor-associated kinase 1; Binds to the IL-1 type I receptor following IL-1 engagement, triggering intracellular signaling cascades leading to transcriptional up-regulation and mRNA stabilization. |
| IRAK2 | interleukin-1 receptor-associated kinase 2; Binds to the IL-1 type I receptor following IL-1 engagement, triggering intracellular signaling cascades leading to transcriptional up-regulation and mRNA stabilization |
| ITGB1 | integrin, beta 1 (fibronectin receptor, beta polypeptide, antigen CD29 includes MDF2, MSK12) |
| ITPR1 | inositol 1,4,5-triphosphate receptor, type 1; Intracellular channel that mediates calcium release from the endoplasmic reticulum following stimulation by inositol 1,4,5- trisphosphate |
| ITSN1 | intersectin 1 (SH3 domain protein); Adapter protein that may provide indirect link between the endocytic membrane traffic and the actin assembly machinery. |
| ITSN2 | intersectin 2; Adapter protein that may provide indirect link between the endocytic membrane traffic and the actin assembly machinery. |
| JUP | junction plakoglobin; Common junctional plaque protein. The membrane- associated plaques are architectural elements in an important strategic position to influence the arrangement and function of both the cytoskeleton and the cells within the tissue. |
| KALRN | kalirin, RhoGEF kinase; Promotes the exchange of GDP by GTP. Activates specific Rho GTPase family members, thereby inducing various signaling mechanisms that regulate neuronal shape, growth, and plasticity, through their effects on the actin cytoskeleton. Induces lamellipodia independent of its GEF activity |
| KCNA2 | potassium voltage-gated channel, shaker-related subfamily, member 2; Mediates the voltage-dependent potassium ion permeability of excitable membranes. Assuming opened or closed conformations in response to the voltage difference across the membrane, the protein forms a potassium-selective channel through which potassium ions may pass in accordance with their electrochemical gradient |
| KCTD10 | potassium channel tetramerisation domain containing 10 |
| KDELR1 | KDEL (Lys-Asp-Glu-Leu) endoplasmic reticulum protein retention receptor 1; Required for the retention of luminal endoplasmic reticulum proteins. |
| KDM5B | lysine (K)-specific demethylase 5B; Histone demethylase that demethylates 'Lys-4' of histone H3, thereby playing a central role in histone code. |
| KIAA0209 | dedicator of cytokinesis 2; Involved in cytoskeletal rearrangements required for lymphocyte migration in response of chemokines. Activates RAC1 and RAC2 small GTPases, probably by functioning as a guanine nucleotide exchange factor (GEF) |
| KIAA2026 | putative uncharacterized protein |
| KIF20A | kinesin family member 20A; Interacts with guanosine triphosphate (GTP)-bound forms of RAB6A and RAB6B. May act as a motor required for the retrograde RAB6 regulated transport of Golgi membranes and associated vesicles along microtubules. |
| KIF23 | kinesin family member 23; Plus-end-directed motor enzyme that moves antiparallel microtubules in vitro. Localizes to the interzone of mitotic spindles |
| KIF3B | kinesin family member 3B; Involved in tethering the chromosomes to the spindle pole and in chromosome movement. Microtubule-based anterograde translocator for membranous organelles. |
| KPNA2 | karyopherin alpha 2 (RAG cohort 1, importin alpha 1); Functions in nuclear protein import as an adapter protein for nuclear receptor KPNB1. |
| KPNA4 | karyopherin alpha 4 (importin alpha 3); Functions in nuclear protein import as an adapter protein for nuclear receptor KPNB1. |
| KPNA6 | karyopherin alpha 6 (importin alpha 7); Functions in nuclear protein import as an adapter protein for nuclear receptor KPNB1. |
| KPNB1 | karyopherin (importin) beta 1; Functions in nuclear protein import, either in association with an adapter protein, like an importin-alpha subunit, which binds to nuclear localization signals (NLS) in cargo substrates, or by acting as autonomous nuclear transport receptor. |
| KPTN | May be involved in actin dynamics. May play a role in producing the sensory apparatus in hair cells. |
| KRAS | v-Ki-ras2 Kirsten rat sarcoma viral oncogene homolog; Ras proteins bind GDP/GTP and possess intrinsic GTPase activity |
| KRIT1 | KRIT1, ankyrin repeat containing |
| KRT28 | Essential for the proper assembly of types I and II keratin protein complexes and the formation of keratin intermediate filaments in the inner root sheath (irs) (By similarity) |
| KRT81 | keratin 81 |
| KTN1 | kinectin 1 (kinesin receptor); Receptor for kinesin thus involved in kinesin-driven vesicle motility. |
| LATS1 | Serine/threonine-protein kinase LATS1 (EC 2.7.11.1)(Large tumor suppressor homolog 1)(WARTS protein kinase)(h-warts); Tumor suppressor which plays a critical role in maintenance of ploidy through its actions in both mitotic progression and the G1 tetraploidy checkpoint |
| LCK | lymphocyte-specific protein tyrosine kinase; Tyrosine kinase that plays an essential role for the selection and maturation of developing T-cell in the thymus and in mature T-cell function. |
| LGALS1 | lectin, galactoside-binding, soluble, 1; May regulate apoptosis, cell proliferation and cell differentiation. Binds beta-galactoside and a wide array of complex carbohydrates. Inhibits CD45 protein phosphatase activity and therefore the dephosphorylation of Lyn kinase |
| LRPAP1 | low density lipoprotein receptor-related protein associated protein 1; Interacts with LRP1/alpha-2-macroglobulin receptor and glycoprotein 330 |
| MAGI1 | membrane associated guanylate kinase, WW and PDZ domain containing 1; May play a role as scaffolding protein at cell-cell junctions. |
| MAP1A | microtubule-associated protein 1A; Structural protein involved in the filamentous cross- bridging between microtubules and other skeletal elements |
| MAP2K1 | mitogen-activated protein kinase kinase 1; Catalyzes the concomitant phosphorylation of a threonine and a tyrosine residue in a Thr-Glu-Tyr sequence located in MAP kinases. Activates ERK1 and ERK2 MAP kinases |
| MAP3K10 | mitogen-activated protein kinase kinase kinase 10; Activates the JUN N-terminal pathway (By similarity) |
| MAP3K11 | mitogen-activated protein kinase kinase kinase 11; Activates the JUN N-terminal pathway. Required for serum-stimulated cell proliferation and for mitogen and cytokine activation of MAPK14 (p38), MAPK3 (ERK) and MAPK8 (JNK1). |
| MAP3K4 | mitogen-activated protein kinase kinase kinase 4; Component of a protein kinase signal transduction cascade. |
| MAP4K2 | mitogen-activated protein kinase kinase kinase kinase 2; Enhances MAP3K1 oligomerization, which may relieve amino-terminal mediated MAP3K1 autoinhibition and lead to activation following autophosphorylation. |
| MAP4K4 | mitogen-activated protein kinase kinase kinase kinase 4; Serine/threonine kinase that may play a role in the response to environmental stress and cytokines such as TNF-alpha. Appears to act upstream of the JUN N-terminal pathway |
| MAPK3 | mitogen-activated protein kinase 3; Involved in both the initiation and regulation of meiosis, mitosis, and postmitotic functions in differentiated cells by phosphorylating a number of transcription factors such as ELK-1. |
| MAPK8 | mitogen-activated protein kinase 8; Responds to activation by environmental stress and pro- inflammatory cytokines by phosphorylating a number of transcription factors, primarily components of AP-1 such as JUN, JDP2 and ATF2 and thus regulates AP-1 transcriptional activity. |
| MARK4 | MAP/microtubule affinity-regulating kinase 4 |
| MBP | myelin basic protein; The classic group of MBP isoforms (isoform 4-isoform 14) are with PLP the most abundant protein components of the myelin membrane in the CNS. |
| MCF2 | MCF.2 cell line derived transforming sequence-like; Guanine nucleotide exchange factor that potentially links pathways that signal through RAC1, RHOA and CDC42 |
| MCF2L | MCF.2 cell line derived transforming sequence-like; Guanine nucleotide exchange factor that potentially links pathways that signal through RAC1, RHOA and CDC42. |
| MCF2L2 | MCF.2 cell line derived transforming sequence-like 2; Probably functions as a guanine nucleotide exchange factor (By similarity) |
| MDM4 | Mdm4 p53 binding protein homolog (mouse); Inhibits p53- and p73-mediated cell cycle arrest and apoptosis by binding its transcriptional activation domain. |
| MED31 | mediator complex subunit 31; Component of the Mediator complex, a coactivator involved in the regulated transcription of nearly all RNA polymerase II-dependent genes. |
| MEOX2 | mesenchyme homeobox 2; Role in mesoderm induction and its earliest regional specification, somitogenesis, and myogenic and sclerotomal differentiation. |
| MEPCE | methylphosphate capping enzyme; S-adenosyl-L-methionine-dependent methyltransferase that adds a methylphosphate cap at the 5'-end of 7SK snRNA, leading to stabilize it |
| METAP2 | methionyl aminopeptidase 2; Removes the amino-terminal methionine from nascent proteins (By similarity) |
| MGC189703 | Uncharacterized protein KIAA0819 ; Associated with spermatid development (By similarity) |
| MICAL1 | microtubule associated monoxygenase, calponin and LIM domain containing 1; May be a cytoskeletal regulator that connects NEDD9 to intermediate filaments |
| MICALCL | MICAL C-terminal like; May cooperate with MAPK1/ERK2 via an intracellular signal transduction pathway in the morphogenetic development of round spermatids to spermatozoa (By similarity) |
| MICALL1 | MICAL-like 1; May be a cytoskeletal regulator |
| MINK1 | misshapen-like kinase 1 (zebrafish); Serine/threonine kinase that may play a role in the response to environmental stress. Appears to act upstream of the JUN N-terminal pathway |
| MIP | major intrinsic protein of lens fiber; Water channel. May be responsible for regulating the osmolarity of the lens |
| MIROI | Mitochondrial transport |
| MIRO2 | Mitochondrial transport |
| MLLT4 | myeloid/lymphoid or mixed-lineage leukemia (trithorax homolog, Drosophila); translocated to, 4; Belongs to an adhesion system, probably together with the E-cadherin-catenin system, which plays a role in the organization of homotypic, interneuronal and heterotypic cell-cell adherens junctions (AJs). Nectin- and actin-filament-binding protein that connects nectin to the actin cytoskeleton |
| MLPH | melanophilin; Rab effector protein involved in melanosome transport. Serves as link between melanosome-bound RAB27A and the motor protein MYO5A |
| MPP5 | membrane protein, palmitoylated 5 (MAGUK p55 subfamily member 5); May play a role in tight junctions biogenesis and in the establishment of cell polarity in epithelial cells. |
| MPRIP | myosin phosphatase Rho interacting protein; Targets myosin phosphatase to the actin cytoskeleton. |
| MRAS | muscle RAS oncogene homolog; May serve as an important signal transducer for a novel upstream stimuli in controlling cell proliferation. Weakly activates the MAP kinase pathway |
| MSN | moesin; Probably involved in connections of major cytoskeletal structures to the plasma membrane |
| MTNR1A | melatonin receptor 1A; High affinity receptor for melatonin. Likely to mediates the reproductive and circadian actions of melatonin. The activity of this receptor is mediated by pertussis toxin sensitive G proteins that inhibit adenylate cyclase activity |
| MTOR | mechanistic target of rapamycin (serine/threonine kinase); Kinase subunit of both mTORC1 and mTORC2, which regulate cell growth and survival in response to nutrient and hormonal signals. |
| MUC12 | cell surface associated; Involved in epithelial cell protection, adhesion modulation, and signaling. |
| MYC | v-myc myelocytomatosis viral oncogene homolog (avian); Participates in the regulation of gene transcription. |
| MYD88 | myeloid differentiation primary response gene (88); Adapter protein involved in the Toll-like receptor and IL-1 receptor signaling pathway in the innate immune response. |
| MYO5A | myosin VA (heavy chain 12, myoxin); Processive actin-based motor that can move in large steps approximating the 36-nm pseudo-repeat of the actin filament. Involved in melanosome transport. |
| MYO5B | myosin VB; May be involved in vesicular trafficking via its association with the CART complex. The CART complex is necessary for efficient transferrin receptor recycling but not for EGFR degradation |
| MYO6 | Myosins are actin-based motor molecules with ATPase activity. Unconventional myosins serve in intracellular movements. Myosin 6 is a reverse-direction motor protein that moves towards the minus-end of actin filaments. |
| MYRIP | myosin VIIA and Rab interacting protein; Rab effector protein involved in melanosome transport. Serves as link between melanosome-bound RAB27A and the motor proteins MYO5A and MYO7A. May link RAB27A-containing vesicles to actin filaments (By similarity) |
| NAV2 | neuron navigator 2; Possesses 3' to 5' helicase activity and exonuclease activity. Involved in neuronal development, specifically in the development of different sensory organs |
| NCF1 | neutrophil cytosolic factor 1; May be required for activation of the latent NADPH oxidase |
| NCF2 | neutrophil cytosolic factor 2; NCF2, NCF1, and a membrane bound cytochrome b558 are required for activation of the latent NADPH oxidase |
| NCK1 | NCK adaptor protein 1; Adapter protein which associates with tyrosine- phosphorylated growth factor receptors or their cellular substrates. |
| NCK2 | Adapter protein which associates with tyrosine- phosphorylated growth factor receptors or their cellular substrates. |
| NCKAP1 | Is part of lamellipodial complex that controls Rac- dependent actin remodeling |
| NCKIPSD | NCK interacting protein with SH3 domain; Has an important role in stress fiber formation induced by active diaphanous protein homolog 1 (DRF1). |
| NDEL1 | nudE nuclear distribution gene E homolog (A. nidulans)-like 1; Facilitates the polymerization of neurofilaments from the individual subunits NEFH and NEFL (By similarity). |
| NEK2 | NIMA (never in mitosis gene a)-related kinase 2; Protein kinase that is involved in mitotic regulation. May have a role at the G2-M transition. May also play a role in meiosis. |
| NEK9 | (never in mitosis gene a)- related kinase 9; Pleiotropic regulator of mitotic progression, participating in the control of spindle dynamics and chromosome separation. Phosphorylates different histones, myelin basic protein, beta-casein, and BICD2. Phosphorylates histone H3 on serine and threonine residues and beta-casein on serine residues. Important for G1/S transition and S phase progression |
| NET1 | neuroepithelial cell transforming 1; Acts as guanine nucleotide exchange factor (GEF) for RhoA GTPase. |
| NF1 | Stimulates the GTPase activity of Ras. NF1 shows greater affinity for Ras GAP, but lower specific activity. May be a regulator of Ras activity |
| NFKBIA | nuclear factor of kappa light polypeptide gene enhancer in B-cells inhibitor, alpha; Inhibits the activity of dimeric NF-kappa-B/REL complexes by trapping REL dimers in the cytoplasm through masking of their nuclear localization signals. |
| NFKBIB | nuclear factor of kappa light polypeptide gene enhancer in B-cells inhibitor, beta; Inhibits NF-kappa-B by complexing with and trapping it in the cytoplasm. |
| NGEF | neuronal guanine nucleotide exchange factor; Acts as a guanine nucleotide exchange factor |
| NKIRAS1 | NFKB inhibitor interacting Ras-like 1; Atypical Ras-like protein that acts as a potent regulator of NF-kappa-B activity by preventing the degradation of NF-kappa-B inhibitor beta (NFKBIB) by most signals, explaining why NFKBIB is more resistant to degradation. |
| NKIRAS2 | NFKB inhibitor interacting Ras-like 2; Atypical Ras-like protein that acts as a potent regulator of NF-kappa-B activity by preventing the degradation of NF-kappa-B inhibitor beta (NFKBIB) by most signals, explaining why NFKBIB is more resistant to degradation. |
| NM23-H2 | non-metastatic cells 2, protein (NM23B) expressed in; Major role in the synthesis of nucleoside triphosphates other than ATP. |
| NME1 | non-metastatic cells 1, protein (NM23A) expressed in; Major role in the synthesis of nucleoside triphosphates other than ATP. |
| NMT1 | N-myristoyltransferase 1; Adds a myristoyl group to the N-terminal glycine residue of certain cellular proteins (By similarity) |
| NOS1 | nitric oxide synthase 1 (neuronal); Produces nitric oxide (NO) which is a messenger molecule with diverse functions throughout the body. |
| NOS2 | nitric oxide synthase 2, inducible; Produces nitric oxide (NO) which is a messenger molecule with diverse functions throughout the body. |
| NOXA1 | NADPH oxidase activator 1; Functions as an activator of NOX1, a superoxide- producing NADPH oxidase. |
| NPM1 | nucleophosmin (nucleolar phosphoprotein B23, numatrin); Involved in diverse cellular processes such as ribosome biogenesis, centrosome duplication, protein chaperoning, histone assembly, cell proliferation, and regulation of tumor suppressors TP53/p53 and ARF. |
| NQO2 | NAD(P)H dehydrogenase, quinone 2; The enzyme apparently serves as a quinone reductase in connection with conjugation reactions of hydroquinones involved in detoxification pathways as well as in biosynthetic processes such as the vitamin K-dependent gamma-carboxylation of glutamate residues in prothrombin synthesis |
| NR3C1 | nuclear receptor subfamily 3, group C, member 1 (glucocorticoid receptor); Receptor for glucocorticoids (GC). Has a dual mode of action: as a transcription factor that binds to glucocorticoid response elements (GRE) and as a modulator of other transcription factors. Affects inflammatory responses, cellular proliferation and differentiation in target tissues. |
| NRAS | neuroblastoma RAS viral (v-ras) oncogene homolog; Ras proteins bind GDP/GTP and possess intrinsic GTPase activity |
| NRBP1 | nuclear receptor binding protein 1; May play a role in subcellular trafficking between the endoplasmic reticulum and Golgi apparatus through interactions with the Rho-type GTPases. |
| NTRK1 | neurotrophic tyrosine kinase, receptor, type 1; Required for high-affinity binding to nerve growth factor (NGF), neurotrophin-3 and neurotrophin-4/5 but not brain- derived neurotrophic factor (BDNF). |
| NUP153 | nucleoporin 153kDa; Possible DNA-binding subunit of the nuclear pore complex (NPC). The repeat-containing domain may be involved in anchoring components of the pore complex to the pore membrane |
| NUP50 | nucleoporin 50kDa; Component of the nuclear pore complex that has a direct role in nuclear protein export. |
| NUTF2 | nuclear transport factor 2; Facilitates protein transport into the nucleus. Interacts with the nucleoporin p62 and with Ran. Acts at a relatively late stage of nuclear protein import |
| NXF1 | nuclear RNA export factor 1; Involved in the nuclear export of mRNA species bearing retroviral constitutive transport elements (CTE) and in the export of mRNA from the nucleus to the cytoplasm. |
| NXT1 | NTF2-like export factor 1; Stimulator of protein export for NES-containing proteins. Also plays a role in the nuclear export of U1 snRNA, tRNA, and mRNA. |
| OBOE | regulating synaptic membrane exocytosis 2; Rab effector involved in exocytosis. May act as scaffold protein |
| OCLN | occludin; May play a role in the formation and regulation of the tight junction (TJ) paracellular permeability barrier. |
| OCRL | oculocerebrorenal syndrome of Lowe; Converts phosphatidylinositol 4,5-bisphosphate to phosphatidylinositol 4-phosphate. |
| OPHN1 | oligophrenin 1; Stimulates GTP hydrolysis of members of the Rho family. |
| OPRM1 | opioid receptor, mu 1; Inhibits neurotransmitter release by reducing calcium ion currents and increasing potassium ion conductance. Receptor for beta-endorphin |
| OPTN | optineurin; Plays a neuroprotective role in the eye and optic nerve. Probably part of the TNF-alpha signaling pathway that can shift the equilibrium toward induction of cell death. |
| OTUB1 | OTU domain, ubiquitin aldehyde binding 1; Hydrolase that can remove conjugated ubiquitin from proteins and plays an important regulatory role at the level of protein turnover by preventing degradation. |
| PAK1 | p21 protein (Cdc42/Rac)-activated kinase 1; The activated kinase acts on a variety of targets. Likely to be the GTPase effector that links the Rho-related GTPases to the JNK MAP kinase pathway. |
| PAK2 | p21 protein (Cdc42/Rac)-activated kinase 2; The activated kinase acts on a variety of targets. Phosphorylates ribosomal protein S6, histone H4 and myelin basic protein. |
| PAK3 | p21 protein (Cdc42/Rac)-activated kinase 3; Key regulator of synapse formation and plasticity in the hippocampus (By similarity |
| PAK4 | p21 protein (Cdc42/Rac)-activated kinase 4; Activates the JNK pathway. Plays a role in the reorganization of the actin cytoskeleton and in the formation of filopodia. |
| PAK6 | p21 protein (Cdc42/Rac)-activated kinase 6; The activated kinase acts on a variety of targets (By similarity) |
| PAK7 | p21 protein (Cdc42/Rac)-activated kinase 7; The activated kinase acts on a variety of targets (By similarity) |
| PARD3 | par-3 partitioning defective 3 homolog (C. elegans); Adapter protein involved in asymmetrical cell division and cell polarization processes. |
| PARD6A | par-6 partitioning defective 6 homolog alpha (C. elegans); Adapter protein involved in asymmetrical cell division and cell polarization processes. |
| PARD6B | par-6 partitioning defective 6 homolog beta (C. elegans); Adapter protein involved in asymmetrical cell division and cell polarization processes. |
| PARD6G | par-6 partitioning defective 6 homolog gamma (C. elegans); Adapter protein involved in asymmetrical cell division and cell polarization processes. |
| PDE6D | phosphodiesterase 6D, cGMP-specific, rod, delta |
| PDGFB | platelet-derived growth factor beta polypeptide (simian sarcoma viral (v-sis) oncogene homolog); Platelet-derived growth factor is a potent mitogen for cells of mesenchymal origin. |
| PDLIM7 | PDZ and LIM domain 7 (enigma); May function as a scaffold on which the coordinated assembly of proteins can occur. |
| PFN1 | profilin 1; Binds to actin and affects the structure of the cytoskeleton. At high concentrations, profilin prevents the polymerization of actin, whereas it enhances it at low concentrations. |
| PGGT1B | protein geranylgeranyltransferase type I, beta subunit; Catalyzes the transfer of a geranyl-geranyl moiety from geranyl-geranyl pyrophosphate to a cysteine at the fourth position from the C-terminus of proteins having the C-terminal sequence Cys-aliphatic-aliphatic-X. |
| PHGDH | phosphoglycerate dehydrogenase |
| PICK1 | protein interacting with PRKCA 1; Probable adapter protein that bind to and organize the subcellular localization of a variety of membrane proteins containing some PDZ recognition sequence. Involved in the clustering of various receptors, possibly by acting at the receptor internalization level. |
| PIGR | polymeric immunoglobulin receptor; This receptor binds polymeric IgA and IgM at the basolateral surface of epithelial cells. |
| PIK3CA | phosphoinositide-3-kinase, catalytic, alpha polypeptide; Phosphorylates PtdIns, PtdIns4P and PtdIns(4,5)P2 with a preference for PtdIns(4,5)P2 |
| PIK3CD | phosphoinositide-3-kinase, catalytic, delta polypeptide |
| PIK3CG | phosphoinositide-3-kinase, catalytic, gamma polypeptide; 3-phosphorylates the cellular phosphoinositide PtdIns- 4,5-biphosphate (PtdIns(4,5)P2) to produce PtdIns-3, 4,5- triiphosphate (PtdIns(3,4,5)P3). Links G-protein coupled receptor activation to the secondary messenger PtdIns(3,4,5)P3 production |
| PIK3R1 | phosphoinositide-3-kinase, regulatory subunit 1 (alpha); Binds to activated (phosphorylated) protein-Tyr kinases, through its SH2 domain, and acts as an adapter, mediating the association of the p110 catalytic unit to the plasma membrane. |
| PIN1 | peptidylprolyl cis/trans isomerase, NIMA-interacting 1; Essential PPIase that regulates mitosis presumably by interacting with NIMA and attenuating its mitosis-promoting activity. |
| PIP4K2A | phosphatidylinositol-5-phosphate 4-kinase, type II, alpha; Catalyzes the phosphorylation of phosphatidylinositol-5- phosphate on the fourth hydroxyl of the myo-inositol ring, to form phosphatidylinositol-4,5-biphosphate |
| PIP5K1A | phosphatidylinositol-4-phosphate 5-kinase, type I, alpha; Participates in the biosynthesis of phosphatidylinositol-4,5-bisphosphate. Mediates RAC1-dependent reorganization of actin filaments. |
| PIP5K1C | phosphatidylinositol-4-phosphate 5-kinase, type I, gamma; Plays a role in membrane ruffling and assembly of clathrin-coated pits at the synapse. Mediates RAC1-dependent reorganization of actin filaments (By similarity). Participates in the biosynthesis of phosphatidylinositol-4,5-bisphosphate" |
| PITPNM1 | phosphatidylinositol transfer protein, membrane-associated 1; Regulates RHOA activity, and plays a role in cytoskeleton remodeling. |
| PITPNM2 | phosphatidylinositol transfer protein, membrane-associated 2; Catalyzes the transfer of phosphatidylinositol and phosphatidylcholine between membranes (in vitro). Binds calcium ions |
| PITX2 | paired-like homeodomain 2; May play an important role in development and maintenance of anterior structures. |
| PKIG | protein kinase (cAMP-dependent, catalytic) inhibitor gamma; Extremely potent competitive inhibitor of cAMP-dependent protein kinase activity, |
| PKN1 | protein kinase N1; Can phosphorylate ribosomal protein S6. Mediates GTPase Rho dependent intracellular signaling |
| PKN2 | protein kinase N2; Exhibits a preference for highly basic protein substrates (By similarity) |
| PLAU | plasminogen activator, urokinase; Specifically cleave the zymogen plasminogen to form the active enzyme plasmin |
| PLCB2 | phospholipase C, beta 2; The production of the second messenger molecules diacylglycerol (DAG) and inositol 1,4,5-trisphosphate (IP3) is mediated by activated phosphatidylinositol-specific phospholipase C enzymes |
| PLCD1 | phospholipase C, delta 1; The production of the second messenger molecules diacylglycerol (DAG) and inositol 1,4,5-trisphosphate (IP3) is mediated by activated phosphatidylinositol-specific phospholipase C enzymes. |
| PLCE1 | phospholipase C, epsilon 1; The production of the second messenger molecules diacylglycerol (DAG) and inositol 1,4,5-trisphosphate (IP3) is mediated by activated phosphatidylinositol-specific phospholipase C enzymes. |
| PLCG1 | phospholipase C, gamma 1; PLC-gamma is a major substrate for heparin-binding growth factor 1 (acidic fibroblast growth factor)-activated tyrosine kinase |
| PLCG2 | phospholipase C, gamma 2 (phosphatidylinositol-specific); The production of the second messenger molecules diacylglycerol (DAG) and inositol 1,4,5-trisphosphate (IP3) is mediated by activated phosphatidylinositol-specific phospholipase C enzymes. |
| PLD1 | phospholipase D1, phosphatidylcholine-specific; Implicated as a critical step in numerous cellular pathways, including signal transduction, membrane trafficking, and the regulation of mitosis. May be involved in the regulation of perinuclear intravesicular membrane traffic |
| PLD2 | phospholipase D2; May have a role in signal-induced cytoskeletal regulation and/or endocytosis (By similarity) |
| PLEKHA8 | pleckstrin homology domain containing, family A (phosphoinositide binding specific) member 8; Involved in TGN-to-plasma membrane transport and in the formation of post-Golgi constitutive carriers. |
| PLEKHG2 | pleckstrin homology domain containing, family G (with RhoGef domain) member 2; May be a transforming oncogene with exchange activity for CDC42 (By similarity). |
| PLEKHM2 | pleckstrin homology domain containing, family M (with RUN domain) member 2 |
| PLIN2 | perilipin 2; May be involved in development and maintenance of adipose tissue |
| PLIN3 | Required for the transport of mannose 6-phosphate receptors (MPR) from endosomes to the trans-Golgi network |
| PLXNA1 | plexin A1; Coreceptor for SEMA3A, SEMA3C, SEMA3F and SEMA6D. Necessary for signaling by class 3 semaphorins and subsequent remodeling of the cytoskeleton. |
| PLXNB1 | plexin B1; Receptor for SEMA4D. Plays a role in RHOA activation and subsequent changes of the actin cytoskeleton. |
| PLXNB2 | plexin B2; Receptor for SEMA4D. Plays a role in RHOA activation and subsequent changes of the actin cytoskeleton. Plays a role in axon guidance, invasive growth and cell migration |
| PMM1 | phosphomannomutase 1; Involved in the synthesis of the GDP-mannose and dolichol-phosphate-mannose required for a number of critical mannosyl transfer reactions (By similarity) |
| PPIG | peptidylprolyl isomerase G (cyclophilin G); PPIases accelerate the folding of proteins. It catalyzes the cis-trans isomerization of proline imidic peptide bonds in oligopeptides. |
| PPP1R12A | protein phosphatase 1, regulatory (inhibitor) subunit 12A; Regulates myosin phosphatase activity |
| PPP2CA | protein phosphatase 2 (formerly 2A), catalytic subunit, alpha isoform; PP2A can modulate the activity of phosphorylase B kinase casein kinase 2, mitogen-stimulated S6 kinase, and MAP-2 kinase. |
| PPP2CB | protein phosphatase 2 (formerly 2A), catalytic subunit, beta isoform; PP2A can modulate the activity of phosphorylase B kinase casein kinase 2, mitogen-stimulated S6 kinase, and MAP-2 kinase. |
| PPP2R1A | protein phosphatase 2 (formerly 2A), regulatory subunit A, alpha isoform; The PR65 subunit of protein phosphatase 2A serves as a scaffolding molecule to coordinate the assembly of the catalytic subunit and a variable regulatory B subunit |
| PPP2R1B | protein phosphatase 2 (formerly 2A), regulatory subunit A, beta isoform; The PR65 subunit of protein phosphatase 2A serves as a scaffolding molecule to coordinate the assembly of the catalytic subunit and a variable regulatory B subunit |
| PPP2R2B | protein phosphatase 2 (formerly 2A), regulatory subunit B, beta isoform; The B regulatory subunit might modulate substrate selectivity and catalytic activity, and also might direct the localization of the catalytic enzyme to a particular subcellular compartment. |
| PPP2R2C | protein phosphatase 2 (formerly 2A), regulatory subunit B, gamma isoform; The B regulatory subunit might modulate substrate selectivity and catalytic activity, and also might direct the localization of the catalytic enzyme to a particular subcellular compartment |
| PPP2R5D | protein phosphatase 2, regulatory subunit B', delta isoform; The B regulatory subunit might modulate substrate selectivity and catalytic activity, and also might direct the localization of the catalytic enzyme to a particular subcellular compartment |
| PRAF2 | PRA1 domain family, member 2; May be involved in ER/Golgi transport and vesicular traffic. Plays a proapoptic role in cerulenin-induced neuroblastoma apoptosis |
| PRKACA | protein kinase, cAMP-dependent, catalytic, alpha; Phosphorylates a large number of substrates in the cytoplasm and the nucleus |
| PRKAR2A | protein kinase, cAMP-dependent, regulatory, type II, alpha; Type II regulatory chains mediate membrane association by binding to anchoring proteins, including the MAP2 kinase |
| PRKCA | protein kinase C, alpha; This is a calcium-activated, phospholipid-dependent, serine- and threonine-specific enzyme. May play a role in cell motility by phosphorylating CSPG4 |
| PRKCB | protein kinase C, beta; This is a calcium-activated, phospholipid-dependent, serine- and threonine-specific enzyme. PKC is activated by diacylglycerol which in turn phosphorylates a range of cellular proteins. |
| PRKCD | protein kinase C, delta; This is calcium-independent, phospholipid-dependent, serine- and threonine-specific enzyme. PKC is activated by diacylglycerol which in turn phosphorylates a range of cellular proteins. |
| PRKCG | protein kinase C, gamma; This is a calcium-activated, phospholipid-dependent, serine- and threonine-specific enzyme |
| PRKCI | protein kinase C, iota; Calcium-independent, phospholipid-dependent, serine- and threonine-specific kinase. May play a role in the secretory response to nutrients. |
| PRKCZ | protein kinase C, zeta; This is a calcium-independent, phospholipid-dependent, serine- and threonine-specific enzyme |
| PSEN1 | presenilin 1; Probable catalytic subunit of the gamma-secretase complex, an endoprotease complex that catalyzes the intramembrane cleavage of integral membrane proteins such as Notch receptors and APP (beta-amyloid precursor protein). |
| PSEN2 | presenilin 2 (Alzheimer disease 4); Probable catalytic subunit of the gamma-secretase complex, an endoprotease complex that catalyzes the intramembrane cleavage of integral membrane proteins such as Notch receptors and APP (beta-amyloid precursor protein). |
| PSMA7 | proteasome (prosome, macropain) subunit, alpha type, 7; The proteasome is a multicatalytic proteinase complex which is characterized by its ability to cleave peptides |
| PTK2 | PTK2 protein tyrosine kinase 2; Non-receptor protein-tyrosine kinase implicated in signaling pathways involved in cell motility, proliferation and apoptosis. |
| PTK2B | PTK2B protein tyrosine kinase 2 beta; Involved in calcium induced regulation of ion channel and activation of the map kinase signaling pathway. |
| PTMA | prothymosin, alpha; Prothymosin alpha may mediate immune function by conferring resistance to certain opportunistic infections |
| PTPLAD1 | protein tyrosine phosphatase-like A domain containing 1; Involved in Rac1-signaling pathways leading to the modulation of gene expression |
| PTPN1 | protein tyrosine phosphatase, non-receptor type 1; May play an important role in CKII- and p60c-src-induced signal transduction cascades (By similarity) |
| PTPN2 | protein tyrosine phosphatase, non-receptor type 2 |
| PXN | paxillin; Cytoskeletal protein involved in actin-membrane attachment at sites of cell adhesion to the extracellular matrix (focal adhesion) |
| RAB10 | RAB10, member RAS oncogene family; May be involved in vesicular trafficking and neurotransmitter release |
| RAB11A | RAB11A, member RAS oncogene family; Modulates endosomal trafficking |
| RAB11B | RAB11B, member RAS oncogene family; Modulates endosomal trafficking |
| RAB11FIP1 | RAB11 family interacting protein 1 (class I); A Rab11 effector protein involved in the endosomal recycling process. Also involved in controlling membrane trafficking along the phagocytic pathway and in phagocytosis |
| RAB11FIP2 | A Rab11 effector protein acting in the regulation of the transport of vesicles from the endosomal recycling compartment (ERC) to the plasma membrane. |
| RAB11FIP3 | RAB11 family interacting protein 3 (class II) |
| RAB11FIP4 | RAB11 family interacting protein 4 (class II); A Rab11 effector protein acting in the endosomal trafficking |
| RAB11FIP5 | RAB11 family interacting protein 5 (class I); Rab effector involved in protein trafficking from apical recycling endosomes to the apical plasma membrane |
| RAB13 | RAB13, member RAS oncogene family; Could participate in polarized transport, in the assembly and/or the activity of tight junctions |
| RAB14 | RAB14, member RAS oncogene family; May be involved in vesicular trafficking and neurotransmitter release |
| RAB15 | RAB15, member RAS onocogene family; May act in concert with RAB3A in regulating aspects of synaptic vesicle membrane flow within the nerve terminal (By similarity) |
| RAB17 | RAB17, member RAS oncogene family; Might be involved in transcellular transport (By similarity) |
| RAB18 | RAB18, member RAS oncogene family; Plays a role in apical endocytosis/recycling. |
| RAB19 |  |
| RAB1A | RAB1A, member RAS oncogene family; Probably required for transit of protein from the ER through Golgi compartment. |
| RAB1B | Probably required for transit of protein from the ER through Golgi compartment. |
| RAB20 | Plays a role in apical endocytosis/recycling |
| RAB21 | RAB21, member RAS oncogene family; Regulates integrin internalization and recycling, but does not influence the traffic of endosomally translocated receptors in general. |
| RAB22A | RAB22A, member RAS oncogene family |
| RAB24 | RAB24, member RAS oncogene family; May be involved in autophagy-related processes |
| RAB25 | member RAS oncogene family; May selectively regulate the apical recycling and/or transcytotic pathways |
| RAB26 | RAB26, member RAS oncogene family; Participates in exocrine secretion: regulates the secretion of acinar granules in the parotid gland (By similarity) |
| RAB27A | RAB27A, member RAS oncogene family; Plays a role in cytotoxic granule exocytosis in lymphocytes. Required for both granule maturation and granule docking and priming at the immunologic synapse |
| RAB27B | RAB27B, member RAS oncogene family; May be involved in targeting uroplakins to urothelial apical membranes (By similarity) |
| RAB2A | RAB2A, member RAS oncogene family; Required for protein transport from the endoplasmic reticulum to the Golgi complex |
| RAB32 | RAB32, member RAS oncogene family; Acts as an A-kinase anchoring protein by binding to the type II regulatory subunit of protein kinase A and anchoring it to the mitochondrion. Also involved in synchronization of mitochondrial fission |
| RAB33A | RAB33A, member RAS oncogene family |
| RAB34 | RAB34, member RAS oncogene family; Protein transport. Involved in the redistribution of lysosomes to the peri-Golgi region (By similarity) |
| RAB35 | RAB35, member RAS oncogene family; Possesses GTPase activity |
| RAB37 | RAB37, member RAS oncogene family |
| RAB3A | RAB3A, member RAS oncogene family; Involved in exocytosis by regulating a late step in synaptic vesicle fusion. Could play a role in neurotransmitter release by regulating membrane flow in the nerve terminal |
| RAB3B | member RAS oncogene family; Protein transport. Probably involved in vesicular traffic |
| RAB3C | RAB3C, member RAS oncogene family; Protein transport. Probably involved in vesicular traffic (By similarity) |
| RAB3D | RAB3D, member RAS oncogene family; Protein transport. Probably involved in regulated exocytosis (By similarity) |
| RAB3IL1 | Guanine nucleotide exchange factor (GEF) for Rab3A, a GTPase that regulates synaptic vesicle exocytosis (By similarity) |
| RAB3IP | RAB3A interacting protein (rabin3) |
| RAB4A | RAB4A, member RAS oncogene family; Protein transport. Probably involved in vesicular traffic (By similarity) |
| RAB4B | member RAS oncogene family; Protein transport. Probably involved in vesicular traffic (By similarity) |
| RAB5A | member RAS oncogene family; Required for the fusion of plasma membranes and early endosomes |
| RAB5B | RAB5B, member RAS oncogene family; Protein transport. Probably involved in vesicular traffic (By similarity) |
| RAB5C | member RAS oncogene family; Protein transport. Probably involved in vesicular traffic (By similarity) |
| RAB6A | member RAS oncogene family; Protein transport. Regulator of membrane traffic from the Golgi apparatus towards the endoplasmic reticulum |
| RAB6B | member RAS oncogene family; Seems to have a role in retrograde membrane traffic at the level of the Golgi complex. May function in retrograde tansport in neuronal cells |
| RAB7A | member RAS oncogene family; Involved in late endocytic transport. |
| RAB8A | member RAS oncogene family; May be involved in vesicular trafficking and neurotransmitter release |
| RAB8B | member RAS oncogene family; May be involved in vesicular trafficking and neurotransmitter release |
| RAB9A | member RAS oncogene family; Involved in the transport of proteins between the endosomes and the trans Golgi network |
| RAB9B | member RAS oncogene family; Involved in the transport of proteins between the endosomes and the trans Golgi network |
| RABAC1 | Rab acceptor 1 (prenylated); General Rab protein regulator required for vesicle formation from the Golgi complex. May control vesicle docking and fusion by mediating the action of Rab GTPases to the SNARE complexes. |
| RABEP1 | rabaptin, RAB GTPase binding effector protein 1; Rab effector protein acting as linker between gamma- adaptin, RAB4A and RAB5A. Involved in endocytic membrane fusion and membrane trafficking of recycling endosomes. Stimulates RABGEF1 mediated nucleotide exchange on RAB5A |
| RABEPK | Rab9 effector protein with kelch motifs; Rab9 effector required for endosome to trans-Golgi network (TGN) transport |
| RABGAP1 | RAB GTPase activating protein 1; May act as a GTPase-activating protein of RAB6A. May play a role in microtubule nucleation by centrosome. |
| RABGEF1 | RAB guanine nucleotide exchange factor (GEF) 1; Rab effector protein acting as linker between gamma- adaptin, RAB4A or RAB5A. Involved in endocytic membrane fusion and membrane trafficking of recycling endosomes. Stimulates nucleotide exchange on RAB5A. Can act as a ubiquitin ligase (By similarity) |
| RABGGTA | Rab geranylgeranyltransferase, alpha subunit; Catalyzes the transfer of a geranyl-geranyl moiety from geranyl-geranyl pyrophosphate to both cysteines in Rab proteins |
| RABGGTB | Catalyzes the transfer of a geranyl-geranyl moiety from geranyl-geranyl pyrophosphate to both cysteines in Rab proteins |
| RABIF | RAB interacting factor; Guanine-nucleotide-releasing protein that acts on members of the SCE4/YPT1/RAB subfamily. Stimulates GDP release from both YPT1 and RAB3A, but is less active on these proteins than on the SEC4 protein. |
| RAC1 | ras-related C3 botulinum toxin substrate 1 (rho family, small GTP binding protein Rac1); Plasma membrane-associated. In its active state, binds to a variety of effector proteins to regulate cellular responses such as secretory processes, phagocytosis of apoptotic cells, epithelial cell polarization and growth-factor induced formation of membrane ruffles |
| RAC2 | ras-related C3 botulinum toxin substrate 2 (rho family, small GTP binding protein Rac2) |
| RAC3 | ras-related C3 botulinum toxin substrate 3 (rho family, small GTP binding protein Rac3) |
| RACGAP1 | Rac GTPase activating protein 1 pseudogene; Essential for the early stages of embryogenesis and may play a role in the microtubule-dependent steps in cytokinesis. |
| RAF1 | v-raf-1 murine leukemia viral oncogene homolog 1; Involved in the transduction of mitogenic signals from the cell membrane to the nucleus. |
| RALA | v-ral simian leukemia viral oncogene homolog A (ras related) |
| RALB | ral simian leukemia viral oncogene homolog B (ras related; GTP binding protein) |
| RALBP1 | RALBP1 associated Eps domain containing 2; Involved in growth factor signaling through its influence on the Ral signaling pathway. ralA binding protein 1; Can activate specifically hydrolysis of GTP bound to RAC1 and CDC42, but not RALA. |
| RALGDS | ral guanine nucleotide dissociation stimulator; Stimulates the dissociation of GDP from the Ras-related RalA and RalB GTPases which allows GTP binding and activation of the GTPases. Interacts and acts as an effector molecule for R-Ras, H-Ras, K-Ras, and Rap |
| RALGPS1 | Ral GEF with PH domain and SH3 binding motif 1; May be involved in cytoskeletal organization (By similarity). |
| RAN | GTP-binding protein involved in nucleocytoplasmic transport. Required for the import of protein into the nucleus and also for RNA export. Involved in chromatin condensation and control of cell cycle (By similarity). |
| RANBP1 | RAN binding protein 10; May act as an adapter protein to couple membrane receptors to intracellular signaling pathways. In contrast to RANBP9, does not interact with Sos and does not activate the Ras pathway |
| RANBP10 | RAN binding protein 10; May act as an adapter protein to couple membrane receptors to intracellular signaling pathways. |
| RANBP2 | RAN binding protein 2; E3 SUMO-protein ligase which facilitates SUMO1 and SUMO2 conjugation by UBE2I. Involved in transport factor (Ran-GTP, karyopherin)-mediated protein import via the F-G repeat-containing domain which acts as a docking site for substrates. |
| RANBP3 | Functions in nuclear protein import as nuclear transport receptor. |
| RANBP9 | RAN binding protein 9; May act as an adapter protein to couple membrane receptors to intracellular signaling pathways. |
| RANGAP1 | Ran GTPase activating protein 1; GTPase activator for the nuclear Ras-related regulatory protein Ran |
| RANGRF | RAN guanine nucleotide release factor; May regulate the intracellular trafficking of RAN. |
| RAP1A | member of RAS oncogene family; Induces morphological reversion of a cell line transformed by a Ras oncogene. Counteracts the mitogenic function of Ras, at least partly because it can interact with Ras GAPs and RAF in a competitive manner |
| RAP1B | RAP1B, member of RAS oncogene family; Induces morphological reversion of a cell line transformed by a Ras oncogene. Counteracts the mitogenic function of Ras, at least partly because it can interact with Ras GAPs and RAF in a competitive manner |
| RAP1GAP | RAP1 GTPase activating protein; GTPase activator for the nuclear Ras-related regulatory protein RAP-1A (KREV-1), converting it to the putatively inactive GDP-bound state |
| RAP1GDS1 | RAP1, GTP-GDP dissociation stimulator 1; Stimulates GDP/GTP exchange reaction of a group of small GTP-binding proteins (G proteins) including Rap1a/Rap1b, RhoA, RhoB and KRas, by stimulating the dissociation of GDP from and the subsequent binding of GTP to each small G protein |
| RAP2A | RAP2A, member of RAS oncogene family |
| RAP2B | RAP2B, member of RAS oncogene family |
| RAPGEF1 | Rap guanine nucleotide exchange factor (GEF) 1; Guanine nucleotide-releasing protein that binds to SH3 domain of CRK and GRB2/ASH. Transduces signals from CRK to activate RAS |
| RAPGEF2 | Rap guanine nucleotide exchange factor (GEF) 2; Guanine nucleotide exchange factor (GEF) for Rap1A, Rap1B and Rap2B GTPases. Does not interact with cAMP or cGMP |
| RAPGEF3 | Rap guanine nucleotide exchange factor (GEF) 3; Guanine nucleotide exchange factor (GEF) for RAP1A and RAP2A small GTPases that is activated by binding cAMP |
| RAPGEF4 | Rap guanine nucleotide exchange factor (GEF) 4; Guanine nucleotide exchange factor (GEF) for RAP1A, RAP1B and RAP2A small GTPases that is activated by binding cAMP. |
| RAPGEF5 | Rap guanine nucleotide exchange factor (GEF) 5; Guanine nucleotide exchange factor (GEF) for RAP1A, RAP2A and MRAS/M-Ras-GTP. Its association with MRAS inhibits Rap1 activation |
| RAPGEF6 | Rap guanine nucleotide exchange factor (GEF) 6; Guanine nucleotide exchange factor (GEF) for Rap1A, Rap2A and M-Ras GTPases. Does not interact with cAMP |
| RARRES3 | retinoic acid receptor responder (tazarotene induced) 3; May be a growth regulator that mediates some of the growth suppressive effects of retinoids |
| RASA1 | RAS p21 protein activator (GTPase activating protein) 1; Inhibitory regulator of the Ras-cyclic AMP pathway. Stimulates the GTPase of normal but not oncogenic Ras p21 |
| RASA3 | RAS p21 protein activator 3; Inhibitory regulator of the Ras-cyclic AMP pathway. Binds inositol tetrakisphosphate (IP4) with high affinity. Might be a specific IP4 receptor |
| RASD1 | RAS, dexamethasone-induced 1; Small GTPase. Negatively regulates the transcription regulation activity of the APBB1/FE65-APP complex via its interaction with APBB1/FE65 (By similarity) |
| RASGRF1 | protein-specific guanine nucleotide-releasing factor 1; Promotes the exchange of Ras-bound GDP by GTP |
| RASGRP1 | RAS guanyl releasing protein 1 (calcium and DAG-regulated); Functions as a diacylglycerol (DAG)-regulated nucleotide exchange factor specifically activating Ras through the exchange of bound GDP for GTP. |
| RASGRP2 | RAS guanyl releasing protein 2 (calcium and DAG-regulated); Functions as a calcium- and DAG-regulated nucleotide exchange factor specifically activating Rap through the exchange of bound GDP for GTP. May also activates other GTPases such as RRAS, RRAS2, NRAS, KRAS but not HRAS |
| RASGRP4 | RAS guanyl releasing protein 4; Functions as a cation- and diacylglycerol (DAG)- regulated nucleotide exchange factor activating Ras through the exchange of bound GDP for GTP. May function in mast cells differentiation |
| RASIP1 | Ras interacting protein 1; May act as effector for Golgi-bound HRAS and other Ras- like proteins. May promote HRAS-mediated transformation |
| RASSF1 | Ras association (RalGDS/AF-6) domain family member 1; Potential tumor suppressor. Required for death receptor- dependent apoptosis. |
| RASSF2 | Ras association (RalGDS/AF-6) domain family member 2; Potential tumor suppressor. Acts as a KRAS-specific effector protein. May promote apoptosis and cell cycle arrest |
| RASSF3 | Ras association (RalGDS/AF-6) domain family member 3 |
| RASSF5 | Ras association (RalGDS/AF-6) domain family member 5; Potential tumor suppressor. Seems to be involved in lymphocyte adhesion by linking RAP1A activation upon T cell receptor or chemokine stimulation to integrin activation. |
| RCC1 | regulator of chromosome condensation 1; Promotes the exchange of Ran-bound GDP by GTP. |
| RCC2 | regulator of chromosome condensation 2; Required for completion of mitosis and cytokinesis. |
| REM1 | RAS (RAD and GEM)-like GTP-binding 1; Promotes endothelial cell sprouting and actin cytoskeletal reorganization. May be involved in angiogenesis. May function in Ca(2+) signaling |
| REPS1 | associated Eps domain containing 1; May coordinate the cellular actions of activated EGF receptors and Ral-GTPases (By similarity) |
| REPS2 | RALBP1 associated Eps domain containing 2; Involved in growth factor signaling through its influence on the Ral signaling pathway |
| RGL1 | ral guanine nucleotide dissociation stimulator-like 1; Probable guanine nucleotide exchange factor |
| RGL2 | ral guanine nucleotide dissociation stimulator-like 2 |
| RGL4 | ral guanine nucleotide dissociation stimulator-like 4 |
| RGNEF | Rho-guanine nucleotide exchange factor (p190RhoGEF); Functions as a RHOA-specific guanine nucleotide exchange factor regulating signaling pathways downstream of integrins and growth factor receptors. |
| RGPD5 | RANBP2-like and GRIP domain containing 5 |
| RGS12 | regulator of G-protein signaling 12; Inhibits signal transduction by increasing the GTPase activity of G protein alpha subunits thereby driving them into their inactive GDP-bound form |
| RGS14 | regulator of G-protein signaling 14; Inhibits signal transduction by increasing the GTPase activity of G protein alpha subunits thereby driving them into their inactive GDP-bound form |
| RHEB | Ras homolog enriched in brain; Stimulates the phosphorylation of S6K1 and EIF4EBP1 through activation of mTORC1 signaling. Activates the protein kinase activity of mTORC1. Has low intrinsic GTPase activity |
| RHOA | ras homolog gene family, member A; Regulates a signal transduction pathway linking plasma membrane receptors to the assembly of focal adhesions and actin stress fibers. |
| RHOB | ras homolog gene family, member B; Mediates apoptosis in neoplastically transformed cells after DNA damage. Not essential for development but affects cell adhesion and growth factor signaling in transformed cells. |
| RHOBTB1 | Rho-related BTB domain containing 1 |
| RHOBTB2 | Rho-related BTB domain containing 2 |
| RHOBTB3 | Rho-related BTB domain containing 3; Rab9-regulated ATPase required for endosome to Golgi transport. Involved in transport vesicle docking at the Golgi complex, |
| RHOC | ras homolog gene family, member C; Regulates a signal transduction pathway linking plasma membrane receptors to the assembly of focal adhesions and actin stress fibers. |
| RHOD | ras homolog gene family, member D; Involved in endosome dynamics. May coordinate membrane transport with the function of the cytoskeleton. Participates in reorganization of actin cytoskeleton (By similarity) |
| RHOH | ras homolog gene family, member H; Negative regulator of hematopoietic progenitor cell proliferation, survival and migration. |
| RHOJ | ras homolog gene family, member J; GTP-binding protein with GTPase activity. Elicits the formation of F-actin-rich structures in fibroblasts and is involved in the regulation of cell morphology (By similarity) |
| RHOQ | ras homolog gene family, member Q |
| RHOU | ras homolog gene family, member U; Acts upstream of PAK1 to regulate the actin cytoskeleton, adhesion turnover and increase cell migration. Stimulates quiescent cells to reenter the cell cycle. |
| RHPN1 | rhophilin, Rho GTPase binding protein 1; Has no enzymatic activity. May serve as a target for Rho, and interact with some cytoskeletal component upon Rho binding or relay a Rho signal to other molecules (By similarity) |
| RHPN2 | Rho GTPase binding protein 2; Binds specifically to GTP-Rho. May function in a Rho pathway to limit stress fiber formation and/or increase the turnover of F-actin structures in the absence of high levels of RhoA activity |
| RILP | Rab interacting lysosomal protein; Rab effector playing a role in late endocytic transport to degradative compartments. Involved in the regulation of lysosomal morphology and distribution. |
| RIMS1 | regulating synaptic membrane exocytosis 1; Rab effector involved in exocytosis. May act as scaffold protein that regulates neurotransmitter release at the active zone. |
| RIN1 | Ras and Rab interactor 1; Ras effector protein, which may serve as an inhibitory modulator of neuronal plasticity in aversive memory formation. Can affect Ras signaling at different levels. |
| RIN2 | Ras and Rab interactor 2; Ras effector protein. May function as an upstream activator and/or downstream effector for RAB5B in endocytic pathway. |
| RIN3 | Ras and Rab interactor 3; Potential Ras effector protein. |
| RIPK4 | receptor-interacting serine-threonine kinase 4; Plays a role in NF-kappa B activation |
| RIT2 | Ras-like without CAAX 2; Binds and exchanges GTP and GDP (By similarity |
| RND1 | Rho family GTPase 1; Lacks intrinsic GTPase activity. Has a low affinity for GDP, and constitutively binds GTP. Controls rearrangements of the actin cytoskeleton. |
| RND2 | Rho family GTPase 2; May be specifically involved in neuronal and hepatic functions. Is a C3 toxin-insensitive member of the Rho subfamily (By similarity) |
| RND3 | Rho family GTPase 3; Binds GTP but lacks intrinsic GTPase activity and is resistant to Rho-specific GTPase-activating proteins |
| RNF115 | ring finger protein 115 |
| RNF139 | ring finger protein 139; Potential tumor suppressor for renal cell carcinoma. Plays a role in mediating ubiquitination. May function as a signaling receptor |
| ROCK1 | Rho-associated, coiled-coil containing protein kinase 1; Phosphorylates and activates DAPK3, which then regulates myosin light chain phosphatase through phosphorylation of MYPT1 thereby regulating the assembly of the actin cytoskeleton, cell migration, invasiveness of tumor cells, smooth muscle contraction and neurite outgrowth. Required for centromere positioning and centromere-dependent exit from mitosis. |
| ROCK2 | Rho-associated, coiled-coil containing protein kinase 2; Regulates the assembly of the actin cytoskeleton. Promotes formation of stress fibers and of focal adhesion complexes. Plays a role in smooth muscle contraction (By similarity) |
| RP1L1 | retinitis pigmentosa 1-like 1 |
| RP2 | retinitis pigmentosa 2 (X-linked recessive); Stimulates the GTPase activity of tubulin, but does not enhance tubulin heterodimerization. Acts as guanine nucleotide dissociation inhibitor for ARL3 |
| RPH3A | rabphilin 3A homolog (mouse); Protein transport. Probably involved with Ras-related protein Rab-3A in synaptic vesicle traffic and/or synaptic vesicle fusion. |
| RPH3AL | rabphilin 3A-like (without C2 domains); Rab GTPase effector involved in the late steps of regulated exocytosis, both in endocrine and exocrine cells (By similarity). |
| RPL7 | ribosomal protein L7; Binds to G-rich structures in 28S rRNA and in mRNAs. Plays a regulatory role in the translation apparatus; inhibits cell-free translation of mRNAs |
| RPS6KB1 | ribosomal protein S6 kinase, 70kDa, polypeptide 1; Phosphorylates specifically ribosomal protein S6 in response to insulin or several classes of mitogens. |
| RPTOR | regulatory associated protein of MTOR, complex 1; Involved in the control of the mammalian target of rapamycin complex 1 (mTORC1) activity which regulates cell growth and survival, and autophagy in response to nutrient and hormonal signals; |
| RRAD | Ras-related associated with diabetes; May play an important role in cardiac antiarrhythmia via the strong suppression of voltage-gated L-type Ca(+2) currents. |
| RRAS | related RAS viral (r-ras) oncogene homolog; Regulates the organization of the actin cytoskeleton |
| RRAS2 | related RAS viral (r-ras) oncogene homolog 2; It is a plasma membrane-associated GTP-binding protein with GTPase activity. |
| RTKN | rhotekin; Mediates Rho signaling to activate NF-kappa-B and may confer increased resistance to apoptosis to cells in gastric tumorigenesis. May play a novel role in the organization of septin structures |
| RTN4 | reticulon 4; Potent neurite growth inhibitor in vitro and plays a role both in the restriction of axonal regeneration after injury and in structural plasticity in the CNS. |
| RUFY1 | RUN and FYVE domain containing 1; Binds phospholipid vesicles containing phosphatidylinositol 3-phosphate and participates in early endosomal trafficking |
| RUNDC3A | RUN domain containing 3A; May act as an effector of RAP2A in neuronal cells (By similarity) |
| RUSC2 | RUN and SH3 domain containing 2 |
| RUVBL2 | RuvB-like 2 (E. coli); Possesses single-stranded DNA-stimulated ATPase and ATP- dependent DNA helicase (5' to 3') activity. |
| S100A9 | S100 calcium binding protein A9; Calcium-binding protein. Has antimicrobial activity towards bacteria and fungi. Important for resistance to invasion by pathogenic bacteria. Up-regulates transcription of genes that are under the control of NF-kappa-B. |
| SCOC | short coiled-coil protein |
| SDCBP | syndecan binding protein (syntenin); Seems to function as an adapter protein. In adherens junctions may function to couple syndecans to cytoskeletal proteins or signaling components. |
| SEC13 | May be involved in protein transport (By similarity) |
| SEC61B | Sec61 beta subunit; Necessary for protein translocation in the endoplasmic reticulum |
| SERTAD1 | SERTA domain containing 1; Acts at E2F-responsive promoters to integrate signals provided by PHD- and/or bromodomain-containing transcription factors. |
| SET | SET nuclear oncogene; Multitasking protein, involved in apoptosis, transcription, nucleosome assembly and histone binding. |
| SFPQ | splicing factor proline/glutamine-rich (polypyrimidine tract binding protein associated); DNA- and RNA binding protein, involved in several nuclear processes. |
| SH2D1A | SH2 domain protein 1A; Inhibitor of the SLAM self-association. Acts by blocking recruitment of the SH2-domain-containing signal-transduction molecule SHP-2 to a docking site in the SLAM cytoplasmic region. |
| SH3BP1 | SH3-domain binding protein 1; Binds differentially to the SH3 domains of certain proteins of signal transduction pathways. |
| SH3D19 | SH3 domain containing 19; May play a role in regulating A disintegrin and metalloproteases (ADAMs) in the signaling of EGFR-ligand shedding. |
| SH3D20 | Rho GTPase activating protein 27; Rho GTPase-activating protein which may be involved in clathrin-mediated endocytosis. |
| SH3RF1 | SH3 domain containing ring finger 1; Acts as a scaffold protein, contributes to Rac-induced signal transduction such as JNKs (MAPK8 and MAPK9) activation and induces apoptosis. |
| SH3RF3 | SH3 domain containing ring finger 3 |
| SHOC2 | RUN domain containing 3A; May act as an effector of RAP2A in neuronal cells (By similarity) |
| SIN3A | SIN3 homolog A, transcription regulator (yeast); Acts as a transcriptional repressor. |
| SLC2A4 | protein interacting with PRKCA 1; Probable adapter protein that bind to and organize the subcellular localization of a variety of membrane proteins containing some PDZ recognition sequence. Involved in the clustering of various receptors, possibly by acting at the receptor internalization level. |
| SMAD2 | SMAD family member 2; Transcriptional modulator activated by TGF-beta and activin type 1 receptor kinase. |
| SMARCA2 | SWI/SNF related, matrix associated, actin dependent regulator of chromatin, subfamily a, member 2; Transcriptional coactivator cooperating with nuclear hormone receptors to potentiate transcriptional activation. |
| SMARCA4 | SWI/SNF related, matrix associated, actin dependent regulator of chromatin, subfamily a, member 4; Transcriptional coactivator cooperating with nuclear hormone receptors to potentiate transcriptional activation. |
| SMARCB1 | SWI/SNF related, matrix associated, actin dependent regulator of chromatin, subfamily b, member 1; Core component of the BAF (hSWI/SNF) complex. This ATP- dependent chromatin-remodeling complex plays important roles in cell proliferation and differentiation, in cellular antiviral activities and inhibition of tumor formation. |
| SMURF1 | SMAD specific E3 ubiquitin protein ligase 1; E3 ubiquitin-protein ligase which accepts ubiquitin from an E2 ubiquitin-conjugating enzyme in the form of a thioester and then directly transfers the ubiquitin to targeted substrates. |
| SMURF2 | SMAD specific E3 ubiquitin protein ligase 2; E3 ubiquitin-protein ligase which accepts ubiquitin from an E2 ubiquitin-conjugating enzyme in the form of a thioester and then directly transfers the ubiquitin to targeted substrates. |
| SNRPE | small nuclear ribonucleoprotein polypeptide E; Appears to function in the U7 snRNP complex that is involved in histone 3'-end processing. Associated with snRNP U1, U2, U4/U6 and U5 |
| SNUPN | snurportin 1; Functions as an U snRNP-specific nuclear import adapter. Involved in the trimethylguanosine (m3G)-cap-dependent nuclear import of U snRNPs. Binds specifically to the terminal m3G-cap U snRNAs |
| SOD2 | superoxide dismutase 2, mitochondrial; Destroys radicals which are normally produced within the cells and which are toxic to biological systems |
| SOS1 | son of sevenless homolog 1 (Drosophila); Promotes the exchange of Ras-bound GDP by GTP |
| SOS2 | son of sevenless homolog 2 (Drosophila); Promotes the exchange of Ras-bound GDP by GTP (By similarity) |
| SPAG8 | sperm associated antigen 8; May play a role in fertility and microtubule formation through interaction with RANBP9 |
| SPAG9 | sperm associated antigen 9; The JNK-interacting protein (JIP) group of scaffold proteins selectively mediates JNK signaling by aggregating specific components of the MAPK cascade to form a functional JNK signaling module. |
| SPRED2 | sprouty-related, EVH1 domain containing 2; Tyrosine kinase substrate that inhibits growth-factor- mediated activation of MAP kinase |
| SRC | v-src sarcoma (Schmidt-Ruppin A-2) viral oncogene homolog (avian) |
| SRGAP1 | SLIT-ROBO Rho GTPase activating protein 1; GTPase-activating protein for RhoA and Cdc42 small GTPases. |
| SSRP1 | structure specific recognition protein 1; Component of the FACT complex, a general chromatin factor that acts to reorganize nucleosomes. The FACT complex is involved in multiple processes that require DNA as a template such as mRNA elongation, DNA replication and DNA repair. |
| STAT1 | signal transducer and activator of transcription 1, 91kDa; Signal transducer and activator of transcription that mediates signaling by interferons (IFNs). |
| STAT3 | signal transducer and activator of transcription 3 (acute-phase response factor); Transcription factor that binds to the interleukin-6 (IL-6)-responsive elements identified in the promoters of various acute-phase protein genes. |
| STAU1 | staufen, RNA binding protein, homolog 1 (Drosophila); Binds double-stranded RNA (regardless of the sequence) and tubulin. May play a role in specific positioning of mRNAs at given sites in the cell by cross-linking cytoskeletal and RNA components, and in stimulating their translation at the site |
| STX1A | Potentially involved in docking of synaptic vesicles at presynaptic active zones. May play a critical role in neurotransmitter exocytosis |
| STX4 | syntaxin 4; Plasma membrane t-SNARE that mediates docking of transport vesicles. |
| SYNE1 | spectrin repeat containing, nuclear envelope 1; Involved in the maintenance of nuclear organization and structural integrity. Probable anchoring protein which theters the nucleus to the cytoskeleton. Connects nuclei to the cytoskeleton by interacting with the nuclear envelope and with F-actin in the cytoplasm |
| SYNJ2 | synaptojanin 2; Inositol 5-phosphatase which may be involved in distinct membrane trafficking and signal transduction pathways. May mediate the inhibitory effect of Rac1 on endocytosis |
| SYTL1 | synaptotagmin-like 1; May play a role in vesicle trafficking (By similarity). Binds phosphatidylinositol 3,4,5-triphosphate. Acts as a RAB27A effector protein and may play a role in cytotoxic granule exocytosis in lymphocytes (By similarity) |
| SYTL2 | synaptotagmin-like 2; Isoform 1 acts as a RAB27A effector protein and plays a role in cytotoxic granule exocytosis in lymphocytes. |
| SYTL3 | synaptotagmin-like 3; May act as Rab effector protein and play a role in vesicle trafficking. Binds phospholipids in the presence of calcium ions (By similarity) |
| SYTL4 | synaptotagmin-like 4; Modulates exocytosis of dense-core granules and secretion of hormones in the pancreas and the pituitary. Interacts with vesicles containing negatively charged phospholipids in a Ca(2+)-independent manner (By similarity) |
| SYTL5 | synaptotagmin-like 5; May act as Rab effector protein and play a role in vesicle trafficking. Binds phospholipids |
| TAF2E | TAF6 RNA polymerase II, TATA box binding protein (TBP)-associated factor, 80kDa; TAFs are components of the transcription factor IID (TFIID) complex, PCAF histone acetylase complex and TBP-free TAFII complex (TFTC). |
| TBC1D3F | TBC1 domain family, member 3F; Acts as a GTPase activating protein for RAB5. Does not act on RAB4 or RAB11 (By similarity) |
| TBC1D3G | TBC1 domain family, member 3G; Acts as a GTPase activating protein for RAB5. |
| TBCD | tubulin folding cofactor D; Tubulin-folding protein; involved in the first step of the tubulin folding pathway. |
| TBXA2R | thromboxane A2 receptor; Receptor for thromboxane A2 (TXA2), a potent stimulator of platelet aggregation. |
| TCEB3 | transcription elongation factor B (SIII), polypeptide 3 (110kDa, elongin A); SIII, also known as elongin, is a general transcription elongation factor that increases the RNA polymerase II transcription elongation past template-encoded arresting sites. |
| TEC | tec protein tyrosine kinase |
| TES | testis derived transcript (3 LIM domains); Scaffold protein that may play a role in cell adhesion, cell spreading and in the reorganization of the actin cytoskeleton. |
| TFRC | Cellular uptake of iron occurs via receptor-mediated endocytosis of ligand-occupied transferrin receptor into specialized endosomes. |
| TGM2 | transglutaminase 2 (C polypeptide, protein-glutamine-gamma-glutamyltransferase); Catalyzes the cross-linking of proteins and the conjugation of polyamines to proteins |
| TIAM1 | T-cell lymphoma invasion and metastasis 1; Modulates the activity of RHO-like proteins and connects extracellular signals to cytoskeletal activities. Acts as a GDP- dissociation stimulator protein that stimulates the GDP-GTP exchange activity of RHO-like GTPases and activates them. |
| TJP1 | tight junction protein 1 (zona occludens 1); The N-terminal may be involved in transducing a signal required for tight junction assembly, |
| TLR2 | toll-like receptor 2; Cooperates with LY96 to mediate the innate immune response to bacterial lipoproteins and other microbial cell wall components. |
| TLR9 | toll-like receptor 9; Participates in the innate immune response to microbial agents. Detects the unmethylated cytidine-phosphate-guanosine (CpG) motifs present in bacterial DNA. |
| TM9SF2 | transmembrane 9 superfamily member 2; In the intracellular compartments, may function as a channel or small molecule transporter |
| TM9SF4 | transmembrane 9 superfamily protein member 4 |
| TMED10 | transmembrane emp24-like trafficking protein 10 (yeast); Involved in vesicular protein trafficking |
| TMED2 | transmembrane emp24 domain trafficking protein 2; Could have a role in the budding of coatomer-coated and other species of coated vesicles. |
| TMF1 | Potential coactivator of the androgen receptor. Mediates STAT3 degradation. |
| TNF | tumor necrosis factor (TNF superfamily, member 2); Cytokine that binds to TNFRSF1A/TNFR1 and TNFRSF1B/TNFBR. It is mainly secreted by macrophages and can induce cell death of certain tumor cell lines. |
| TNFAIP1 | tumor necrosis factor, alpha-induced protein 1 (endothelial); Enhances the PCNA-dependent DNA polymerase delta activity (By similarity) |
| TNFRSF10B | tumor necrosis factor receptor superfamily, member 10b; Receptor for the cytotoxic ligand TNFSF10/TRAIL. The adapter molecule FADD recruits caspase-8 to the activated receptor. The resulting death-inducing signaling complex (DISC) performs caspase-8 proteolytic activation which initiates the subsequent cascade of caspases (aspartate-specific cysteine proteases) mediating apoptosis. Promotes the activation of NF- kappa-B |
| TNFRSF10C | tumor necrosis factor receptor superfamily, member 10c, decoy without an intracellular domain; Receptor for the cytotoxic ligand TRAIL. |
| TNFRSF12A | tumor necrosis factor receptor superfamily, member 12A; Receptor for TNFSF12/TWEAK. Weak inducer of apoptosis in some cell types. |
| TNFRSF1A | tumor necrosis factor receptor superfamily, member 1A; Receptor for TNFSF2/TNF-alpha and homotrimeric TNFSF1/lymphotoxin-alpha. The adapter molecule FADD recruits caspase-8 to the activated receptor. |
| TNIK | TRAF2 and NCK interacting kinase; Serine/threonine kinase that acts as an essential activator of the Wnt signaling pathway. |
| TNK2 | tyrosine kinase, non-receptor, 2; Downstream effector of CDC42 which mediates CDC42- dependent cell migration via phosphorylation of BCAR1. Binds to both poly- and mono-ubiquitin and regulates ligand-induced degradation of EGFR. |
| TNPO1 | transportin 1; Functions in nuclear protein import as nuclear transport receptor. Serves as receptor for nuclear localization signals (NLS) in cargo substrates. |
| TNPO2 | transportin 2; Probably functions in nuclear protein import as nuclear transport receptor. Serves as receptor for nuclear localization signals (NLS) in cargo substrates. |
| TP73 | tumor protein p73; Acts as a tumor suppressor in many tumor types; induces growth arrest or apoptosis depending on the physiological circumstances and cell type. |
| TPM2 | tropomyosin 2 (beta); Binds to actin filaments in muscle and non-muscle cells. Plays a central role, in association with the troponin complex, in the calcium dependent regulation of vertebrate striated muscle contraction. |
| TRAF3 | TNF receptor-associated factor 3; Adapter protein and signal transducer that links members of the tumor necrosis factor receptor family to different signaling pathways by association with the receptor cytoplasmic domain and kinases. |
| TRIM23 | tripartite motif-containing 23; Not known, the C-terminus can act as an allosteric activator of the cholera toxin catalytic subunit |
| TRIO | triple functional domain (PTPRF interacting); Promotes the exchange of GDP by GTP. Together with leukocyte antigen-related (LAR) protein, it could play a role in coordinating cell-matrix and cytoskeletal rearrangements necessary for cell migration and cell growth |
| TRIP10 | thyroid hormone receptor interactor 10; Required for translocation of GLUT4 to the plasma membrane in response to insulin signaling (By similarity). |
| TRIP13 | thyroid hormone receptor interactor 13 |
| TRPC1 | transient receptor potential cation channel, subfamily C, member 1; Thought to form a receptor-activated non-selective calcium permeant cation channel. |
| TSC2 | tuberous sclerosis 2; In complex with TSC1, inhibits the nutrient-mediated or growth factor-stimulated phosphorylation of S6K1 and EIF4EBP1 by negatively regulating mTORC1 signaling. |
| TTC1 | tetratricopeptide repeat domain 1 |
| TUBA1A | tubulin, alpha 1a; Tubulin is the major constituent of microtubules. It binds two moles of GTP, one at an exchangeable site on the beta chain and one at a non-exchangeable site on the alpha-chain |
| TUBA4A | tubulin, alpha 4a; Tubulin is the major constituent of microtubules. It binds two moles of GTP, one at an exchangeable site on the beta chain and one at a non-exchangeable site on the alpha-chain (By similarity) |
| TUBB | tubulin, beta |
| TUBB2A | tubulin, beta 2A; Tubulin is the major constituent of microtubules. It binds two moles of GTP, one at an exchangeable site on the beta chain and one at a non-exchangeable site on the alpha-chain (By similarity) |
| TUBG1 | tubulin, gamma 1; Tubulin is the major constituent of microtubules. Gamma tubulin is found at microtubule organizing centers (MTOC) such as the spindle poles or the centrosome, suggesting that it is involved in the minus-end nucleation of microtubule assembly |
| UBB | ubiquitin B |
| UBC | ubiquitin C |
| UBXN11 | UBX domain protein 11; May be involved in the reorganization of actin cytoskeleton mediated by RND1, RND2 AND RND3. |
| UNC119 | unc-119 homolog (C. elegans); May play a role in the mechanism of photoreceptor neurotransmitter release through the synaptic vesicle cycle |
| UNC13D | unc-13 homolog D (C. elegans); Plays a role in cytotoxic granule exocytosis in lymphocytes. Required for both granule maturation and granule docking and priming at the immunologic synapse. |
| USO1 | USO1 homolog, vesicle docking protein (yeast); General vesicular transport factor required for intercisternal transport in the Golgi stack; it is required for transcytotic fusion and/or subsequent binding of the vesicles to the target membrane. |
| USP6NL | USP6 N-terminal like; Acts as a GTPase-activating protein for RAB5A. Involved in receptor trafficking. In complex with EPS8 inhibits internalization of EGFR |
| VAV1 | vav 1 guanine nucleotide exchange factor; Couples tyrosine kinase signals with the activation of the Rho/Rac GTPases, thus leading to cell differentiation and/or proliferation |
| VAV2 | vav 2 guanine nucleotide exchange factor; Guanine nucleotide exchange factor for the Rho family of Ras-related GTPases. Plays an important role in angiogenesis. Its recruitement by phosphorylated EPHA2 is critical for EFNA1-induced RAC1 GTPase activation and vascular endothelial cell migration and assembly (By similarity) |
| VAV3 | vav 3 guanine nucleotide exchange factor; Exchange factor for GTP-binding proteins RhoA, RhoG and, to a lesser extent, Rac1. Binds physically to the nucleotide-free states of those GTPases. Plays an important role in angiogenesis. |
| VIM | vimentin; Vimentins are class-III intermediate filaments found in various non-epithelial cells, especially mesenchymal cells |
| VPS13C | vacuolar protein sorting 13 homolog C |
| VPS24 | vacuolar protein sorting 24 homolog (S. cerevisiae); Probable core component of the endosomal sorting required for transport complex III (ESCRT-III) which is involved in multivesicular bodies |
| VPS45 | vacuolar protein sorting 45 homolog (S. cerevisiae); May play a role in vesicle-mediated protein trafficking from the Golgi stack through the trans-Golgi network |
| VPS4A | vacuolar protein sorting 4 homolog A (S. cerevisiae); Involved in late steps of the endosomal multivesicular bodies (MVB) pathway. Recognizes membrane-associated ESCRT-III assemblies and catalyzes their disassembly, possibly in combination with membrane fission. |
| VRK1 | vaccinia related kinase 1; Serine/threonine kinase that phosphorylates 'Thr-18' of p53/TP53 and may thereby prevent the interaction between p53/TP53 and MDM2 |
| VRK2 | vaccinia related kinase 2; Probable serine/threonine kinase (By similarity) |
| VRK3 | vaccinia related kinase 3; Probable serine/threonine kinase (By similarity) |
| WAS | Wiskott-Aldrich syndrome (eczema-thrombocytopenia); Effector protein for Rho-type GTPases, providing a link with the Arp2/3 complex that regulates the structure and dynamics of the actin cytoskeleton. Important for efficient actin polymerization. |
| WASF1 | WAS protein family, member 1; Downstream effector molecules involved in the transmission of signals from tyrosine kinase receptors and small GTPases to the actin cytoskeleton |
| WASF2 | WAS protein family, member 2; Downstream effector molecules involved in the transmission of signals from tyrosine kinase receptors and small GTPases to the actin cytoskeleton (By similarity) |
| WASL | Wiskott-Aldrich syndrome-like; Regulates actin polymerization by stimulating the actin- nucleating activity of the Arp2/3 complex. Binds to HSF1/HSTF1 and forms a complex on heat shock promoter elements (HSE) that negatively regulates HSP90 expression |
| WDR44 | Downstream effector for RAB11. May be involved in vesicle recycling (By similarity) |
| XPO1 | exportin 1 (CRM1 homolog, yeast); Mediates the nuclear export of cellular proteins (cargos) bearing a leucine-rich nuclear export signal (NES) and of RNAs. |
| XPO4 | exportin 4; Mediates the nuclear export of proteins (cargos) with broad substrate specificity. |
| XPO5 | exportin 5; Mediates the nuclear export of proteins bearing a double-stranded RNA binding domain (dsRBD) and double-stranded RNAs (cargos). XPO5 in the nucleus binds cooperatively to the RNA and to the GTPase Ran in its active GTP-bound form. |
| XPO6 | exportin 6; Mediates the nuclear export of actin and profilin-actin complexes in somatic cells |
| XPO7 | exportin 7; Mediates the nuclear export of proteins (cargos) with broad substrate specificity. In the nucleus binds cooperatively to its cargo and to the GTPase Ran in its active GTP-bound form. |
| XPOT | exportin, tRNA (nuclear export receptor for tRNAs); Mediates the nuclear export of aminoacylated tRNAs. |
| YWHAB | tyrosine 3-monooxygenase/tryptophan 5-monooxygenase activation protein, beta polypeptide; Adapter protein implicated in the regulation of a large spectrum of both general and specialized signaling pathway. |
| YWHAE | tyrosine 3-monooxygenase/tryptophan 5-monooxygenase activation protein, epsilon polypeptide; Adapter protein implicated in the regulation of a large spectrum of both general and specialized signaling pathway. |
| YWHAH | tyrosine 3-monooxygenase/tryptophan 5-monooxygenase activation protein, eta polypeptide; Adapter protein implicated in the regulation of a large spectrum of both general and specialized signaling pathway. |
| YWHAQ | tyrosine 3-monooxygenase/tryptophan 5-monooxygenase activation protein, theta polypeptide; Adapter protein implicated in the regulation of a large spectrum of both general and specialized signaling pathway. |
| YWHAZ | tyrosine 3-monooxygenase/tryptophan 5-monooxygenase activation protein, zeta polypeptide; Adapter protein implicated in the regulation of a large spectrum of both general and specialized signaling pathway. |
| ZBTB12 | zinc finger and BTB domain-containing 12; may be involved in transcription |
| ZFYVE20 | zinc finger, FYVE domain containing 20; Rab4/Rab5 effector protein acting in early endocytic membrane fusion and membrane trafficking of recycling endosomes. |
| ZHX2 | zinc fingers and homeoboxes 2; Acts as a transcriptional repressor |
| ZNF212 | zinc finger protein 212; May be involved in transcriptional regulation |
| ZNF709 | zinc finger protein 709; May be involved in transcriptional regulation |
|  |  |
|  |  |
|  |  |
