# Supplementary material for: Topological and Functional Properties of the Small GTPases Protein Interaction Network
Source: PLoS One. 2012 Sep 13;7(9):e44882. doi: 10.1371/journal.pone.0044882 (PMC3441499; doi:10.1371/journal.pone.0044882)
Supplement: Table S3 — Proteins present in multiple small GTPase networks. (DOCX) [file pone.0044882.s004.docx]

| **Table S3. Proteins present in multiple small GTPase networks** | | |
| --- | --- | --- |
| **Networks** | **Role** | **Gene Symbol** |
| Rho, Ras | Actin | FHOD1, FLNA |
|  | Adaptor | ITSN1, NCK1 |
|  | Apoptosis | FADD, FAS |
|  | Effector | ABL2, RALBP1 |
|  | GAP | ARHGAP29, RACGAP1, RAV1, GMIP |
|  | GEF | ARHGEF2, RAP1GDS1, RAPGEF1, RASGRF1, RGL2, VAV1 |
|  | GTPase | RHOD, HRAS |
|  | Heat shock | HSPA1A |
|  | Kinase | PIK3R1, PRKACA, PRKCB, PRKCZ, MAPK8 |
|  | Lipid | FNTA, ICMT, PGGT1B |
|  | NTP synthesis | NME1 |
|  | Phosphatase | PTPN1 |
|  | Receptor | GNA12, GRB2, TLR2 |
|  | Ubiquitin | SMURF2, UBC |
| Rho, Rab | Actin | ACTB |
|  | GAP | TBC1D3F |
|  | GDI | GDI1 |
|  | GTPase | RAB35, RAB9B |
|  | Kinase | AKT1 |
|  | Lipid | OCRL |
|  | Receptor | PLN3 |
|  | Tubulin | TUBA4A |
| Ras, Rab | Adaptor | AP1G1 |
|  | GEF/Effector | RIN1 |
|  | GTPase | RAB8B |
|  | Phosphatase | PPP2R1B, GAP, RASA1 |
|  | Receptor | AGTR1 |
|  | Signaling | HGS |
|  | Tumor supp | TSC2 |
| Rab, Arf | Adaptor | GGA1, GGA2, GGA3, SLC2A4 |
|  | Effector | RAB11FIP3 |
|  | Golgi | GCC2, GOLG1 |
|  | Tubulin | TUBA1A |
| Rho, Arf | Cytoskeleton | EZR |
|  | Effector | ARFIP1, ARFIP2, ARL2BP |
|  | GAP | ARHGAP21 |
|  | GTPase | CDC42 |
| Ras, Arf | GTPase | ARF1, ARF6, RALA |
|  | Signaling | CALM1 |
|  | Vesicle traffic | EXOC5 |
| Ran, Arf | GAP | ASAP2 |
| Rho, Ran | Phosphatase | PPP2R2B |
|  | Receptor | AR |
| Arf, Rho, Rab | Signaling | CAV1 |
| Arf, Ras, Rho | Kinase | PRKC1 |
|  | Phosphatase | PPP2CA |
|  | Phospholipase | PLD1 |
|  | Receptor | INSR |
| Ras, Rho, Rab | Lipid | RABAC1 |
|  | Receptor | MTNR1A |
| Arf, Rho, Rab, Ras | GDI-like | PDE6D |
