# Supplementary material for: Topological and Functional Properties of the Small GTPases Protein Interaction Network
Source: PLoS One. 2012 Sep 13;7(9):e44882. doi: 10.1371/journal.pone.0044882 (PMC3441499; doi:10.1371/journal.pone.0044882)
Supplement: Table S4 — Interactions of proteins present in multiple small GTPase networks. (DOCX) [file pone.0044882.s005.docx]

**Table S4. Interactions of proteins present in multiple small GTPase networks**

| **Networks** | **Role** | **Gene symbol** | **Neighbors** |
| --- | --- | --- | --- |
|  |  |  |  |
| Arf, Rho, Rab | Signaling | CAV1 | FLNA, FYN, HRAS, INSR, NOS2, PLD1, PRKACA, RAB27B, RAC1, RASA1, RHOA, SOS1, SRC, TRPC1 |
| Arf, Ras, Rho | Kinase | PRKC1 | CDC42, GAPDH, HRAS, IRAK1, MAP2K1,PARD6B, PARG6G, PRKCZ, RAB2A, RAB4A, RAC1, RHOQ, SRC |
|  | Phosphatase | PPP2CA | ARL2, BCL2, PPP2R2C, PPP2R5D, RHOB, RRAS, TBCD |
|  | Phospholipase | PLD1 | ACTA1, ACTB, ARF1, ARF6, VAV1, CDC42, PIP5K1A, PKN1, PRKCA, RAC1, RALA, RHOA |
|  | Receptor | INSR | ARF1, CAV1, GRB2, HRAS, PIK3R1, PTPN1, RAF1, RASA1, RHOB, SRC, VAV1 |
| Ras, Rho, Rab | Lipid | RABAC1 | GDI1, HRAS, OBOE,RAB1A, RAB3A, RAB4A, RAB5A, RAB5C, RAB6A, RAB7A, RAB17, RAB22A, RAB33A, RAP1A, RHOA, RRAS2 |
|  | Receptor | MTNR1A | FLNA, RAB10, RAC1, RAP1A |
| Arf, Rho, Rab, Ras | GDI-like | PDE6D | ARL2, ARL3, HRAS, RAB8A, RAB13, RAP1A, RAP2B, RASA1, RHOA, RHOB, RND1 |
